# Human LFA-1 governs T cell immune surveillance of the skin

**DOI:** 10.1126/sciimmunol.adz8360

**Published:** 2026-02-27

**Authors:** Ahmad Yatim, Leila Youssefian, Aida Idani, Haralambos Mourelatos, Nasrin Alipour Olyaei, Laleh Habibi, Jessica N. Peel, Maira Gaballa, Colin Kim, Danyel Lee, Corentin Le Floc’h, Yi-Hao Chan, Marta Martin-Fernandez, Léo Pelletier, Koji Nakajima, Antoine Fayand, Mana Momenilandi, Yoann Seeleuthner, Matthieu Chaldebas, Amir Hossein Saeidian, Hamidreza Mahmoudi, Neda Nikbakht, Caroline Deswarte, Nesrine Kerrouche, Houria Sahel, Nima Parvaneh, Banafshe Tamizifar, Roya Sherkat, Johan Chanal, Alireza Firooz, Zahra Alizadeh, Lazaro Lorenzo-Diaz, Alice-Andrée Mariaggi, Masato Ogishi, Antoine Diep, Boualem Hammadi, Nacim Kerrouche, Darawan Rinchai, Sahar Sedighzadeh, Soophia Mehrjooy, Seyed Mohammad Akrami, Cedric Lenormand, Mirjana Radosavljevic, Jérémie Rosain, Shen-Ying Zhang, Bertrand Boisson, Peng Zhang, Stéphanie Boisson-Dupuis, James G. Krueger, Jean-Luc Prétet, Tim Waterboer, Zahra Pourpak, Mohammad Reza Fazlollahi, Laurent Abel, Dusan Bogunovic, Mohammad Shahrooei, Aurélie Cobat, Raphael Carapito, Anne Molitor, Hassan Vahidnezhad, Vivien Béziat, Seiamak Bahram, Emmanuelle Jouanguy, Jean-Laurent Casanova

**Affiliations:** 1 St. Giles Laboratory of Human Genetics of Infectious Diseases, Rockefeller Branch, Rockefeller University, New York, NY, USA.; 2 Laboratory of Human Genetics of Infectious Diseases, Necker Branch, INSERM UMR 1163, Necker Hospital for Sick Children, Paris, France.; 3 Paris Cité University, Imagine Institute, Paris, France.; 4 Department of Pathol-ogy, Cytogenetics Laboratory, City of Hope National Medical Center, Irwindale, CA, USA.; 5 Laboratoire d’ImmunoRhumatologie Moléculaire, plateforme GENOMAX, INSERM UMR_S 1109, Faculté de Médecine, Fédération Hospitalo-Universitaire OMICARE, Fédération de Médecine Translationnelle de Strasbourg (FMTS), Institut Thématique Interdisciplinaire TRANSPLANTEX NG, Université de Strasbourg, Strasbourg, France.; 6 Weill Cornell/Rockefeller/Memorial Sloan Kettering Tri-Institutional MD-PhD Program, New York, NY, USA.; 7 Department of Medical Genetics, School of Medicine, Tehran University of Medical Sciences, Tehran, Iran.; 8 Immunology, Asthma and Allergy Research Institute, Tehran University of Medical Sciences, Tehran, Iran.; 9 Department of Pediatrics and Center for Genetic Errors of Immunity, Columbia University Medical Center, New York, NY, USA.; 10 Rare Diseases Research Institute (IIER), Instituto de Salud Carlos III, Madrid, Spain.; 11 Department of Molecular and Human Genetics, Baylor College of Medicine, Houston, TX, USA.; 12 Department of Dermatology, Razi Hospital, Tehran University of Medical Sciences, Tehran, Iran.; 13 Department of Dermatology and Cutaneous Biology, Sidney Kimmel Cancer Center, Thomas Jefferson University, Philadelphia, PA, USA.; 14 Établissement Public de Santé de Proximité de Cheraga, Polyclinique de Cheraga, Department of Dermatology, Algiers, Algeria.; 15 Department of Dermatology, CHU Bab El Oued, Faculty of Medicine, University of Algiers 1, Algiers, Algeria.; 16 Division of Allergy and Clinical Immunology, Department of Pediatrics, Tehran University of Medical Sciences, Tehran, Iran.; 17 Dermatology Clinic, Tehran, Iran.; 18 Immunodeficiency Diseases Research Center, Isfahan University of Medical Sciences, Isfahan, Iran.; 19 Service de Dermatologie, Hôpital Cochin-Port Royal, Paris, France.; 20 Center for Research and Training in Skin Disease and Leprosy, Tehran University of Medical Sciences, Tehran, Iran.; 21 Children’s Medical Center, Pediatrics Center of Excellence, Tehran University of Medical Sciences, Tehran, Iran.; 22 Laboratory of Virology, Assistance Publique-Hôpitaux de Paris (AP-HP), Cochin Hospital, Paris, France.; 23 Department of Immunology, Saint-Louis Hospital, Paris, France.; 24 General Chemistry Laboratory, Department of Clinical Chemistry, APHP, Necker Hospital for Sick Children, Paris, France.; 25 Dr. Shahrooei Lab, Tehran, Iran.; 26 Dermatologic Clinic, University of Strasbourg, Strasbourg University Hospital, Strasbourg, France.; 27 Service d’Immunologie Bi-ologique, Plateau Technique de Biologie, Pôle de Biologie, Nouvel Hôpital Civil, Strasbourg, France.; 28 Study Center for Primary Immunodeficiencies, Necker Hospital for Sick Children, AP-HP, Paris, France.; 29 Laboratory of Investigative Dermatology, Rockefeller University, New York, NY, USA.; 30 Université Marie et Louis Pasteur, CHU Besançon, CNRS, Chrono-environnement (UMR 6249), Besançon, France.; 31 Division of Infections and Cancer Epidemiology, German Cancer Research Center (DKFZ), Heidelberg, Germany.; 32 Center for Applied Genomics, Children’s Hospital of Philadelphia, Philadelphia, PA, USA.; 33 Division of Human Genetics, Children’s Hospital of Philadelphia, Philadelphia, PA, USA.; 34 Department of Pediatrics, Perelman School of Medicine, University of Pennsylvania, Philadelphia, PA, USA.; 35 Department of Dermatology, Perelman School of Medicine, University of Pennsylvania, Philadelphia, PA, USA.; 36 Department of Pediatrics, Necker Hospital for Sick Children, Paris, France.; 37 Howard Hughes Medical Institute, New York, NY, USA.

## Abstract

The human integrin lymphocyte function–associated antigen 1 (LFA-1; αLβ2) is broadly expressed on leukocytes and involved in various intercellular adhesions. We report complete LFA-1 deficiency because of inherited αL (CD11a) deficiency in otherwise healthy adults of various ancestries with skin lesions due to commensal papillomaviruses. The patients had no history of invasive infections characteristic of children with inherited deficiency of β2 (CD18), which forms heterodimers with αL, αM (CD11b), αX (CD11c), or αD (CD11d). The development and function of leukocyte subsets are largely preserved in the absence of LFA-1. However, the transendothelial migration of skin-tropic cutaneous lymphocyte antigen (CLA)^+^ memory T cells is severely impaired, resulting in their selective sequestration in the blood. Conversely, alternative integrins mediate the extravasation of other leukocytes, including other T cell subsets, to other tissues. Human LFA-1 is required for steady-state T cell homing to the skin and control of papillomaviruses but is otherwise largely redundant. Integrin-mediated T cell compartmentalization is thus essential for organ-selective immune surveillance.

## INTRODUCTION

Human papillomaviruses (HPVs) form a group of more than 200 viruses that infect only keratinocytes in cutaneous and mucosal stratified epithelia. Commensal HPVs, mostly from the β genus, are ubiquitous in healthy skin and account for a large proportion of the human skin virome (1–3). The continuous clearance of infected keratinocytes renders β-HPV infection clinically invisible and protects against virus-related warts and nonmelanoma skin cancer (NMSC) (4, 5). However, this control can be compromised in patients with epidermodysplasia verruciformis (EV), a group of rare single-gene inborn errors of immunity (IEI) that selectively impair skin immunity to commensal HPVs. EV is characterized by lifelong disseminated flat warts caused by the uncontrolled expansion of β-HPVs (6, 7). In addition to substantial morbidity, about 50% of patients develop NMSC in areas of skin exposed to ultraviolet (UV) radiation (8). Beyond β-HPVs, a dysregulation of the cutaneous viral flora is observed in patients with EV (9), including an expansion of skin commensal polyomaviruses and a predisposition to polyomavirus-driven Merkel cell carcinoma (10–12), suggesting a broad impairment of skin immunity to commensal viruses.

EV has long been considered a prototypical disease for studying skin immunity to viruses, including oncogenic HPVs (13, 14). Inherited deficiencies of *TMC6* (EVER1), *TMC8* (EVER2), and *CIB1* account for about half of all isolated EV cases (15, 16), but the cellular basis of the disease remains unresolved. The essential role of T cells in protective immunity to commensal HPVs has been documented through studies of syndromic forms of EV in humans (17–19) and, more recently, in studies of mice infected with mouse papillomavirus (MmuPV1) (4, 5). However, not all inborn errors of T cell immunity confer a predisposition to β-HPV expansion and NMSC (20), suggesting that EV-associated T cell defects may specifically compromise a core mechanism of the immune surveillance of commensal viruses in the skin. Given that about half of patients with EV remain without genetic etiology, we aimed to discover and mechanistically characterize a genetic cause of EV in these patients, which might reveal essential components of T cell–mediated skin defense.

## RESULTS

### Four probands with rare biallelic *ITGAL* variants

**Fig. 1.**
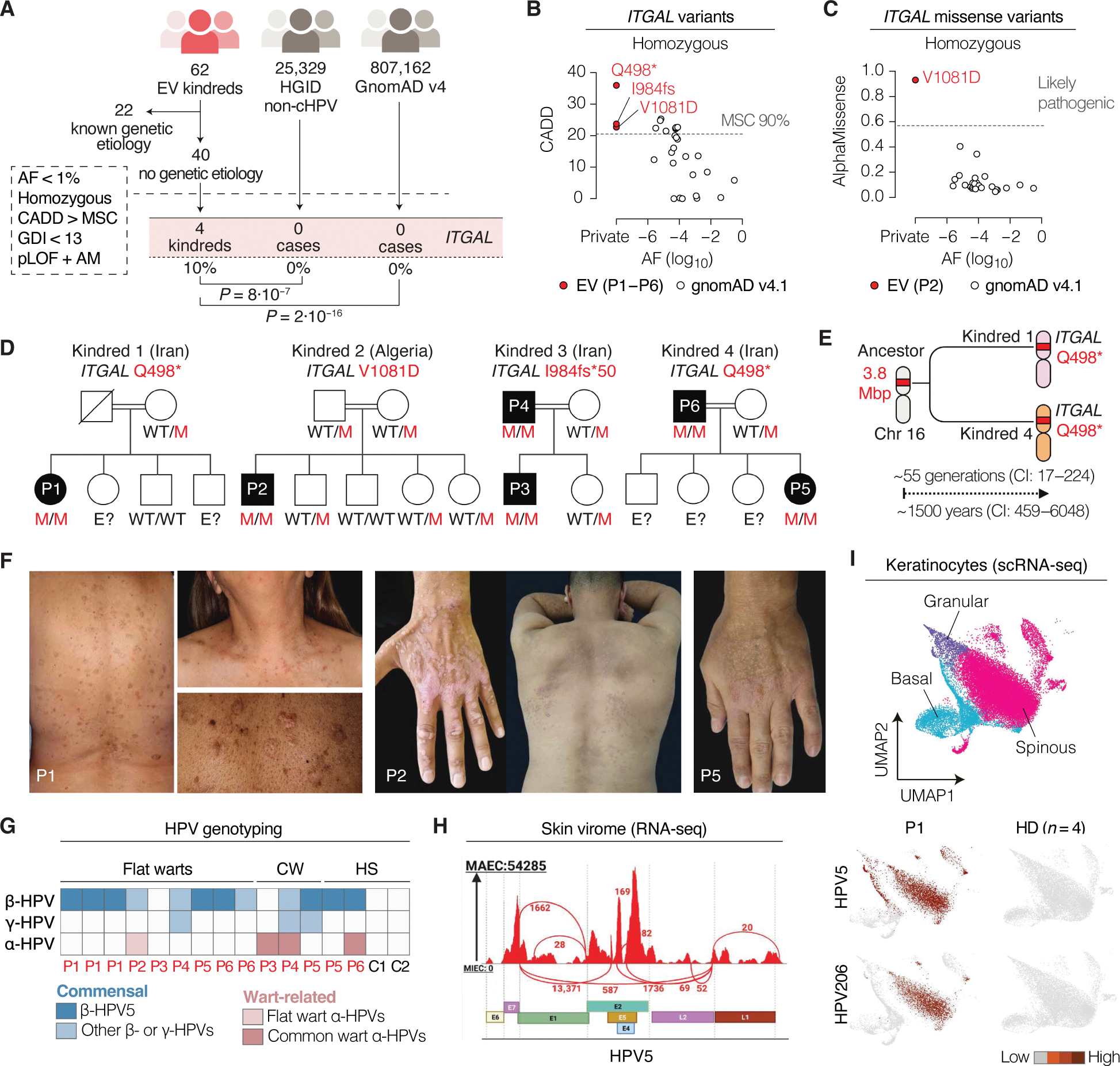
Rare biallelic *ITGAL* variants in patients with isolated EV. (**A**) Strategy for identifying biallelic, predicted deleterious variants enriched in the EV cohort. See Materials and Methods for variant filtering strategy. HGID, Laboratory of Human Genetics of Infectious Diseases; non-cHPV, noncutaneous HPV. (**B** and **C**) Homozygous *ITGAL* variants in gnomAD v4.1 (white) or EV (red), plotted by (B) CADD score versus allele frequency (AF) for all nonsynonymous variants and by (C) AlphaMissense score versus AF for missense variants. (**D**) Pedigree showing segregation of *ITGAL* variants in the four kindreds. Black symbols, individuals with EV; M, mutated; E?, unknown. (**E**) Estimated age of the most recent common ancestor determined by ESTIAGE. CI, confidence interval. (**F**) Photographs of the patients’ lesions. (**G**) HPV genotyping in skin biopsies from patients (P, *N* = 14) and controls (C, *N* = 2). CW, common warts; HS, healthy skin. (**H**) Whole-transcriptome sequencing–based detection of viral transcripts in skin lesions from P1. MAEC, maximum exon coverage. (**I**) scRNA-seq of keratinocytes from P1 and healthy controls (*N* = 4). Top: Uniform Manifold Approximation and Projection (UMAP) showing keratinocyte clustering. Bottom: Feature plots of HPV transcript levels.

In our cohort of 62 unrelated kindreds with EV, 22 probands carried deleterious variants of known EV-causing genes. The genetic cause in the other 40 kindreds remained unexplained. An unbiased genomewide search in these 40 probands (Fig. 1A) identified 46 rare biallelic variants predicted to be deleterious in 44 genes (table S1). None of these variants were found in known IEI-causing genes (21). *ITGAL* was the only gene recurrently mutated, with homozygous variants detected in four unrelated kindreds (Fig. 1A and fig. S1A). No such homozygous *ITGAL* variants were found in our control database of 25,329 individuals with other infectious diseases (table S2), indicating a strong and specific association between these *ITGAL* genotypes and EV. Moreover, analysis of *ITGAL* variants from the gnomAD dataset (v4.1, comprising 807,162 individuals) revealed no homozygous *ITGAL* variants meeting these criteria in the general population (Fig. 1, B and C). The EV cohort was therefore highly enriched in individuals homozygous for predicted deleterious *ITGAL* variants (10%, 4 of 40), compared with patients with other infectious diseases (0 of 25,329) and with the general population (0 of 807,162), with corrected odds ratios of 442 (*P* = 8 × 10^−7^) and 1.9 × 10^5^ (*P* = 2 × 10^−16^), respectively (fig. S1B and table S2). Overall, these findings strongly suggest that autosomal recessive (AR) *ITGAL* deficiency is a genetic etiology of EV.

### Familial segregation of biallelic *ITGAL* variants with EV

Clinical and familial data were obtained for the four probands with candidate *ITGAL* genotypes (Fig. 1D). Six patients (P1 to P6) from four unrelated kindreds and two countries (Algeria and Iran) had isolated EV, including five adults (aged 33 to 78 years) and one adolescent (aged 14 years). Familial segregation was consistent with an AR pattern of inheritance with complete penetrance (Fig. 1D). Three different *ITGAL* variants were identified (table S3): Q498* (P1, P5, and P6), I984Sfs*50 (P3 and P4), and V1081D (P2). A founder effect accounts for the recurrence of Q498* in kindreds 1 and 4, with the most recent common ancestor estimated to have lived 55 generations (~1500 years) ago (Fig. 1E). The patients had widespread and persistent cutaneous flat warts characteristic of EV (P1 to P6) (Fig. 1F). P1 and P6 developed cutaneous squamous and basal cell carcinomas after the age of 50 years, consistent with the high risk of late-onset NMSC in patients with EV (8). In addition, P3 to P6 suffered from episodes of common warts (fig. S1, C and D), as occasionally seen in patients with isolated EV (7). HPV genotyping identified β- and γ-HPVs in 86% (12 of 14) and 21% (3 of 14) of skin biopsies from the patients, respectively, whereas α-HPVs were detected in 27% (4 of 14) (Fig. 1G and table S4). Whole transcriptome–based profiling of the skin virome showed high levels of β-HPV-5 replication in the lesions of P1 (Fig. 1H). Moreover, single-cell RNA sequencing (scRNA-seq) identified β-HPV-5 and β-HPV-206 transcripts in keratinocytes from a skin lesion of P1, but not in healthy control epidermis (*n* = 4), indicating that a substantial proportion of keratinocytes displayed persistent β-HPV infection, a characteristic feature of EV (Fig. 1I). None of the six patients had a history of other severe infectious diseases, although they had documented infections with various pathogens, as demonstrated by serological testing (fig. S1E and table S5). All six patients, therefore, displayed selective susceptibility to cutaneous HPVs, particularly β-HPV, but were otherwise healthy.

### The mutant αL proteins abolish LFA-1 (αLβ2) surface expression

**Fig. 2.**
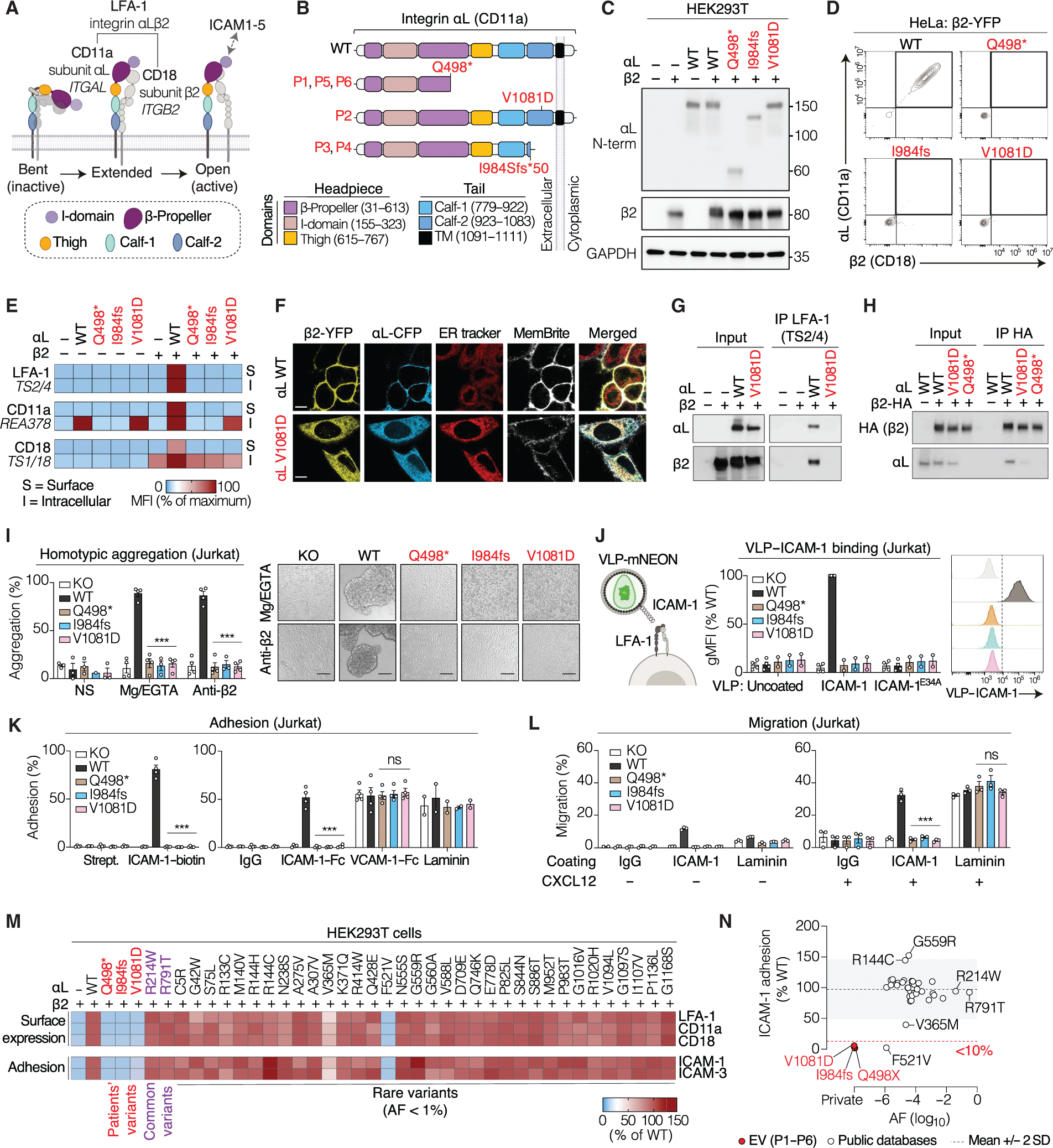
Integrin αL (CD11a) mutant alleles are LOF in vitro. (**A**) Schematic representation of the LFA-1 integrin heterodimer. (**B**) WT integrin αL and predicted impact of the patients’ variants. (**C**) Protein immunoblots of αL and β2 in HEK293T cells transfected with αL variants and β2. MWs (in kilodalton) are shown on the right. GAPDH, glyceraldehyde phosphate dehydrogenase. (**D**) Surface expression of LFA-1 subunits in HeLa cells transfected with αL variants and β2-YFP, assessed by flow cytometry. See also fig. S2E for quantification. (**E**) Surface (S) and intracellular (I) αL/β2 levels in HEK293T cells transfected with αL variants and β2 (mean; *N* = 3). See also fig. S2D. MFI, median fluorescence intensity. (**F**) Confocal images of HeLa cells transfected with αL-CFP (blue) and β2-YFP (yellow), with ER (red) and membrane (white) staining. Scale bars, 10 μm. (**G**) Protein immunoblots of αL and β2 after IP with an anti–LFA-1 (TS2/4) antibody in HEK293T cells transfected with αL and β2. (**H**) Protein immunoblots of αL and β2 after IP with an anti-HA antibody in HEK293T cells transfected with αL and β2-HA. (**I**) Homotypic aggregation of αL-KO Jurkat cells transduced with αL variants, induced by Mg^2+^/EGTA or activating anti-β2 Ab (CBR LFA1/2) (mean ± SEM; *N* = 4). Right: Representative microscopy images. Scale bars, 100 μm. NS, nonstimulated. (**J**) ICAM-1 binding to αL-KO Jurkat cells expressing αL variants using ICAM-1–pseudotyped VLPs containing mNEON (means ±SEM; *N* ≥ 2). (**K**) Adhesion of αL-KO Jurkat cells expressing αL variants to immobilized integrin ligands after Mg^2+^/EGTA stimulation (mean ± SEM; *N* = 4). (**L**) Migration of αL-KO Jurkat cells expressing αL variants through Transwell inserts coated with integrin ligands, with CXCL12 in the lower chamber (mean ± SEM; *N* = 3). (**M** and **N**) Expression and function of all homozygous αL variants found in public databases (see table S7) or in patients (red) in HEK293T cells transfected with αL and β2. (M) Heatmap showing LFA-1 expression and adhesion to ICAM-1 and ICAM-3, relative to WT. (N) Variants plotted by activity (ICAM-1 binding) versus AF. See also fig. S2H. Two-way ANOVA tests with Dunnett’s correction were used in (I), (K), and (L) (variants versus WT). ***P < 0.001; ns, not significant. Representative results from *N* ≥ 3 are shown in (C), (D), (F), (G), and (H).

*ITGAL* encodes the αL integrin chain (CD11a), which pairs with the β2 chain (CD18) to form the integrin lymphocyte function–associated antigen 1 (LFA-1) (Fig. 2A and table S6) (22, 23). LFA-1 is broadly and exclusively expressed on leukocytes (24). The Q498* and I984fs αL variants are truncating mutations predicted to remove the transmembrane and intracellular domains (Fig. 2B). The V1081D substitution in the Calf-2 domain is predicted to be structurally destabilizing [ΔΔ*G* (difference in the Gibbs free energy of folding between the mutant and wild-type proteins) = 3 kcal/mol (25)] (table S3). We studied the impact of the patients’ variants on the expression of αL and the αLβ2 (LFA-1) complex. Human embryonic kidney (HEK) 293T cells were transfected with plasmids encoding wild-type (WT) αL (NM_002209.3) or the patients’ variants, together with β2. Immunoblotting showed that WT and V1081D αL migrated at the expected size (~150 kDa), whereas Q498* and I984fs produced truncated proteins (Fig. 2C and fig. S2A). Flow cytometry of transfected HeLa (Fig. 2D) and HEK293T (fig. S2B) cells revealed complete loss of LFA-1 surface expression for all three variants compared with WT. We then tested the impact of the patients’ variants in a more relevant leukocyte cell line, the Jurkat T cell line. *ITGAL* knockout (KO) Jurkat cells (αL^KO^) were transduced with vectors expressing αL variants. Consistent with findings obtained in HEK293T and HeLa cells, the Q498*, I984fs, and V1081D variants abolished LFA-1 surface expression on T cells (fig. S2C).

### The mutant αL proteins and the β2 subunit are retained in the ER

The assembly of integrin α and β subunits into a heterodimer in the endoplasmic reticulum (ER) is a prerequisite for integrin surface expression (26). Consistently, WT αL and β2 reached the cell surface only if both subunits were expressed together in HEK293T (Fig. 2E and fig. S2D) or HeLa cells (fig. S2E). By contrast, the patients’ αL alleles prevented β2 from reaching the surface. The V1081D αL mutant was also retained in the cell, and αL Q498* and I984fs were undetectable by flow cytometry (Fig. 2E and fig. S2, D and E). The integrin α/β heterodimer is normally transported from the ER to the Golgi apparatus, where its glycosylation matures (27). Treatment with endoglycosidase H (Endo H), which cleaves only immature N-glycans, showed that only WT αL in the presence of β2 gave a high molecular weight (MW) band resistant to Endo H, indicating successful dimer formation and Golgi trafficking (fig. S2F). By contrast, the V1081D αL protein was fully sensitive to Endo H, suggesting that it failed to reach the Golgi compartment (fig. S2F). Consistent with these findings, confocal microscopy on HeLa cells demonstrated that the V1081D variant caused the retention of both αL and β2 in the ER (Fig. 2F). By contrast, WT αL was localized at the plasma membrane. Thus, the αL V1081D variant leads to a loss of surface expression because of impaired ER-to-Golgi trafficking.

### The V1081D αL protein does not bind β2

The interaction between the α-integrin Calf-2 domain and the β-integrin EGF4 and βTD domains stabilizes the dimer in its bent configuration (28). We hypothesized that the V1081D substitution in the αL Calf-2 domain might disrupt LFA-1 dimer formation. We tested this hypothesis by performing coimmunoprecipitation (co-IP) with lysates from HEK293T cells expressing either WT or V1081D αL together with β2. The TS2/4 monoclonal antibody (mAb), which recognizes αL only when associated with β2 (29), failed to immunoprecipitate the intracellular pool of αL V1081D (Fig. 2G), suggesting that V1081D alters its interaction with β2. A C-terminal hemagglutinin (HA) tag was added to β2 to ensure efficient β2 immunoprecipitation, regardless of potential alterations in mutant αLβ2 complex formation or structure. IP with an anti-HA mAb recovered similar amounts of β2 and indicated that V1081D strongly decreases the association between αL and β2 but does not completely abolish it (Fig. 2H). Collectively, these results indicated that the V1081D variant abolishes LFA-1 surface expression by disrupting αL association with β2. Residual dimers are detected in overexpression systems, but they remain trapped in the ER, possibly because of misfolding that prevents trafficking to the Golgi and cell surface.

### The mutant LFA-1 integrins are loss of function

LFA-1 promotes intercellular interactions by binding to members of the intercellular adhesion molecule (ICAM) family of transmembrane ligands on neighboring cells (30). We studied the impact of the patients’ *ITGAL* genotypes on LFA-1 function in αL^KO^ Jurkat T cells. Homotypic aggregation of Jurkat cells, which express ICAM-2 and ICAM-3 (fig. S2G), was induced by LFA-1 activation with Mg^2+^/EGTA (ethylene glycol tetraacetic acid) (31) or an activating mAb (CBR LFA1/2). As for αL^KO^ cells, no aggregate formation was observed with cells expressing αL Q498*, I984fs, or V1081D (Fig. 2I). In addition, these cells failed to capture viruslike particles (VLPs) displaying ICAM-1, indicating a complete loss of LFA-1–mediated ICAM-1 binding (Fig. 2J). Adhesion to immobilized ICAM-1 and ICAM-3 was also severely impaired, whereas adhesion to the β1-integrin ligands vascular cell adhesion molecule–1 (VCAM-1) and laminin was not affected by αL variants (Fig. 2K). We then assessed the integrin-dependent chemotactic motility of T cells. Jurkat cells expressing WT αL displayed robust migration toward CXCL12 in the presence of ICAM-1 (Fig. 2L). By contrast, cells expressing αL Q498*, I984fs, or V1081D displayed no LFA-1–dependent migration. As a control, β1-dependent migration, facilitated by laminin, was unaffected (Fig. 2L). Collectively, these results indicate that the patients’ αL alleles abolish LFA-1 function in an overexpression system.

### Integrin αL variants homozygous in the general population are neutral

The exact prevalence of EV is unknown, but with fewer than 350 kindreds reported to date (32, 33), it is probably <1 of 100,000 births. We investigated whether the cumulative frequency of biallelic *ITGAL* loss-of-function (LOF) genotypes in the general population was at least as rare as that of EV. We therefore evaluated the expression and function of all nonsynonymous *ITGAL* variants for which homozygotes were reported in public databases, encompassing a total of 1,224,276 individuals (table S7). This analysis included two common αL variants, R791T [allele frequency (AF), 31%] and R214W (AF, 4%), and 34 rare variants (AF, <1%). Of the 36 αL variants tested in HEK293T cells (cotransfected with β2), only the rare F521V variant severely impaired both LFA-1 surface expression and adhesion to ICAM-1 and ICAM-3 (Fig. 2M and fig. S2H). This allele (chr16:30496154-T-G) is extremely rare (AF, 1.2 × 10^−6^) and was identified in the homozygous state in only one individual. Another rare αL variant, V365M, was weakly hypomorphic (40% of WT activity), whereas the other 34 variants did not impair LFA-1 expression or function (Fig. 2M and fig. S2H). These results indicate that AR αL deficiency is rare in the general population (1 of 1,224,276) (Fig. 2N), consistent with αL deficiency being a genetic etiology of EV.

### Lack of LFA-1 expression by the patients’ cells

**Fig. 3.**
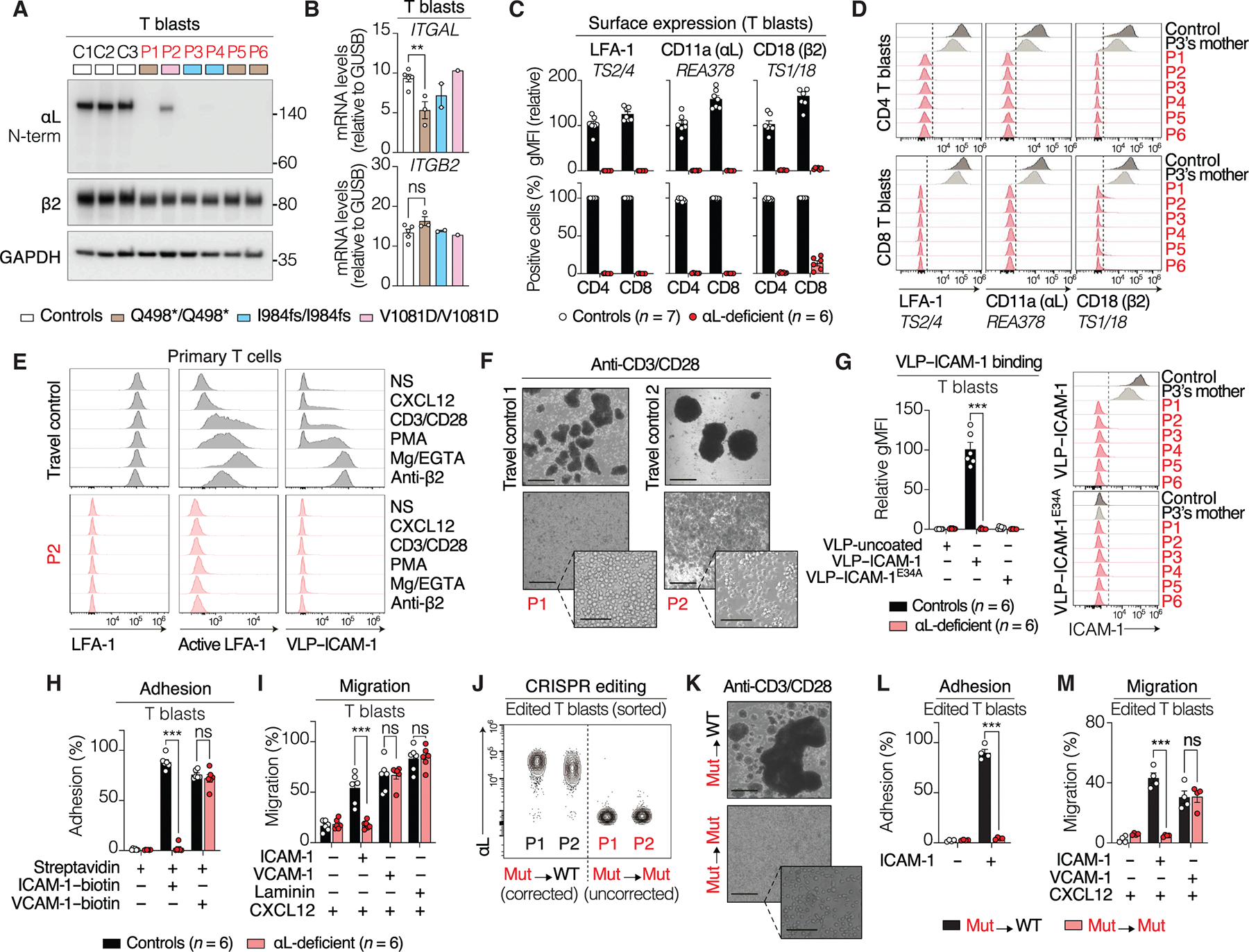
Complete LFA-1 deficiency in the patients’ primary cells. (**A**) Protein immunoblots of αL and β2 in T cell blasts of patients (*N* = 6) and controls (*N* = 3). MWs (in kilodalton) are shown on the right. (**B**) *ITGAL* and *ITGB2* mRNA levels in T cell blasts from patients (*N* = 6) and controls (*N* = 5) by quantitative reverse transcription PCR. (**C** and **D**) Surface expression of LFA-1 subunits on T cell blasts from patients (*N* = 6) and controls (*N* = 7) by flow cytometry. (C) Quantification. (D) Representative histograms. (**E**) LFA-1 surface expression, conformational activation (m24), and ligand binding (VLP–ICAM-1) in primary T cells after inside-out (CXCL12, anti-CD3/CD28, PMA) or pharmacological stimulation (Mg^2+^/EGTA, β2-activating antibody CBR LFA1/2). See also fig. S3D. (**F**) Homotypic aggregation of primary T cells after 48 hours of anti-CD3/CD28 stimulation. Representative images from P1, P2, and TC. Scale bars, 500 μm (low magnification) and 100 μm (high magnification). (**G**) VLP–ICAM-1 binding to patient (*N* = 6) and control (*N* = 6) T cell blasts after Mg^2+^/EGTA activation (mean ± SEM). (**H**) Adhesion of patient (*N* = 6) and control (*N* = 6) T cell blasts to immobilized integrin ligands after Mg^2+^/EGTA activation (mean ± SEM). (**I**) Migration of patient (*N* = 6) and control (*N* = 6) T cell blasts through Transwell inserts coated with integrin ligands, with CXCL12 in the lower chamber (mean ± SEM). (**J**) CRISPR-Cas9 correction of αL mutations in patient T cell blasts. See also fig. S3H. (**K**) Homotypic aggregation of CRISPR-edited T cell blasts after anti-CD3/CD28 stimulation. Scale bars, 500 μm (low magnification) and 100 μm (high magnification). (**L**) Adhesion of CRISPR-edited T cell blasts to ICAM-1 after Mg^2+^/EGTA activation (mean ± SEM). See also fig. S3I. (**M**) Migration of CRISPR-edited T cell blasts through Transwell inserts coated with integrin ligands, with CXCL12 in the lower chamber (*N* = 4; mean ± SEM). Student’s *t* tests (unpaired, two-tailed) were used in (A). Multiple *t* tests (unpaired, two-tailed) with Holm-Šidák correction were used in (G), (H), (I), (L), and (M). ***P < 0.001.

We evaluated LFA-1 expression in the patients’ leukocytes. Immunoblotting with an mAb recognizing the N terminus of αL revealed no detectable protein in T cells from P1, P3, P4, P5, and P6 (Fig. 3A), despite only weak nonsense-mediated mRNA decay (Fig. 3B), suggesting that the endogenous Q498* and I984fs proteins are unstable in primary cells. In T cells from P2 (V1081D), αL protein was detectable but migrated at a slightly lower MW than in controls (Fig. 3A), suggesting incomplete glycosylation. This was confirmed by the absence of an Endo H–resistant band in P2 (fig. S3A), indicating defective Golgi trafficking of αL. Total αL levels in P2 were also reduced (Fig. 3A), suggesting that heterodimerization with β2 contributes to αL stability. Accordingly, β2 KO in control T cells decreased αL abundance and prevented its glycosylation maturation (fig. S3B). Flow cytometry with mAbs targeting either αL, β2, or the αLβ2 heterodimer showed the complete absence of LFA-1 on the surface of the patients’ T cells (Fig. 3, C and D, and fig. S3C). Together, these findings indicate that the Q498* (P1, P5, and P6), I984fs (P3 and P4), and V1081D (P2) *ITGAL* genotypes abolish LFA-1 expression on the surface of the patients’ cells.

### Functional deficiency of LFA-1 in the patients’ cells

We then assessed LFA-1 expression, conformational activation, and function in primary T cells after integrin activation via inside-out signals [anti-CD3/CD28, CXCL12, and phorbol 12-myristate 13-acetate (PMA)] or direct pharmacological activation (Mg/EGTA, β2-activating antibody). Control T cells showed rapid LFA-1 activation upon stimulation, with minimal effects on surface expression levels, whereas patient T cells showed no detectable LFA-1 surface expression or activity across all conditions (Fig. 3E and fig. S3D). CD3/CD28 costimulation induced homotypic cell aggregation in T cells from healthy controls but not in those from patients (Fig. 3F). The patient’s T cells also failed to capture VLP–ICAM-1, indicating an impairment of ICAM-1 binding (Fig. 3G). Adhesion to ICAM-1– and ICAM-3–coated plates was also impaired in T cells from patients (Fig. 3H and fig. S3, E to G), whereas adhesion to VCAM-1 and laminin was similar for patient and control cells. In chemotaxis assays, control T cells displayed robust migration on ICAM-1, whereas no such migration was observed for T cells from patients (Fig. 3I). Last, CRISPR-Cas9–mediated correction of the genetic defects in T cells from P1 and P2 successfully restored LFA-1 expression to levels similar to those in control T cells (Fig. 3J and fig. S3H) and rescued LFA-1–dependent aggregation (Fig. 3K), adhesion (Fig. 3L and fig. S3I), and migration (Fig. 3M). Together, these results demonstrate that LFA-1 is completely deficient in the patients’ T cells and unresponsive to physiological (inside-out signals) and pharmacological activation.

### Somatic mosaicism in rare memory T cells

We assessed LFA-1 surface expression across patient leukocyte subsets. Consistent with our findings in expanded T cells, αL was undetectable on most peripheral blood mononuclear cells (PBMCs) from all patients (fig. S4A). However, rare αL^+^ cells were detected in P1 (2.2%), P2 (1.8%), P4 (0.8%), and P6 (2.8%) but not in the other patients. These αL^+^ cells were found exclusively among CD3^+^ memory T cells and belonged to γδ, CD8, or CD4 subsets, depending on the patient (fig. S4B). Sequencing of the *ITGAL* locus in αL^+^ cells revealed a spontaneous reversion of one allele to the WT form in P1 (fig. S4C) or correction of the frameshift by second-site compensatory insertions in P4 (fig. S4D). Because of the rarity of revertant cells and their failure to proliferate in response to CD3/CD28 or PHA (phytohemagglutinin) stimulation, it was not possible to investigate LFA-1 in these cells. Somatic reversion is more common in patients with IEI than previously thought (34, 35) and can underlie milder clinical presentations in some cases (36, 37). The clinical presentation of the six patients with αL deficiency was similar, and disease severity was not correlated with the presence, nature, or counts of these revertants, suggesting that these rare αL^+^ cells did not improve the clinical course of disease in these patients.

### LFA-1 is dispensable for human neutrophil migration

**Fig. 4.**
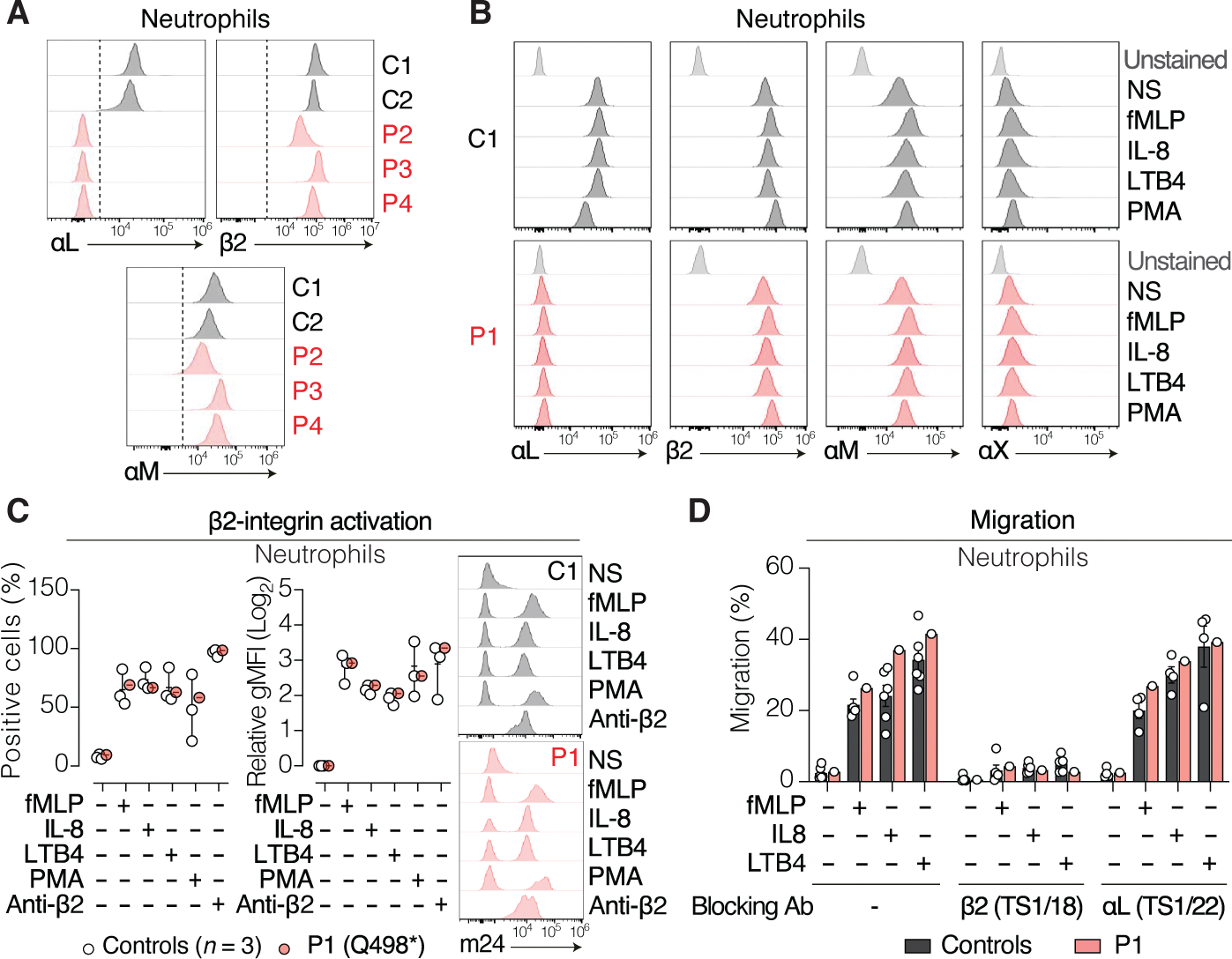
β2-integrin expression and function in patient neutrophils. (**A**) Surface expression of αL, β2, and αM on neutrophils from patients (P, *N* = 4) and controls (C, *N* = 2) assessed by flow cytometry. (**B**) Expression of β2-integrin subunits (αL, β2, αM, and αX) on resting and stimulated (fMLP, IL-8, LTB4, and PMA) neutrophils from patient (P1) and a control (C1). (**C**) β2-integrin activation in neutrophils from P1 and controls (*N* = 3) stimulated with the indicated agonists, assessed by flow cytometry using the activation-specific mAb (m24). anti-β2, activating anti-β2 mAb (CBR LFA1/2). (**D**) Migration of neutrophils from P1 and controls (*N* = 6; mean ± SEM) through Transwell inserts toward fMLP, IL-8, or LTB4 in the lower chamber, in the absence or presence of blocking antibodies against β2 (TS1/18) or αL (TS1/22). Dots represent individual samples.

β2 also pairs with αM (CD11b), αX (CD11c), or αD (CD11d) to form other β2 integrins expressed on myeloid cells, including neutrophils. Biallelic β2 mutations cause leukocyte adhesion deficiency type I (LAD-I) (38, 39), an IEI characterized by impaired neutrophil migration and life-threatening invasive bacterial and fungal infections in early childhood (40, 41). The absence of neutrophil-related phenotypes in patients with αL deficiency suggests that other β2 integrins might effectively compensate for the lack of LFA-1 in neutrophils. Consistently, surface expression of β2, αM, and αX on neutrophils of patients with αL deficiency was within the range of controls, both at baseline (Fig. 4A) and after stimulation with inflammatory [interleukin-8 (IL-8) and leukotriene B4 (LTB4)] and bacterial-derived [*N*-formyl-methionyl-leucyl-phenylalanine (fMLP)] chemoattractants (Fig. 4B). As expected, αL expression was completely absent under all conditions. In addition, αL-deficient neutrophils showed normal β2 conformational switching in response to neutrophil chemoattractants (Fig. 4C), indicating preserved inside-out signaling and activation of other β2 integrins in the absence of LFA-1. Last, αL-deficient neutrophils migrated normally toward fMLP, IL-8, and LTB4 in transwell assays (Fig. 4D). This migration was β2 integrin dependent, given that β2-blocking antibodies abolished neutrophil chemotaxis. By contrast, αL blockade had no effect on the migration of healthy donor neutrophils (Fig. 4D), confirming that LFA-1 is dispensable for human neutrophil migration. Together, these findings demonstrate that other β2 integrins are normally expressed, activated, and functionally competent in the absence of LFA-1, thereby supporting effective neutrophil migration and likely accounting for the lack of neutrophil-related phenotypes in patients with αL deficiency.

### Normal development of leukocyte subsets in the absence of LFA-1

**Fig. 5.**
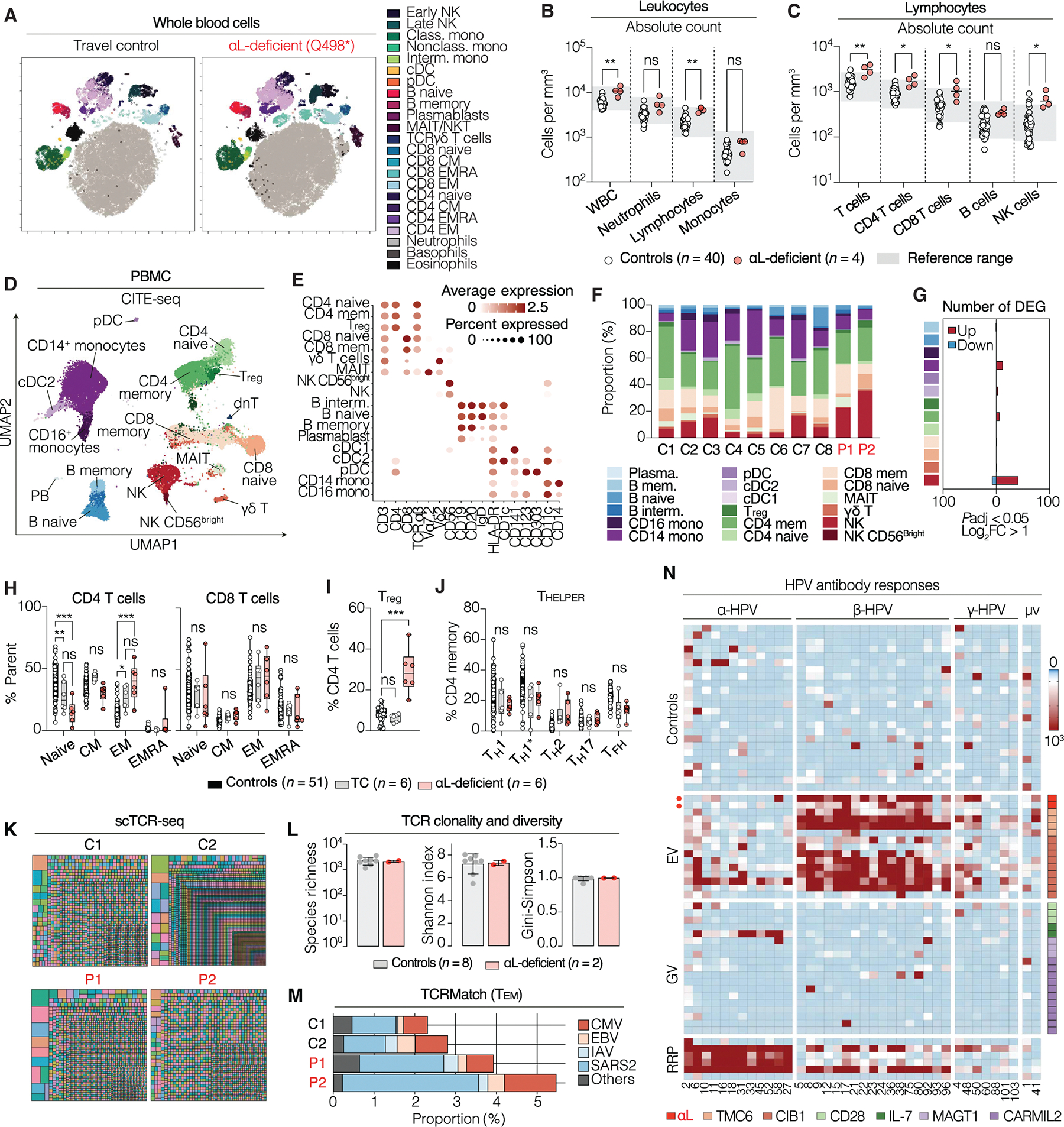
LFA-1 deficiency has a mild impact on leukocyte development and T cell differentiation. (**A**) Whole-blood CyTOF profiling of patients with αL deficiency (*N* = 4, aged 14 to 67 years) and controls (*N* = 51, aged 14 to 70 years). Representative *t*-distributed stochastic neighbor embedding (*t*-SNE) maps from P1 and their TC. Full datasets in data file S1. (**B** and **C**) Absolute counts of (B) leukocyte and (C) lymphocyte subsets from patients (*N* = 4) and controls (*N* = 40) based on complete blood count. See also table S8. WBC, white blood cells. (**D** to **G**) CITE-seq of PBMCs from patients (P1 and P2) and controls (*N* = 8). (D) UMAP clustering. PB, plasmablasts; dnT, double-negative T cells. (E) Representative protein markers. (F) Cell type proportions. (G) Pseudobulk differential expression; numbers of differentially expressed genes (DEGs) (adjusted *P* < 0.05) are shown per lineage. DEG lists are in data file S2. (**H** to **J**) T cell subset composition in patients (*N* = 6), TC (*N* = 6), and local controls (*N* = 51). See also fig. S5 [(I) and (J)]. T_FH_, T follicular helper. (**K** to **M**) scTCR-seq analysis of T cells from P1, P2, and controls (*N* = 8). (K) Tree map representations of TCR repertoires. (L) Clonality and diversity indices; see also fig. S5L. (M) Antigen specificity predictions for CD4^+^ T_EM_ clonotypes using TCRMatch. See also fig. S5M and data file S3. (**N**) HPV antibody responses in patients with EV (*N* = 15), generalized verrucosis (GV; *N* = 19), recurrent respiratory papillomatosis (RRP; *N* = 6), and controls (*N* = 24). Heatmap shows IgG responses to 38 HPV types by Luminex. Mann-Whitney [(B) and (C)], two-way ANOVA [(H) and (J)], or Kruskal-Wallis tests (I) were used for analysis. *P < 0.05, **P < 0.01, and ***P < 0.001. Box plots show the median and IQR (interquartile range); dots represent individual samples.

We analyzed blood from patients (*n* = 6, aged 14 to 78 years) and healthy controls (*n* = 51, aged 14 to 70 years) to study the impact of human LFA-1 deficiency on leukocyte development and homeostasis. Deep immunophenotyping by mass cytometry (cytometry by time-of-flight; CyTOF) indicated the presence and normal overall distribution of all myeloid and lymphoid subsets (Fig. 5A and data file S1). Blood cell counts revealed no major abnormalities except for a modest increase in total lymphocyte counts (Fig. 5, B and C; fig. S5, A to C; and table S8). The counts and distributions of myeloid populations were within normal ranges (Fig. 5B and fig. S5, D and E). Patients had a mild T cell lymphocytosis, affecting both CD4 and CD8 T cells (Fig. 5C). Natural killer (NK) cell numbers were also modestly increased (Fig. 5C), with a higher frequency of memory-like NKG2C^+^ NK cells (fig. S5F), consistent with the cytomegalovirus (CMV) seropositivity of the patients (table S5) (42, 43). Counts and frequencies were normal for unconventional T cell populations (fig. S5G) and B cell subsets (fig. S5H). In addition, single-cell transcriptomics and surface epitope detection [CITE-seq (cellular indexing of transcriptomes and epitopes)] analysis of PBMCs from patients (P1 and P2) and controls (*n* = 8) confirmed the presence and normal distribution of 18 leukocyte subsets in patient samples (Fig. 5, D to F). Pseudobulk differential expression analysis identified limited transcriptional changes across all leukocyte subsets (Fig. 5G and data file S2), suggesting normal transcriptional profiles. Human LFA-1 deficiency does not, therefore, affect the development of the major circulating leukocyte subsets. It has only a mild impact on leukocyte homeostasis, with a modest increase in T cell counts.

### LFA-1 deficiency does not affect T cell differentiation

The overall frequencies of T cell subsets were within the range of travel controls (TC, *n* = 6) or local controls (*n* = 51) (Fig. 5H), except for an increase in the count and frequency of memory regulatory T cells (T_reg_ cells) (Fig. 5I and fig. S5, I and J). All T helper subsets [T helper 1 (T_H_1), T_H_2, T_H_1*, T_H_17, and T follicular helper cells] were present at frequencies within the range of controls (Fig. 5J). We observed no abnormalities in the expression of anergy and exhaustion markers by memory T cells (fig. S5K). Single-cell T cell receptor sequencing (scTCR-seq) on T cells from P1 and P2 showed normal αβ repertoire clonality and diversity (Fig. 5K), with species richness, Shannon, and Gini-Simpson indices comparable to those of controls (*n* = 8) (Fig. 5L), including by iterative subsampling of memory subsets (fig. S5, L abd M). TCRmatch (44) analysis of purified CD4 effector memory (T_EM_) CDR3β sequences revealed broad antigenic specificity for common pathogens (data file S3), similar to that of controls. The expanded memory T cell clonotypes of P1 and P2 were predicted to be reactive to Epstein-Barr virus (EBV), CMV, influenza A, and severe acute respiratory syndrome coronavirus 2 (SARS-CoV-2), consistent with the patients’ documented history of prior infections (Fig. 5M). Collectively, these results indicated that the patients’ T cells developed and differentiated normally in vivo.

### LFA-1 deficiency does not affect humoral responses

Blood total B cell counts, subset frequencies, and plasmablast numbers were within the range of controls (fig. S5H and data file S1). The plasma concentrations of immunoglobulin classes were normal (table S9). Patients with LFA-1 deficiency had detectable antibodies against common pathogens in standard serological tests (fig. S1E and table S5). A multiplex serological analysis of anti-HPV humoral responses revealed that patients with αL deficiency displayed strong broad seropositivity for β-HPVs, like patients with isolated EV, due to mutations of *TMC6* or *CIB1* (Fig. 5N). By contrast, patients with generalized verrucosis caused by severe T cell defects (e.g., *IL7*, *CARMIL2*, or *CD28* deficiency) had few, if any, anti-HPV antibodies (Fig. 5N). The presence of anti-HPV immunoglobulin G (IgG) responses, which are T cell dependent, suggests that LFA-1 deficiency, like TMC6-TMC8-CIB1 deficiency, does not globally impair anti-HPV T and B cell immunity. Likewise, humoral responses to skin and urogenital commensal polyomaviruses were normal (fig. S5N). Collectively, these results indicate that patients with LFA-1 deficiency generate adequate T cell–dependent humoral responses against a diverse range of microbes, including HPV. Overall, inherited human LFA-1 deficiency has a limited impact on circulating leukocyte differentiation and function, possibly explaining the absence of infectious phenotypes other than EV.

### LFA-1–deficient T cells respond normally to polyclonal TCR stimulation

**Fig. 6.**
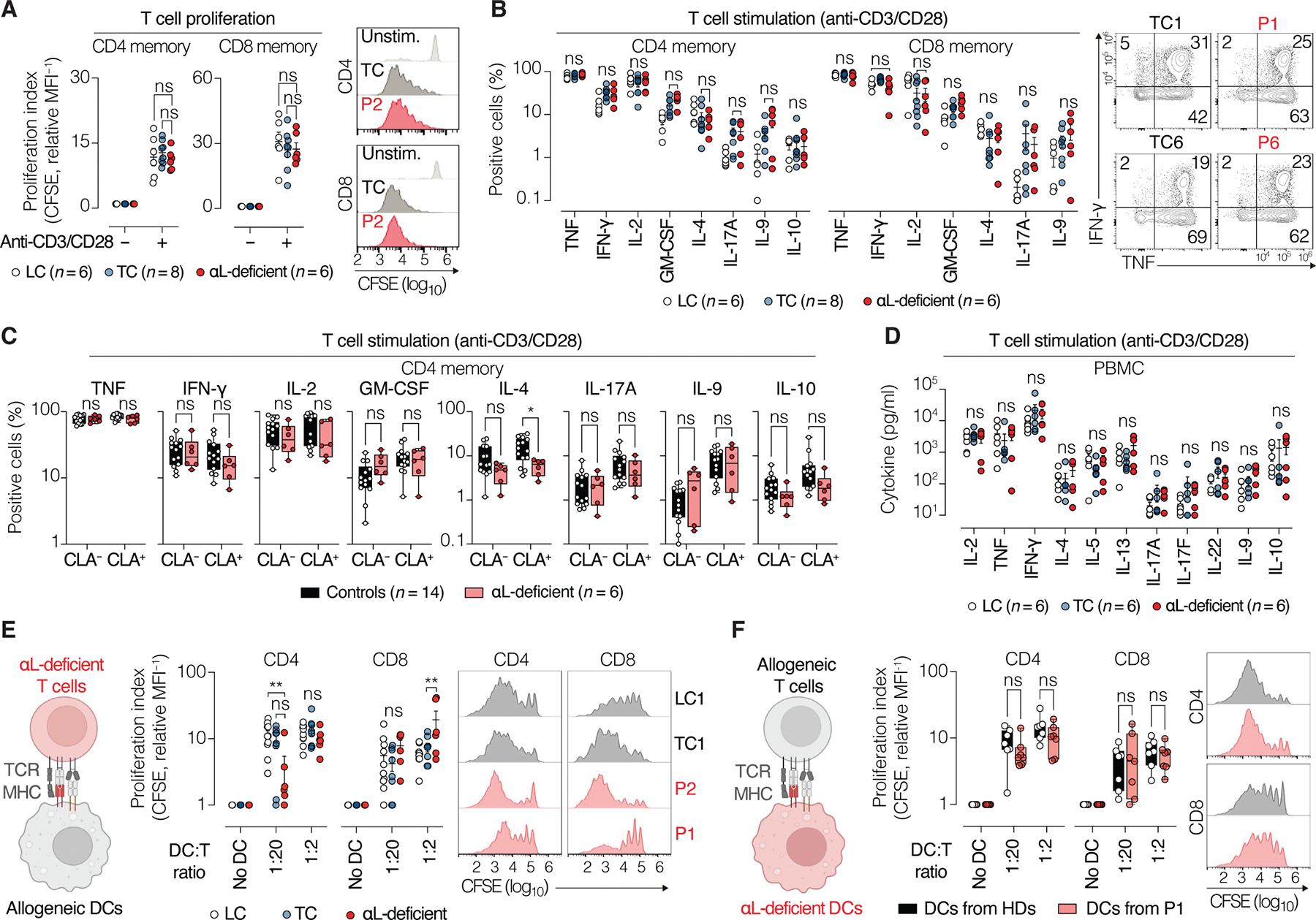
Limited impact of LFA-1 deficiency on polyclonal and APC-dependent activation of human T cells. (**A**) Proliferation of T cells from patients (*N* = 6), TC (*N* = 8), and local controls (LC; *N* = 6), assessed by CFSE dilution after 5 days of anti-CD3/CD28 stimulation. (**B**) Cytokine production by memory T cells from patients (*N* = 6), TC (*N* = 8), and LC (*N* = 6) after 24-hour anti-CD3/CD28 stimulation, assessed by flow cytometry. (**C**) Cytokine production by CLA^+^ (skin-tropic) and CLA^−^ memory CD4 T cells from patients (*N* = 6) and controls (*N* = 14). (**D**) Cytokines secreted by PBMCs from patients (*N* = 6), TC (*N* = 6), and LC (*N* = 6) after 24-hour anti-CD3/CD28 stimulation, measured by LEGENDplex. (**E**) Proliferation of αL-deficient T cells (from patients) cocultured with allogeneic MDDCs at the indicated ratios. Representative CFSE histograms are shown on the right (DC:T 1:2). Each dot represents one allogeneic pairing (LC, *N* = 10; TC, *N* = 8; P, *N* = 6). See also fig. S6 (A to D). (**F**) Proliferation of allogeneic T cells (from controls, *N* = 7) induced by MDDCs from P1 or healthy donors (HDs). Representative CFSE histograms are shown on the right (DC:T 1:2). Each dot represents one allogeneic pairing. See also fig. S6 (E and F). Two-way ANOVA tests with Tukey’s correction were used in (A), (B), (D), and (E). Mann-Whitney tests with Holm-Šidák correction were used in (C) and (F). *P < 0.05 and **P < 0.01. MHC, major histocompatibility complex.

We assessed the functions of CD4 and CD8 T cells from patients with αL deficiency (*n* = 6) in vitro, comparing their responses with those of TC (*n* = 8) and local controls (*n* = 6). Despite the lack of cluster formation (homotypic aggregation), the patients’ T cells displayed normal proliferation (Fig. 6A) and cytokine production (Fig. 6B) in response to ex vivo stimulation with anti-CD3/CD28 antibodies. Cytokine production by skin-tropic memory T cells, identified on the basis of their expression of the skin-homing marker cutaneous lymphocyte antigen (CLA) (45), was also similar in patients and controls (Fig. 6C). Moreover, the quantification of cytokines in the supernatants of PBMCs after anti-CD3/CD28 stimulation revealed similar levels of production for a broad panel of cytokines (Fig. 6D). The patients’ T cells therefore responded normally to polyclonal TCR stimulation in vitro, indicating intact signaling and preserved functional capacity.

### LFA-1 is dispensable for APC-dependent T cell activation

LFA-1 is a component of the immunological synapse between T cells and antigen-presenting cells (APCs) (46). However, the unexpectedly narrow phenotype of patients with LFA-1 deficiency—with normal control of systemic infections and normal memory T cell differentiation—suggests that LFA-1 may be dispensable for T cell priming in vivo. To test whether LFA-1 is required for APC-dependent T cell activation in vitro, we cocultured αL-deficient T cells from patients with allogeneic monocyte-derived dendritic cells (MDDCs). Both proliferation (Fig. 6E) and cytokine production (fig. S6A) of patients’ T cells were similar to those of TC. Likewise, αL KO in control T cells (fig. S6B) had minimal to no impact on activation by allogenic APCs (fig. S6, C and D). LFA-1–deficient T cells can therefore effectively engage with APCs and show normal APC-dependent activation. We then cocultured αL-deficient MDDCs from P1 with allogeneic control T cells. The absence of LFA-1 on MDDCs had only a minimal effect on T cell activation (Fig. 6F and fig. S6E). Moreover, coculture of αL-deficient MDDCs (P1) with αL-deficient T cells (P2) resulted in robust T cell proliferation (fig. S6F). Last, we assessed whether LFA-1 deficiency impaired the generation and/or recall of HPV-specific memory T cells in vivo. P1 was the only patient with a skin lesion positive for high-risk α-HPV18 and was evaluated for the presence of circulating HPV18-specific T cells. After PBMC stimulation with HPV18 E6 and E7 peptides, interferon-γ (IFN-γ)^+^ and tumor necrosis factor (TNF)^+^ memory T cells were detected in P1 but not in healthy donors (fig. S6G), consistent with the low prevalence of HPV18 responders in the general population (47). As a control, stimulation with the CEFSX peptide pool, which includes epitopes from a broad range of pathogens, elicited comparable responses in the patient and controls. These data demonstrate that HPV-specific T cells are present in the patient’s blood and are responsive to peptides presented by APCs in the absence of LFA-1. Collectively, these findings indicate that, in our assays, LFA-1 is dispensable on T cells and APCs for synapse-mediated activation.

### Expression of α4 and β2 integrins on human leukocytes

**Fig. 7.**
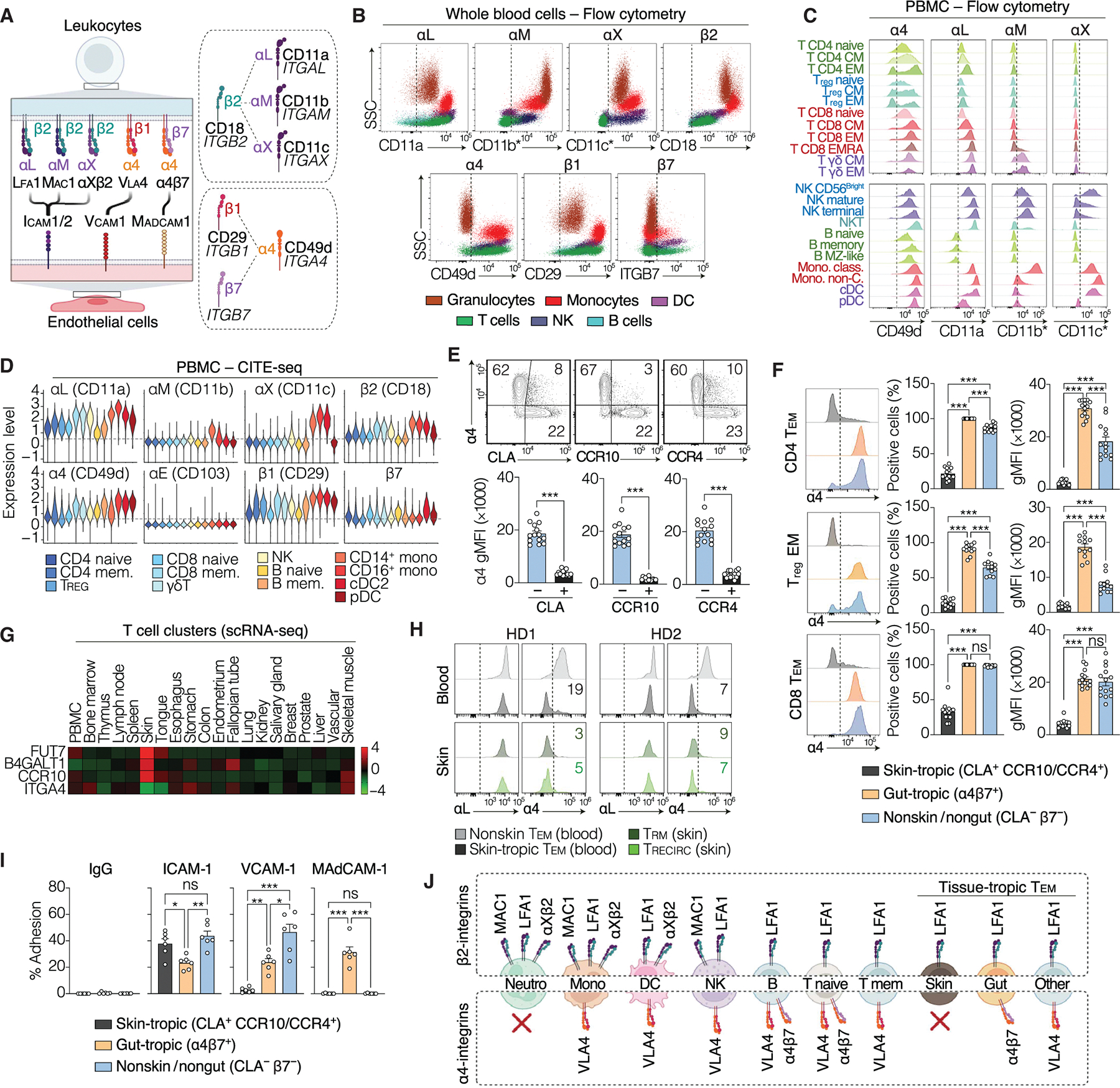
Expression of α4 and β2 integrins on human leukocyte subsets and tissue-specific T cells. (**A**) Leukocyte homing integrins and their endothelial ligands. (**B**) Integrin expression profile in whole blood from a healthy donor. (*) αM and αX were assessed postpermeabilization because of their large intracellular mobilizable pools. (**C**) Surface expression of integrin α subunits on PBMCs from a healthy donor; β subunits in fig. S7A. (**D**) CITE-seq analysis of integrin surface levels in leukocytes from healthy donors (*N* = 8). See also fig. S7B. (**E**) α4 expression relative to the skin-homing markers CLA, CCR10, and CCR4 on T_EM_ cells from healthy donors (*N* = 14). gMFI, geometric mean fluorescence intensity. (**F**) α4 expression in skin-tropic (CLA^+^CCR4/CCR10^+^), gut-tropic (CLA^−^β7^+^), and nonskin/nongut T_EM_ cells (CLA^−^β7^−^) from healthy donors (*N* = 14). (**G**) *Z*-score–normalized expression of α4 (*ITGA4*), *CCR10*, and CLA-generating enzymes *FUT7* and *B4GALT1* in T cell clusters from human tissues (source: proteinatlas.org). See also fig. S7G. (**H**) αL and α4 expression in skin-tropic and skin-resident T cells from healthy donors (HD; *N* = 2). T_RM_, CD69^+^CCR7^−^CD45RA^−^; T_RECIRC_, CD69^−^CCR7^−^CD45RA^−^. See also fig. S7 (H and I). (**I**) Adhesion of tissue-specific T_EM_ cells from healthy donor PBMCs to integrin ligands (mean ± SEM; *N* = 6). (**J**) Schematic summary of homing integrin expression across human leukocyte subsets. Unpaired *t* tests with Welch’s correction for unequal variances were used in (E); ordinary one-way ANOVA with Tukey’s correction in (F) (percentages) and (I); Brown-Forsythe ANOVA with Dunnett’s T3 correction in (F) (gMFI) and (I) (VCAM-1). *P < 0.05, **P < 0.01, and ***P < 0.001.

Leukocyte trafficking from blood to tissues depends on the interaction between leukocytic integrins and their endothelial ligands (48). Five human integrins support transendothelial migration: the α4 integrins VLA-4 (α4β1) and α4β7, which bind to VCAM-1 and mucosal addressin cell adhesion molecule–1 (MAdCAM-1), respectively, and the β2 integrins LFA-1 (αLβ2), Mac-1 (αMβ2), and αXβ2, which bind to endothelial ICAM-1 and ICAM-2 (Fig. 7A). Mac-1 and αXβ2 are predominantly found on myeloid subsets, whereas α4β7 is mostly expressed on gut-homing lymphocytes (49). By contrast, LFA-1 and VLA-4 are reported to be broadly expressed across leukocyte subsets (50, 51). We profiled leukocytic integrin expression with high granularity using flow cytometry on whole blood and CITE-seq on PBMCs from healthy individuals. Neutrophils expressed the three β2 integrins but were the only major leukocyte subset lacking α4 integrins (Fig. 7B). Most of the PBMC subsets coexpressed multiple homing integrins (Fig. 7, C and D, and fig. S7, A and B). Monocytes were positive for four homing integrins (VLA-4, LFA-1, Mac-1, and αXβ2), whereas NK cells, γδ T cells, and dendritic cells expressed three (VLA-4, LFA-1, and either Mac-1 or αXβ2). Naive αβ^+^ T cells had low levels of LFA-1, α4β7, and VLA-4. These integrins were up-regulated in memory αβ^+^ T cells, with moderate expression in central memory (T_CM_) T cells and high levels in T_EM_ cells. Unlike that of LFA-1, the expression of VLA-4 and α4β7 was heterogeneous in memory T cells, with a small population—particularly in the T_EM_ compartment—displaying a complete loss of α4-integrin expression (Fig. 7C). Thus, leukocytes coexpress at least one α4 integrin and one β2 integrin, with the exception of neutrophils and a small subpopulation of αβ^+^ T_EM_ cells lacking α4 integrins.

### Circulating skin-tropic T cells are α4 negative

We further characterized α4-negative T_EM_ cells by CITE-seq to determine whether they constituted a phenotypically distinct population in healthy individuals. Gene expression analysis revealed high levels of expression for skin-homing chemokine receptors (*CCR10*, *CCR4*, and *CCR8*) in α4-negative T_EM_ cells relative to α4^+^ T_EM_ cells (fig. S7C). Using flow cytometry, we confirmed that the loss of α4 integrins in T_EM_ cells was strongly correlated with acquisition of the skin-homing markers CLA, CCR10, and CCR4 (Fig. 7E). Integrin α4 was absent or weakly expressed in skin-tropic T_EM_ cells (CLA^+^ CCR4 or CCR10^+^), whereas gut-tropic (CLA^−^ β7^+^) and nonskin/nongut T_EM_ cells (CLA^−^ β7^−^) expressed high levels of α4 (Fig. 7F). By contrast, LFA-1 was expressed at similar levels in different tissue-specific T_EM_ cells (fig. S7D). In addition, α4 expression remained low to undetectable in skin-tropic T_EM_ cells cultured with homeostatic cytokines (IL-7, IL-2, and IL-15) or inflammatory stimuli (anti-CD3, TNF, and IL-4), whereas LFA-1 expression remained high and stable under all conditions (fig. S7, E and F). These observations suggest that the selective integrin expression patterns might be part of a fixed program intrinsic to tissue-specific memory T cells.

### Skin-resident and skin-tropic T cells rely on LFA-1 for tissue homing

In humans, memory T cells patrol or reside in nearly all tissues (52). To determine whether α4 loss was specific to skin T cells, we analyzed T cell clusters from various tissues using single-cell transcriptomic datasets (www.proteinatlas.org) (53). Cutaneous T cells had the lowest level of α4 (*ITGA4*) (Fig. 7G and fig. S7G) but the highest expression of skin-associated genes, such as *CCR10* and CLA-generating enzymes *FUT7* and *B4GALT1* (54). We then analyzed skin biopsy specimens from healthy donors. Consistent with previous observations (55), most cutaneous T cells expressed CLA and were either resident (T_RM_; CD69^+^ CD103^+/−^) (~80%) or recirculating (T_RECIRC_; CD69^−^) (~20%) memory T cells (fig. S7H). Both CLA^+^ T_RM_ and CLA^+^ T_RECIRC_ cells, as well as blood skin-tropic T_EM_ cells from the same donors, were α4 negative, whereas nonskin-tropic T_EM_ cells were α4^+^ (Fig. 7H and fig. S7I). We then assessed the differential adhesion capacity of tissue-specific circulating T_EM_ cells to various integrin ligands. Skin-tropic T cells adhered exclusively to the LFA-1 ligand ICAM-1, whereas nonskin-tropic T cells adhered to ICAM-1, VCAM-1, and, in the case of gut-tropic T cells, to MAdCAM-1 (Fig. 7I). Thus, skin-tropic and skin-resident T cells expressed LFA-1 as their sole homing integrin (Fig. 7J), suggesting that the skin homing of T cells may be exclusively dependent on LFA-1 in the absence of alternative homing integrins.

### Accumulation of skin-tropic T cells in the patients’ blood

**Fig. 8.**
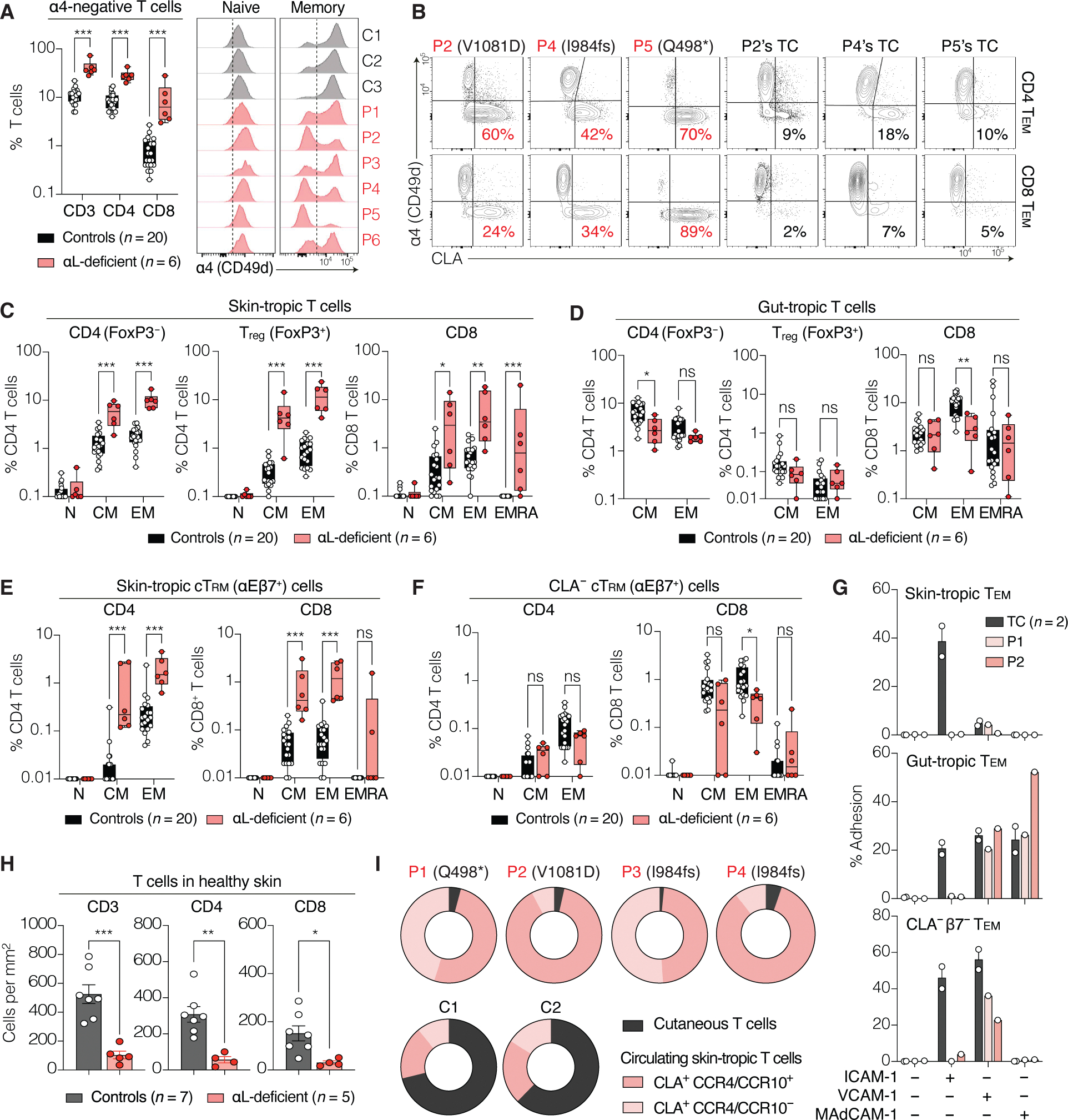
LFA-1 deficiency impairs the homing of T cells to the skin. (**A**) Frequencies of α4-negative T cells in PBMCs of patients with αL deficiency (*N* = 6) and healthy controls (*N* = 20), assessed by flow cytometry. See also fig. S8A. (**B**) α4 and CLA expression on CD4 and CD8 T_EM_ cells from patients (P; *N* = 3) and TC (*N* = 3). Representative dot plots are shown. (**C** and **D**) Frequencies of (C) skin-tropic (CLA^+^CCR4/CCR10^+^) and (D) gut-tropic (CLA^−^α4β7^+^) T cells in PBMCs from patients (*N* = 6) and controls (*N* = 20). (**E** and **F**) Frequencies of cT_RM_ cells (CD69^−^αEβ7^+^) in PBMCs from patients (*N* = 6) and controls (*N* = 20). (E) Skin-tropic cT_RM_ cells (CLA^+^CCR4/CCR10^+^). (F) CLA^−^cT_RM_ cells. See also fig. S8C. (**G**) Adhesion of tissue-specific T_EM_ cells from P1, P2, or TC (*N* = 2) to integrin ligands. (**H**) T cell counts in skin from patients with αL deficiency (*N* = 5) and controls (*N* = 7) (mean ± SEM). See also fig. S8 (D and E). (**I**) Blood-to-skin distribution of skin-specific T cells in patients (P; *N* = 4) and controls (C; *N* = 2). Mann-Whitney tests with Holm-Šidák correction were used in (A) and (C) to (F); unpaired *t* test was used in (H). *P < 0.05, **P < 0.01, and ***P < 0.001.

We hypothesized that the loss of LFA-1 might preferentially impair T cell homing to the skin. Flow cytometry analysis indicated a four-fold increase in the proportion of circulating α4-negative T cells in patients (*n* = 6) versus controls (*n* = 20) (Fig. 8A and fig. S8A), most of which expressed CLA (Fig. 8B). Patients with LFA-1 deficiency exhibited a marked increase in the proportion of skin-tropic (CLA^+^ CCR4/CCR10^+^) CD4 and CD8 T cells (Fig. 8C), affecting both T_CM_ and T_EM_ cells but not naïve cells. Total T_reg_ cell numbers were elevated in patients, but this increase was specifically due to the accumulation of skin-tropic memory T_reg_ cells (Fig. 8C and fig. S8B). By contrast, the proportion of gut-tropic (α4β7^+^) memory T cells was normal or decreased in patients (Fig. 8D). In humans, cutaneous CD103^+^ (αEβ7^+^) T_RM_ cells can down-regulate CD69 and reenter the bloodstream (56). We detected circulating αEβ7^+^ T_RM_ (cT_RM_) cells in healthy individuals, forming a rare population of total CD4 (~0.5%) and CD8 T cells (~2%) (fig. S8C). Among cT_RM_ cells, CLA and α4 delineated two populations: CLA^−^α4^+^ and CLA^+^α4^−^ cells. The CLA^+^α4^−^ population also expressed CCR4 and CCR10, further indicating skin specificity (fig. S8C). In the patients’ blood, CLA^+^ α4^−^ cT_RM_ proportions were markedly higher than those in controls (Fig. 8E), whereas CLA^−^α4^+^ cT_RM_ proportions were either similar or lower (Fig. 8F). Collectively, these findings highlight a selective accumulation of α4-negative skin-tropic T_EM_ and cT_RM_ cells in the patients’ blood, suggesting that skin-tropic T cells are sequestered in the circulation in the absence of LFA-1.

### Impaired homing of T cells to the skin in patients with LFA-1 deficiency

Skin-tropic memory T cells continuously recirculate between the skin and the blood (55–58). We hypothesized that their accumulation in the patients’ blood might result from impaired skin homing. We evaluated the capacity of T_EM_ cells from P1 and P2 to adhere to endothelial integrin ligands. CLA^−^ T_EM_ cells from patients with αL deficiency adhered to VCAM-1 and, in the case of gut-tropic cells, to MAdCAM-1 as well. By contrast, CLA^+^ T_EM_ cells from both patients failed to adhere to any of the ligands tested (Fig. 8G), indicating that LFA-1–deficient skin-tropic T cells are unable to engage with endothelial integrin ligands, thereby potentially impeding their extravasation into the skin. We tested this hypothesis by isolating T cells from the healthy skin of patients (*n* = 5) and controls (*n* = 7). After complete tissue digestion, skin specimens from healthy controls yielded five times as many T cells (mean: 524 cells/mm^2^) as healthy skin from patients (mean: 103 cells/mm^2^) (Fig. 8H). A similar deficit in T cell numbers was observed in EV lesions from P1 and P2 (fig. S8D). T cell subset characterization in P1’s skin indicated a marked decrease across all αβ^+^ subsets, affecting T_RM_ and T_RECIRC_ cells and both CD4 and CD8 lineages (fig. S8E). Conversely, γδ T cell counts were within the range of controls (fig. S8E). Using standard estimates of skin surface area and blood volume, we calculated the blood:skin ratio of skin-specific T cells. In healthy controls, this ratio was ~1:2, indicating that the estimated number of cutaneous T cells was twice the number of skin-tropic T cells in the bloodstream (Fig. 8I). By contrast, this ratio exceeded 20:1 in patients with αL deficiency, indicating that the vast majority of skin-specific T cells were found in the blood rather than the skin (Fig. 8I). Overall, these findings indicate that LFA-1 is essential for the skin homing of T cells and that its deficiency leads to the sequestration of skin-tropic T cells in the blood, thereby impairing immune surveillance of commensal HPVs in the skin.

## DISCUSSION

We report that AR complete and selective LFA-1 deficiency, caused by biallelic αL (CD11a) LOF mutations, is a genetic etiology of isolated EV. Genetic, biochemical, and immunological analyses established causality between αL deficiency and cutaneous HPV lesions. The mechanism of disease involves a selective impairment of T cell trafficking into the skin. LFA-1 deficiency disrupts the steady-state homing of skin-tropic memory T cells, a process ensuring continuous immunosurveillance of healthy skin. We show that this process is essential for controlling commensal and persistent cutaneous viruses, which are predominantly β-HPVs, and for preventing associated skin cancers. Low levels of cutaneous T cell infiltration are also observed in β2^−/−^ mice (59) and mice treated with anti–ICAM-1 mAbs (60), suggesting that LFA-1 may also be required for T cell entry into the skin in mice. LFA-1 is a component of the synapse between cytotoxic lymphocytes and their targets (22, 61). However, none of the patients had clinical disease related to herpesviral infections despite seropositivity to EBV and CMV, suggesting normal in vivo control by cytotoxic lymphocytes (62, 63). Consistently, αL^−/−^ mice efficiently control systemic viral infections, and the cytotoxic activity of their lymphocytes is preserved (64). Last, in our patients, LFA-1 deficiency has an unexpectedly limited impact on T cell priming and differentiation in vivo, and patient T cells show preserved APC-dependent activation in vitro. Overall, a selective defect of the skin-homing of T cells, rather than a profound defect of T cell priming or functions, likely underlies isolated EV in patients with αL deficiency.

The human skin harbors a large number of resident T_reg_ cells (65), which maintain peripheral immune tolerance and promote tissue repair (66). In patients with LFA-1 deficiency, skin-tropic T_reg_ cells are also sequestered in the blood. Their depletion in the skin might facilitate viral persistence. However, patients with *FOXP3* deficiency who lack T_reg_ cells do not develop EV lesions or other cutaneous warts (67), suggesting that skin T_reg_ cell deficiency alone cannot explain the expansion of cutaneous HPVs. In addition, none of the patients with LFA-1 deficiency reported delayed wound healing, skin fibrosis, or alopecia, and only one patient had a history of eczema. Although T_reg_ cells are reduced in the patients’ skin, they are not absent, and residual T_reg_ populations probably provided sufficient local regulation to maintain skin tolerance and repair. The small number of individuals with αL deficiency identified remains a limitation; studies of additional patients are needed to define the broader spectrum of cutaneous manifestations beyond EV.

Despite LFA-1 involvement in multiple intercellular adhesions, patients with αL deficiency have a narrow clinical phenotype, indicating extensive functional redundancy among leukocyte integrins. We found that most leukocytes express multiple homing integrins, typically including at least one from the α4 family and one from the β2 family. This pattern may provide versatility in trafficking while allowing for functional redundancy (49, 68–70). Neutrophils and skin-tropic memory T cells are the only exceptions, given that they lack α4 integrins and rely exclusively on β2 integrins for tissue homing. In patients with LAD-I, β2 deficiency disrupts all β2 integrins, thereby impairing neutrophil extravasation into inflamed tissues (71). By contrast, in patients with EV, αL deficiency selectively disrupts LFA-1 and impairs the homeostasis of skin-tropic T cells while preserving β2-dependent neutrophil migration (fig. S9). Consistently, patients with αL deficiency do not exhibit any clinical or biological features typical of LAD-I. Last, patients with LAD-I and complete β2 deficiency have not been reported to develop warts, probably because this condition is fatal in early infancy in the absence of hematopoietic stem cell transplantation (72), whereas HPV lesions in EV manifest later in childhood or adolescence. Consistently, disseminated warts have been reported in patients with partial β2 deficiency aged 10 to 38 years (73–75).

Modulating leukocyte trafficking has demonstrated clinical benefit in several inflammatory conditions and continues to hold promise for a broad range of diseases (76). Insights from IEI inform the rational design of drugs targeting homing receptors such as integrins by revealing the spectrum of essential functions and exposing critical vulnerabilities. Although integrin inhibition can attenuate pathological tissue inflammation, it may also impair local immune surveillance. This is illustrated by the development of HPV lesions in both patients with αL deficiency and individuals treated with anti-CD11a therapy (efalizumab) (77, 78), which was used to treat psoriasis between 2004 and 2009. Nevertheless, therapeutic antibodies targeting intact surface receptors might not perfectly phenocopy the corresponding genetic deficiency. Efalizumab ligation of LFA-1 exerts pharmacological effects beyond blocking ligand binding. It triggers inhibitory signaling that induces T cell hyporesponsiveness (79, 80) and dampens VLA-4 activity (81). This may explain the increased risk of John Cunningham (JC) virus encephalitis observed in patients treated with efalizumab (82) but not in patients with inherited αL or β2 deficiency.

Most infections are innocuous in most individuals, and most infectious diseases are confined to specific organs (83). Our study provides proof of concept that organ-selective infectious diseases can be due to a homing defect of tissue-specific memory T cells in patients whose immunity is otherwise intact. Tissue-specific memory T cells have a notable migratory selectivity and are subject to continuous trafficking to and in selective organs (49, 58). This compartmentalization of T cell responses has been widely documented (52, 84, 85), but its essential role in organ-specific protective immunity had not been definitively established in humans, in natural conditions of infection (86). Here, we demonstrate that a “lacunar” deficiency of tissue-homing T cells can mimic a deficiency of tissue-intrinsic immunity. Our findings, thus, shed light on an alternative pathomechanism for organ-selective infectious diseases, highlighting the fundamental role of T cell compartmentalization in organ-specific immunity.

## MATERIALS AND METHODS

### Patients and study participants

#### Study design

This study is an observational, family-based case series coupled to ex vivo mechanistic experiments. The objective was to characterize the genetic and mechanistic basis of EV in affected individuals by comparing patient samples with healthy controls using genetic analyses and immunological assays. The study investigated the functional and immunological consequences of biallelic *ITGAL* variants in patients with EV, with a focus on understanding susceptibility to skin commensal HPV. Because of the rarity of the condition, no a priori power calculation was feasible; we included all eligible patients identified through international collaboration. Inclusion criteria for patients were EV confirmed by clinical evaluation and supported by histological and virological findings (EV discovery cohort) and homozygous or compound heterozygous carriers of predicted deleterious variants in *ITGAL* (genotype-defined subgroup). Exclusion criteria for controls were known immunodeficiency, history of severe infectious disease, current systemic immunosuppression, or acute infection at the time of sampling. Randomization was not applicable, as group assignment was determined by disease status (EV) or genotype status (*ITGAL*). The primary end points were predefined as LFA-1 expression and function in relevant leukocyte subsets. Secondary end points included additional clinical, immunophenotypic, and functional readouts described in Results. Data collection stopped when all eligible individuals were enrolled and/or when available biospecimens were exhausted. Outliers were not removed. The study was not blinded. Experiments used independent biological samples (from patients and from healthy controls, as indicated); cell line–based experiments were repeated in independent experimental runs; the number of biological replicates and repeats for each assay is reported in the corresponding figure legends.

#### Human participants

Studies on data and samples from patients and healthy volunteers were approved by the institutional review and privacy boards, and the local ethics committees, in accordance with the Helsinki Declaration. Experiments were conducted in the US and France in accordance with local regulations and with the approval of the institutional review board (IRB) of the Rockefeller University (protocol no. JCA-0700) and INSERM (protocol no. C10-13). Written informed consent was obtained from all patients and family members in their countries of residence, in accordance with local regulations (P1 in the US, P2 in Algeria, and P3 to P6 in Iran) and with IRB approval. Healthy controls were recruited locally at the Rockefeller University in New York (US) (protocol no. JCA-0709) and the Imagine Institute in Paris (France), and travel controls were recruited in the countries of residence of the patients (US, Algeria, and Iran) in accordance with local regulations. Written informed consent was obtained from all healthy volunteers enrolled in this study.

#### Patients

Patient P1 (kindred 1) is a 69-year-old Iranian woman born to first-cousin parents, now residing in the US. She is homozygous for a truncating mutation (16:30519870-T-A) in *ITGAL*, leading to the replacement of the glutamine at position 498 with a premature stop codon (Q498*). She had disseminated and persistent flat warts from about 8 years of age onward. She also reported a history of common warts in childhood (not documented photographically) that did not persist into adulthood. Flat warts were treated by local cryotherapy, with limited therapeutic success. From her fifties onward, she developed multiple NMSCs, including a basal cell carcinoma (BCC) of the face and cutaneous squamous cell carcinomas (cSCCs) of the trunk and upper thighs, successfully treated by surgical excision. At the age of 62 years, she was diagnosed with invasive cSCC of the pubic area, positive for β-HPV5 and high-risk α-HPV18. P1 also developed a vulvar intraepithelial neoplasm positive for β-HPV8 and high-risk α-HPV66. She had not experienced other severe infectious diseases. P1 had no skin condition other than EV and never experienced delayed wound healing, skin fibrosis, alopecia, or inflammatory skin disease. Aside from EV, she only reported a single episode of onychomycosis affecting one nail at the age of 68 years.

Patient P2 (kindred 2) is a 33-year-old Algerian man with first-cousin parents, residing in Algeria. He is homozygous for a missense variant of *ITGAL* (16:30519870-T-A) replacing a valine at position 1081 with an aspartate (V1081D). He was vaccinated according to national vaccination schedules, including Bacille Calmette-Guérin (BCG) vaccination. No adverse events were noted. His symptoms first appeared at the age of 6 years, when his mother noticed the development of flat warts on his face and legs. Over time, the lesions progressively spread to other areas of the body. At the age of 12 years, P2 began a 4-year course of laser therapy and cryotherapy, resulting in minimal improvement. Treatment with acitretin for more than 2 years was ineffective and was discontinued due to drug-related liver toxicity. The patient has not experienced any other severe infections.

Patient P3 (kindred 3) is a 14-year-old Iranian boy, son of P4, born to second-degree cousin parents, and residing in Iran. He has had cutaneous warts since early childhood and is homozygous for a frameshift *ITGAL* mutation (16-30517055-GG-G) predicted to result in a truncated protein (I984Sfs*50). His clinical history includes episodes of extensive common warts on the hands and feet that resolved spontaneously. Dermatological examination showed persistent flat warts on the face and the back of the hands and common warts on the elbows. P3 received standard vaccinations, including BCG, without adverse events. He had no history of other infections. Patient P4 (kindred 3), a 56-year-old Iranian man and the father of P3, has also experienced cutaneous warts since childhood. He is homozygous for the I984Sfs*50 *ITGAL* mutation. Dermatological examination of P4 revealed flat warts on the back of the hands, common warts on the feet, and a lichenoid plaque on the right elbow. He had no history of other infections or notable medical conditions. Neither P3 nor P4 presented with genital warts.

Patient P5 (kindred 4) is a 35-year-old Iranian woman living in Iran, the daughter of P6. She has first-cousin parents. She is homozygous for the *ITGAL* truncating mutation Q498*. She reported a history of disseminated cutaneous warts since childhood. Dermatological examination revealed disseminated flat warts and mosaic warts on the plantar and interdigital areas of her feet. P5 also reported a history of eczema and skin allergies since childhood, which exacerbated at the age of 24 years. She has received antihistamine and unspecified anti-inflammatory drugs for her allergies, with limited improvement. She reported no history of other severe infections or extracutaneous conditions. Patient P6 (kindred 4) is a 78-year-old Iranian man, father of P5, residing in Iran and homozygous for the *ITGAL* Q498* mutation. He has had disseminated flat warts since childhood, with episodes of common warts on his feet. After the age of 60 years, he developed multiple BCCs on his scalp, which were surgically removed. These BCCs arose in areas previously exposed to radiation therapy for tinea capitis during childhood. Tinea capitis was a common condition in Iran for which radiation therapy remained the only available treatment until the introduction of griseofulvin in 1959. Like UV radiation, x-ray radiation acts as a skin cocarcinogen in patients with EV (87). It was not possible to test the BCCs for HPV.

### Cell culture and isolation

#### Cell lines

HEK293T cells (RRID:CVCL_0063) and HeLa cells (RRID:CVCL_0030) were purchased from the American Type Culture Collection (ATCC) and cultured in Dulbecco’s modified Eagle’s medium (DM-EM; Gibco, catalog no. A4192101) supplemented with 10% fetal bovine serum (FBS; Gibco) and 10 mM Hepes (Gibco, catalog no. 15630080). The Jurkat T cell line (clone E6-1, RRID:CVCL_0367) was purchased from ATCC and cultured in RPMI 1640 medium with GlutaMAX (Gibco, catalog no. 61870036), supplemented with 10% FBS. All cells were maintained at 37°C under an atmosphere containing 5% CO_2_ in a humidified incubator, and the medium was replaced every 2 to 4 days.

#### Peripheral blood mononuclear cells

PBMCs from patients (*n* = 6, protocol no. JCA-0700), travel controls (*n* = 8, protocol no. JCA-0709), and local healthy controls (*n* = 22, protocol no. JCA-0709) were isolated by Ficoll density gradient centrifugation (GE Healthcare) from venous blood samples collected into heparin-containing tubes. PBMCs were cryopreserved and stored in liquid nitrogen until use. Cryopreserved PBMCs were thawed and rested in RPMI 1640 supplemented with 10% FBS for 4 hours before use.

#### Primary T cell expansion and culture

T cell blasts were generated by expanding T cells from PBMCs in RPMI 1640 medium supplemented with 10% FBS, recombinant human IL-2 (Roche, catalog no. 11147528001) at a concentration of 10 ng/ml, and phytohemagglutinin-M (Thermo Fisher Scientific, catalog no. 10576015) at a concentration of 1% (v/v). The medium containing IL-2 was replaced every 48 to 72 hours, and expansion was continued until day 14 poststimulation, at which point cryopreserved T cell blast stocks were created. Cryopreserved T cell blasts were thawed in ImmunoCult XF T Cell Expansion Medium (STEMCELL Technologies, catalog no. 10981) supplemented with IL-2 (10 ng/ml), rested overnight, and stimulated with ImmunoCult Human CD3/CD28/CD2 T cell Activator (STEMCELL Technologies, catalog no. 10970). The medium containing IL-2 was replaced every 48 to 72 hours, and T cell blasts were restimulated with CD3/CD28/CD2 T cell Activator at a dilution of 1:40 to 1:100 every 21 days to maintain proliferation. T cell blasts were cultured for a minimum of 7 days poststimulation before use. CD4 and CD8 T cell blasts were isolated by fluorescence-activated cell sorting (FACS) with a BD FACS Aria cell sorter (BD Biosciences).

### Whole-exome sequencing and PCR sequencing

Genomic DNA was extracted from whole blood. Whole-exome sequencing (WES) was performed at the Yale Center for Genome Analysis (USA) and the Genomics Core Facility of the Imagine Institute (Paris, France). The DNA preparation was enriched in exons with the SureSelect XT Library prep Kit (Agilent Technologies, USA) and sequenced on an Illumina sequencer by paired-end sequencing (San Diego, USA). Raw reads were processed using the Illumina DRAGEN germline pipeline v4.0.3 for mapping to the hg38 reference genome and variant calling. All variants were annotated with Ensembl Variant Effect Predictor and our in-house annotation pipeline.

For the targeted sequencing of *ITGAL* variants, the relevant genomic regions were amplified by polymerase chain reaction (PCR), purified on a silica column (Macherey-Nagel, catalog no. 740609), and sequenced by the standard Sanger method or a long-read sequencing method. The Sanger sequencing reactions were performed with the Big Dye Terminator v1.1 kit (Thermo Fisher Scientific, catalog no. 4337451), purified by centrifugation through Sephadex G-50 Superfine resin (GE Healthcare, catalog no. GE17-0041-01), and subjected to capillary electrophoresis (Applied Biosystems 3730xL system, Thermo Fisher Scientific). Long-read PCR sequencing was performed by Plasmidsaurus (plasmidsaurus.com) with Oxford Nanopore Technology and custom analysis and annotation. SnapGene v7.2.1 (RRID:SCR_015052) was used for the alignment of PCR sequencing data. Primer sequences are provided in data file S4.

### Variant filtering and prioritization

We implemented a filtering and prioritization method to identify potential candidate disease-causing variants. Given that the family pedigrees suggested an AR mode of inheritance, we focused on biallelic variants in protein-coding genes on autosomes. We prioritized variants that were either rare (with an AF below 0.01 in gnomAD v4) or unreported in public databases (private variants). We enriched our list of candidates in potentially deleterious variants by selecting protein-truncating variants (pLOF)—such as stop-gain, essential splice-site, and frameshift variants—and in-frame insertion-deletion (indel) variants and missense variants with an AlphaMissense score above 0.56, the threshold for pathogenicity (88). We excluded variants with a combined annotation-dependent depletion (CADD) score below the 95% mutation significance cutoff (89) and those located in genes with a gene damage index (GDI) above 13, the threshold for AR inborn errors of immunity genes (90). The enrichment of the EV cohort (excluding those with known genetic etiologies) in rare biallelic predicted deleterious *ITGAL* variants relative to our in-house control cohorts (total of 25,329 individuals with other infectious diseases) was estimated by Firth penalized logistic regression adjusted for ethnicity (five principal components).

### Population genetics of human *ITGAL*

*ITGAL* has a GDI of 8.16, indicating a low accumulation of nonsynonymous variants in the general population (90). The consensus negative selection (CoNeS) score for *ITGAL* is low (−0.8), whereas the sHet score (selective effects of heterozygous protein truncating variants) is relatively high (0.027), indicating moderate negative selection. The probability of being LOF intolerant (pLI) for the *ITGAL* gene is 0.004, indicating that heterozygosity for truncating variants is tolerated in the general population. This suggests that *ITGAL* is not haploinsufficient, consistent with the absence of clinical disease in heterozygous (WT/M) relatives of the patients. The population genetics of *ITGAL* (table S6)—with its low mutational burden (GDI), no apparent haploinsufficiency (pLI), and moderate negative selection (CoNeS)—is consistent with an AR IEI underlying EV that only weakly impairs reproduction.

### Founder effect analysis and mutation dating

We investigated the possibility of a founder effect by analyzing WES data from members of kindred 1 (P1) and kindred 4 (P5 and P6), two apparently unrelated families carrying the *ITGAL* Q498* mutation. A shared haplotype spanning 3.8 Mb (chr16:28111905–31915298) was identified around the *ITGAL* variant, indicating a common ancestral origin. The age of the most recent common ancestor was inferred by the likelihood-based ESTIAGE (estimate the age of a mutation) method (91), which estimated the origin of the mutation at 55 generations ago (95% confidence interval: 17 to 224 generations). Assuming a mean generational interval of 27 years, the most recent common ancestor is estimated to have lived approximately 1500 years ago (95% confidence interval: 459 to 6,048 years).

### HPV detection and genotyping

Formalin-fixed paraffin-embedded skin biopsy sections from patients and controls were analyzed for HPV detection and genotyping at the French National Reference Center for Papillomaviruses (Besançon, France) in two complementary assays, as previously described (92, 93). The INNO-LiPA HPV Genotyping Extra II assay (Fujirebio, Ghent, Belgium) identifies 32 HPV genotypes (HPV-6, 11, 16, 18, 26, 31, 33, 35, 39, 40, 42, 43, 44, 45, 51, 52, 53, 54, 56, 58, 59, 61, 62, 66, 67, 68, 70, 73, 81, 82, 83, and 89), whereas a multiplex bead-based genotyping assay on a Luminex platform was used to detect 126 different HPV genotypes, including 22 αHPVs (HPV-6, 11, 16, 18, 26, 31, 33, 35, 39, 45, 51, 52, 53, 56, 58, 59, 66, 68, 70, 73, and 82), 46 βHPVs (HPV-5, 8, 9, 12, 14, 15, 17, 19, 20, 21, 22, 23, 24, 25, 36, 37, 38, 47, 49, 75, 76, 80, 92, 93, 96, 98, 99, 100, 104, 105, 107, 110, 111, 113, 115, 118, 120, 122, 124, 143, 145, 150, 151, 152, 159, and 174), 53 γHPVs (HPV-4, 48, 50, 60, 65, 88, 95, 101, 103, 108, 109, 112, 116, 119, 121, 123, 126, 127, 128, 129, 130, 131, 132, 133, 134, 148, 149, 156, SD2, 161, 162, 163, 164, 165, 166, 167, 168, 169, 170, 171, 172, 173, 175, 178, 179, 180, 184, 197, 199, 200, 201, and 202), and 8 HPVs associated with common warts (HPV-1, 2, 3, 4, 7, 10, 27, and 57).

### Luminex anti-HPV serological tests

Plasma samples were collected from patients with EV due to αL deficiency (P1 and P2), TMC6 deficiency (*n* = 4), or CIB1 deficiency (*n* = 9), from patients with generalized verrucosis (*n* = 19) or recurrent respiratory papillomatosis (*n* = 6), and from healthy controls (*n* = 24). Samples were shipped on dry ice to the German Cancer Research Center (DKFZ, Heidelberg, Germany) for serological analysis of HPV antibody responses using a Luminex-based multiplex assay, as previously described (94). This assay enables simultaneous detection of antibodies against the L1 antigens of HPV types 1, 2, 4, 5, 6, 8, 9, 10, 11, 12, 15, 16, 17, 18, 21, 22, 23, 24, 27b, 31, 33, 36, 38, 41, 45, 48, 50, 52, 58, 60, 75, 80, 88, 92, 93, 96, 101, and 103. For this assay, recombinant glutathione *S*-transferase fusion proteins of HPV L1 antigens were immobilized on polystyrene beads and incubated with serum samples at a 1:100 dilution. Bound immunocomplexes were detected using a biotinylated secondary antibody and streptavidin–phycoerythrin (PE) as a reporter. The resulting median fluorescence intensity values were used to generate the heatmap.

### Plasmids, cloning, and mutagenesis

Plasmids encoding human integrin αL (NM_002209.3) were constructed by inserting the full-length *ITGAL* coding sequence (derived from healthy donor cDNA) into pcDNA3.1(+) for transient transfection (pcDNA3-ITGAL) and pLZRS for stable transduction (pLZRS-ITGAL). The retroviral vector pLZRS-IRES-deltaNGFR (Addgene plasmid no. 72930) was a gift from E. van de Vosse. An integrin αL-mCFP (monomeric cyan fluorescent protein) fusion protein was created by subcloning the mCFP fragment from Addgene plasmid no. 8637 (a gift from T. Springer) into pcDNA3-ITGAL, fusing mCFP to the αL C terminus after removal of the stop codon (pcDNA3-ITGAL-mCFP).

Patient *ITGAL* mutations (Q498*, I984fs, and V1081D) were introduced into both pcDNA3-ITGAL and pLZRS-ITGAL constructs via site-directed mutagenesis with the KAPA HiFi HotStart Ready-Mix PCR Kit (Roche, via Thermo Fisher Scientific, catalog no. 50-196-5299). After amplification, PCR products were digested with DpnI (New England Biolabs, catalog no. R0176L) at 37°C for 1 hour for selective degradation of the parental plasmid. The resulting nicked plasmids were used to transform NEB 10-beta–competent *Escherichia coli* cells (New England Biolabs), and the amplified plasmids were purified with a plasmid midiprep kit (Zymo Research, catalog no. D4201).

Nonsynonymous *ITGAL* variants (*n* = 36) present in the homozygous state in public databases (gnomAD v4.1, UK Biobank, All of Us, BRAVO, GenomeAsia 100 K, Turkish Variome, Iranome, and Greater Middle East Variome) were introduced into pcDNA3-ITGAL by Q5 site-directed mutagenesis with the Q5 Hot Start High-Fidelity DNA Polymerase (New England Biolabs, catalog no. M0494L). The amplified products were treated with an enzyme mixture containing kinase, ligase, and DpnI (New England Biolabs, catalog no. M0554S) for 5 min at room temperature to facilitate plasmid circularization and to degrade the parental plasmid. The mutated plasmids were amplified in NEB 10–beta competent *E. coli* cells (New England Biolabs) and purified with a plasmid midiprep kit (Zymo Research, catalog no. D4201). A list of all the *ITGAL* variants is provided in table S5. Primer sequences are provided in data file S4.

The plasmids encoding human integrin β2 (ITGB2) used in this study were as follows: pcDNA3-ITGB2 (untagged), pCMV3-ITGB2-HA (C-terminal HA tag), and pEYFP-ITGB2-mYFP (C-terminal monomeric yellow fluorescent protein fusion). pcDNA3-ITGB2 (Addgene plasmid no. 8640; RRID: Addgene_8640) and pEYFP-ITGB2-mYFP (Addgene plasmid no. 8638; RRID:Addgene_8638) were gifts from T. Springer. pCMV3-ITGB2-HA was purchased from Sino biological (catalog no. HG10970-CY).

A plasmid encoding human ICAM-1 was constructed for pseudotyping VLPs by amplifying the ICAM-1 cDNA from Addgene plasmid no. 8632 (RRID: Addgene_8632; a gift from T. Springer) and inserting it into a pCMV6 backbone by InFusion cloning (Takara Bio, catalog no. 638946). The LFA-1–binding defective mutation (E34A) was introduced into the pCMV6-ICAM-1 vector by site-directed mutagenesis using 2xSuper PFX MasterMix (CoWin Biosciences, catalog no. CW2965S). All plasmid sequences were verified by whole-plasmid sequencing (PlasmidSaurus).

### Transient transfection

HEK293T cells (RRID:CVCL_0063) were transiently transfected with a plasmid encoding either WT or mutant αL (pcDNA3-ITGAL), together with a plasmid encoding β2 (pcDNA3-ITGB2), with the X-tremeGENE 9 DNA Transfection Reagent (Roche, catalog no. 6365787001). Briefly, HEK293T cells were used to seed six-well plates at a density of 1 × 10^6^ cells per well in 2 ml of DMEM supplemented with 10% FBS and 10 mM Hepes. The following day, a transfection mixture was prepared by combining 1 μg of pcDNA3-ITGAL, 1 μg of pcDNA3-ITGB2, and 6 μl of X-tremeGENE 9 in 300 μl of Opti-MEM Reduced Serum Medium (Thermo Fisher Scientific, catalog no. 31985070). The mixture was added dropwise to the cells. The medium was replaced 16 hours posttransfection. Cells were harvested for downstream experiments 60 hours posttransfection. Cells were detached by pipetting after incubation for 5 min in 0.2% bovine serum albumin (BSA) and 2 mM EDTA in phosphate-buffered saline (PBS).

HeLa (RRID:CVCL_0030) cells were transfected with a plasmid encoding either WT or mutant αL (pcDNA3-ITGAL), together with a plasmid encoding β2-mYFP (pEYFP-ITGB2-mYFP; Addgene plasmid no. 8638; RRID:Addgene_8638), with Lipofectamine 2000 (Thermo Fisher Scientific, catalog no. 11668019). Briefly, HeLa cells were used to seed 12-well plates at a density of 150,000 cells per well in 1 ml of DMEM supplemented with 10% FBS and 10 mM Hepes. The following day, a transfection mixture was prepared by combining 500 ng of pcDNA3-ITGAL, 500 ng of pEYFP-ITGB2-mYFP, and 3 μl of Lipofectamine 2000 in 200 μl of Opti-MEM Reduced Serum Medium (Thermo Fisher Scientific, catalog no. 31985070). The mixture was added dropwise to the cells. The medium was replaced 16 hours posttransfection. Cells were harvested for downstream experiments 48 hours posttransfection. Cells were detached by incubating with TrypLE Select (Thermo Fisher Scientific, catalog no. 12563011) for 5 min.

### Gene KO

CRISPR-mediated KO of integrins αL and β2 was achieved by delivering Cas9 ribonucleoproteins (RNPs) in cell lines or primary hu-man T cells. Pools of three single guide RNAs (sgRNAs) targeting either *ITGAL* (αL) or *ITGB2* (β2) and a scrambled negative control sgRNA were purchased from Synthego. The sequences of the sgRNAs were as follows: ITGAL (AUUUCUCACCAGGGUCAUCG, AAGCCUCUAUCAGUGCCAGU, and CCCCAGUUACUCACCUCUCA); ITGB2 (GUAAAGCGUCACUUUUUGUG, CUGGGUUUCAGCGAGGCUUG, and CCCCCUCCCCAGAACUUCAC); scrambled (GCACUACCAGAGCUAACUCA).

For gene KO in Jurkat cells, TrueCut Cas9 v2 (Thermo Fisher Scientific, catalog no. A36499) and the sgRNA pool were combined at an equimolar ratio (7.5 pmol each) in a final volume of 2 μl and incubated at room temperature for 10 min. Meanwhile, 200,000 cells were harvested, washed in PBS, and resuspended in 10 μl of resuspension buffer R. The resulting cell suspension (8 μl) was then mixed with 2 μl of RNP complexes and electroporated with the Neon NxT Electroporation System 10-μl Kit (Thermo Fisher Scientific, catalog no. N1096) with the following parameters: 1700 V, 20 ms, 1 pulse. The electroporated cells were transferred to 24-well plates containing 500 μl of prewarmed RPMI medium supplemented with 10% FBS. KO efficiency was assessed 4 days postelectroporation by flow cytometry, with an efficiency >90% for the αL sgRNAs. αL KO Jurkat cells were sorted by FACS and expanded to establish a stable cell line, which was subsequently transduced with patient αL variants.

For gene KO in T cell blasts, cells were restimulated 3 days before electroporation with ImmunoCult Human CD3/CD28/CD2 T cell Activator (STEMCELL Technologies, catalog no. 10970) and were cultured in ImmunoCult XF T Cell Expansion Medium (STEMCELL Technologies, catalog no. 10981) supplemented with IL-2 (10 ng/ml). Recombinant Cas9 (Thermo Fisher Scientific, catalog no. A36499) and the sgRNA pool were combined at an equimolar ratio (75 pmol each) in a final volume of 5 μl and incubated at room temperature for 10 min. Meanwhile, 2 × 10^6^ T cells were washed in PBS, resuspended in 100 μl of resuspension buffer R, and added to the 5 μl of RNP complexes. Cells were electroporated with the Neon NxT Electroporation System 100-μl Kit (Thermo Fisher Scientific, catalog no. MPK10096) with the following parameters: 1600 V, 10 ms, 3 pulses. After electroporation, cells were transferred to six-well plates containing 2 ml per well of prewarmed ImmunoCult medium supplemented with IL-2 (10 ng/ml). KO efficiency was assessed 6 days postelectroporation by flow cytometry, with an efficiency >90% for the αL sgRNAs and >80% for the β2 sgRNAs. αL and β2 KO T blasts were sorted by FACS and further expanded in vitro.

For gene KO in primary nonactivated T cells, T cells from healthy donors were obtained from freshly collected PBMCs. Recombinant Cas9 (Thermo Fisher Scientific, catalog no. A36499) and the sgRNA pool were combined at an equimolar ratio (75 pmol each) in a final volume of 10 μl and incubated at room temperature for 10 min. Meanwhile, 4 × 10^6^ T cells were washed in PBS, resuspended in 200 μl of resuspension buffer T, and added to the 10 μl of RNP complexes. Cells were electroporated with the Neon NxT Electroporation System 100-μl Kit (Thermo Fisher Scientific, catalog no. MPK10096) with the following parameters: 2200 V, 20 ms, 1 pulse. After electroporation, cells were transferred to 24-well plates containing 1 ml per well of prewarmed RPMI 1640 medium (2 × 10^6^ cells per well), supplemented with 10% FBS, IL-7 (2 ng/ml; R&D Systems, catalog no. BT-007-010), and IL-15 (2 ng/ml; Miltenyi Biotec, catalog no. 130095760). KO efficiency was assessed 7 days postelectroporation by flow cytometry, with an efficiency >80% for αL sgRNAs. αL KO primary T cells were used for allogenic reactions.

### Correction of the patient mutations by gene editing Design of sgRNA and the HDR donor template

CRISPR-Cas9–mediated gene editing was performed by homology-directed repair (HDR) with synthetic single-stranded DNA (ssDNA) donor templates for precise correction of the mutations in patient-derived primary cells. The sgRNAs and the ssDNA template were designed with the Alt-R CRISPR HDR Design Tool provided by Integrated DNA Technologies (IDT; www.idtdna.com). We generated homology arms of 90 nucleotides and introduced a silent mutation to prevent recutting after correction. A pool of three sgRNAs was selected, and both sgRNAs and ssDNA were selected to correspond to the same DNA strand. The design tool does not directly support the reversion of a mutation (Mut → WT). We therefore initially generated a design for the opposite change (WT → Mut) and then manually adjusted the design. Guide RNAs were purchased from Synthego, and the ssDNA repair templates (Alt-R HDR Donor Oligo) were purchased from IDT.

#### Assembly of the RNP complexes and the HDR donor template

We resuspended the sgRNAs in water at a final concentration of 30 μM (pool). RNP complexes were assembled by combining recombinant Cas9 (Thermo Fisher Scientific, catalog no. A36499) and sgRNAs at an equimolar ratio (75 pmol each), with 2.5 μl of Cas9 (5 μg/μl) and 2.5 μl of sgRNA (30 μM). The mixture was incubated at room temperature for 10 min, after which 150 pmol of template ssDNA (1.5 μl of a 100 μM solution) was added. For correction of the αL Q498* mutation, the following sgRNAs were used: GTTTATCTACTAGAGAAGAC, TTCTCTAGTAGATAAACACC, and TATCTACTAGAGAAGACAGG; with the following ssDNA template: GACGTGGACCAAGATGGGGAGACAGAGCTGCTGCTGATTGGTGCCCCACTGTTCTATGGGGAGCAGAGAGGAGGTCGGGTGTTTATCTACCAGCGGAGACAGGTGCGGCCAGGATCTGGAGCTGAGAGGGAGGAGGGAGAGCAGCAGAGATTCGCAGCTCCCAGTTATTCTGAAGGCTTTCTCTG. For correction of the αL V1081D mutation, the following sgRNAs were used: GGTTGTCATGAAGGATGACG, CTGCTCCCAGGTTGTCATGA, and CGTCATCCTTCATGACAACC; with the following ssDNA template: GGGAATGGAACCAGGTTCACAGGGACTTTCAGGGGTGGAAAAGGCATTTTCCGGCACTCCCCTCCCCCTGCTCTCAGGTTGTCATGAAAGTTGACGTCGTGTATGAGAAGCAGATGCTCTACCTCTACGTGCTGAGCGGCATCGGGGGGCTGCTGCTGCTGCTGCTCATTTTCATAGTGCTGT.

#### Electroporation of cells

Expanded T cells (T cell blasts) from P1 (αL Q498*) and P2 (αL V1081D) were restimulated 3 days before electroporation with ImmunoCult Human CD3/CD28/CD2 T cell Activator (STEMCELL Technologies, catalog no. 10970) and cultured in ImmunoCult XF T Cell Expansion Medium (STEMCELL Technologies, catalog no. 10981) supplemented with IL-2 (10 ng/ml). We collected 2 × 10^6^ cells by centrifugation, washed them once with PBS, and resuspended them in 100 μl of editing buffer. The cell suspension was then added to the RNP/ssDNA complex. Cells were electroporated with the Neon NxT Electroporation System 100-μl Kit (Thermo Fisher Scientific, catalog no. MPK10096) with the following parameters: 1600 V, 10 ms, 3 pulses. Following electroporation, cells were transferred to six-well plates containing 2 ml per well of prewarmed ImmunoCult medium supplemented with IL-2 (10 ng/ml). Editing efficiency (correction) was assessed 4 days postelectroporation by flow cytometry, with an efficiency of ~20% for the mutation present in P1 and ~30% for the mutation present in P2. Corrected (αL-positive) and uncorrected (αL-negative) cells were sorted by FACS and further expanded in vitro.

### Retrovirus production and cell transduction

HEK293T cells (RRID:CVCL_0063) were used to seed 10-cm dishes at a density of 5 × 10^6^ cells per dish in 10 ml of DMEM supplemented with 10% FBS and 10 mM Hepes. The following day, a transfection mixture was prepared by combining 5 μg of pLZRS-IRES-deltaN-GFR(CD271) encoding either WT αL or patient variants, or an empty vector (Addgene plasmid no. 72930), 2.5 μg of a Gag/Pol plasmid, 2.5 μg of an Env (VSV-G) plasmid, and 30 μl of X-tremeGENE 9 (Roche, catalog no. 6365787001) in 1 ml of Opti-MEM (Thermo Fisher Scientific, catalog no. 31985070). The mixture was incubated for 15 min at room temperature and then added dropwise to the cells. The medium was replaced 16 hours posttransfection, and the supernatants were collected 48 hours posttransfection. The supernatants were passed through filters with 0.45-μm pores and concentrated 100-fold with a Retro-X concentrator (Takara Bio, catalog no. 631456), according to the manufacturer’s instructions. Integrin αL KO Jurkat cells were transduced with the concentrated retrovirus at a 1:100 dilution. Stably transduced cells were isolated with anti-CD271 antibody-coated beads (Miltenyi Biotec, catalog no. 130-099-023).

### Extraction of mRNA, cDNA synthesis, and quantitative reverse transcription PCR

T cell blasts were harvested and washed with ice-cold PBS. Total RNA was extracted with the Quick-RNA 96 Kit (Zymo Research, catalog no. R1052), according to the manufacturer’s instructions. RNA samples were reverse-transcribed to generate cDNA with the SuperScript IV VILO Master Mix (Thermo Fisher Scientific, catalog no. 11756050), according to the manufacturer’s instructions. Each reaction was set up with 200 ng of RNA in a final volume of 5 μl. The resulting cDNA was diluted to a final volume of 50 μl before use. Quantitative real-time PCR was performed on an Applied Biosystems 7500 Fast Real-Time PCR System with the TaqMan Universal PCR Master Mix (Thermo Fisher Scientific, catalog no. 4304437), according to the manufacturer’s instructions, with 10 ng of cDNA in each reaction. The relative expression of *ITGAL* (Thermo Fisher Scientific, catalog no. Hs00158218_m1) and *ITGB2* (Thermo Fisher Scientific, catalog no. Hs00164957_m1) was determined as 2^−ΔCt^ values after normalization against *GUSB* (Thermo Fisher Scientific, catalog no. Hs00939627_m1) mRNA levels.

### Protein immunoblots

Cells were harvested and washed with ice-cold PBS. Total protein extracts were prepared by lysing cell pellets in NP-40 lysis buffer [150 mM NaCl, 50 mM tris (pH 8.0), and 1.0% NP-40] supplemented with cOmplete Mini Protease Inhibitor Cocktail (Roche, catalog no. 11836170001) immediately before use. Lysates were incubated for 30 min on ice and then clarified by centrifugation at 10,000*g* for 10 min at 4°C. Protein yield was determined with the Pierce BCA Protein Assay (Thermo Fisher Scientific, catalog no. 23225), and samples containing equal amounts of protein (~10 μg) were separated by SDS–polyacrylamide gel electrophoresis (SDS-PAGE; Mini-PROTEAN TGX Gels 4 to 20%, Bio-Rad, catalog no. 4561096). Proteins were transferred onto a polyvinylidene difluoride membrane in a wet transfer system (SureLock Midi, Thermo Fisher Scientific, catalog no. STM2001) at 20 V for 45 min at 4°C. The membrane was blocked by incubation in 5% milk in PBST (0.05% Tween 20 in PBS) for 1 hour at room temperature. The membranes were then probed with the desired primary antibody (overnight incubation at 4°C or incubation for 2 hours at room temperature) followed by the appropriate secondary antibody conjugated to horseradish peroxidase (HRP; 1 hour at room temperature). Primary antibodies against the following targets were used: integrin αL (N-terminal) (clone 27; Santa Cruz Biotechnology; catalog no. sc-135951; 1:1000 dilution in PBST), integrin αL (C-terminal) (clone E5S9K; Cell Signaling Technology; catalog no. 26703S; 1:1000 dilution in PBST), integrin β2 (clone D4N5Z; Cell Signaling Technology; catalog no. 73663S; 1:1000 dilution in PBST), glyceraldehyde phosphate dehydrogenase (HRP-conjugated) (clone 1E6D9; Proteintech; catalog no. HRP-60004; 1:10,000 dilution in PBST 5% milk), and HA-Tag (HRP-conjugated) (clone 6E2; Cell Signaling Technology; catalog no. 2999S; 1:1000 dilution in PBST). Antibody binding was detected with the Pierce HRP substrate for enhanced chemiluminescence (Thermo Fisher Scientific, catalog no. 32106). Images were acquired on an Amersham Imager 600 (GE Life Sciences). Uncropped immunoblots are provided in data file S5.

### Profiling of LFA-1 glycosylation and trafficking with PNGase F and Endo H

The treatment of total protein extracts with peptide *N*-glycosidase (PNGase) F removes all N-linked oligosaccharides, reducing the size of N-glycosylated proteins to that of their unglycosylated forms. By contrast, Endo H selectively cleaves high-mannose glycans generated in the ER but cannot remove the complex glycans formed in the Golgi compartment. Glycoproteins that have entered the Golgi compartment therefore become resistant to Endo H digestion while remaining sensitive to PNGase F. The unique specificity of Endo H and PNGase F was used to monitor the intracellular trafficking of αL and β2. Total cell extracts were prepared by lysing cell pellets in NP-40 lysis buffer [150 mM NaCl, 50 mM tris (pH 8.0), and 1.0% NP-40]. Protein extracts were denatured by combining 30 μg of protein, 3 μl of 10× glycoprotein denaturing buffer (New England Biolabs), and water in a total volume of 30 μl. This mixture was incubated at 99°C for 10 min and then chilled on ice. For PNGase F digestion, 10 μl of the denatured protein extract was combined with 2 μl of 10× GlycoBuffer 2, 2 μl of 10% NP-40, 1 μl of PNGase F (New England Biolabs, catalog no. P0704S), and 5 μl of water in a 20-μl reaction, which was incubated at 37°C for 1 hour. For Endo H digestion, 10 μl of denatured protein extract was mixed with 2 μl of 10× GlycoBuffer 3, 2 μl of Endo H (New England Biolabs, catalog no. P0702S), and 6 μl of water in a 20-μl reaction, which was incubated at 37°C for 1 hour. For mock digestion, 10 μl of protein extract was mixed with 2 μl of 10X GlycoBuffer 3 and 8 μl of water in a 20-μl reaction, which was incubated at 37°C for 1 hour. A volume of 10 μl from each reaction (~5 μg protein) was subjected to SDS-PAGE and protein immunoblot analysis. Uncropped immunoblots are provided in data file S5.

### Coimmunoprecipitation

HEK293T cells transfected with a plasmid encoding either WT or mutant αL and with a plasmid encoding β2-HA (C-terminal HA tag) were mechanically harvested 60 hours posttransfection and washed twice with ice-cold PBS. Total protein extracts were prepared by lysing cell pellets in IP buffer [100 mM NaCl, 50 mM tris (pH 7.4), 1.0% Triton X-100, 10% glycerol, and 0.5 mM EDTA] supplemented with cOmplete Mini Protease Inhibitor Cocktail (Roche, catalog no. 11836170001). Lysates were incubated on a rotating platform for 2 hours at 4°C and then clarified by centrifugation at 10,000*g* for 10 min at 4°C. Whole-cell lysates (~500 μg of protein) were incubated with 10-μl anti-HA magnetic beads (Thermo Fisher Scientific, catalog no. 88836) or with 2 μg anti-αL antibody (clone TS2/4) combined with Dynabeads Protein G (Thermo Fisher Scientific, catalog no. 10003D) in a total volume of 1 ml of IP buffer. Samples were rotated for 4 hours at 4°C and then washed five times with 1 ml of IP buffer on a DynaMag-2 magnetic rack (Thermo Fisher Scientific). Protein complexes were eluted with 0.1 M glycine (pH 3) for 10 min at room temperature and neutralized with 10 μl of tris (pH 8), and 10 μl of each co-IP sample was subjected to SDS-PAGE and analyzed by protein immunoblotting. Uncropped immunoblots are provided in data file S5.

### Analysis of the expression of LFA-1 and ICAMs by flow cytometry

Cells were harvested and washed once in PBS supplemented with 0.2% BSA and 2 mM EDTA (FACS buffer). Nonspecific binding was minimized by first incubating the cells with Human TruStain FcX (BioLegend, RRID:AB_2818986). For surface staining, cells were incubated with one or more of the following antibodies diluted in FACS buffer: anti–integrin αL (CD11a)-PE (dilution: 1:1000), clone TS2/4 (BioLegend, catalog no. 350606/RRID:AB_10660753); anti–integrin αL (CD11a)-VioBlue (dilution: 1:200), clone REA378 (Miltenyi Biotec, catalog no. 130-105-477/RRID:AB_2654622); anti–integrin β2 (CD18)-Allophycocyanin (dilution: 1:1000), clone TS1/18 (BioLegend, catalog no. 302114/RRID:AB_2280568); anti–integrin β2 (CD18)-PE-Vio770 (dilution: 1:1000), clone REA1112 (Miltenyi Biotec, catalog no. 130-119-265/RRID:AB_2751670); anti–ICAM-1 (CD54)-PE-Cy7 (Cyanine 7) (dilution: 1:500), clone HA58 (BioLegend, catalog no. 353115/RRID:AB_2715943); anti–ICAM-2 (CD102)-FITC (dilution: 1:500), clone CBR-IC2/2 (BioLegend, catalog no. 328507/RRID:AB_1134236); or anti–ICAM-3 (CD50)-PE (dilution: 1:500), clone MEM-171 (Thermo Fisher Scientific, catalog no. MA1-10244/RRID:AB_11152541). After incubation for 1 hour at 4°C, cells were washed and resuspended in FACS buffer for immediate acquisition. For intracellular staining, cells were fixed and permeabilized with the Foxp3 Transcription Factor Staining Buffer Set (eBioscience kit; Thermo Fisher Scientific, catalog no. 00-5523-00) according to the manufacturer’s instructions. Permeabilized cells were incubated for 1 hour at 4°C with antibodies diluted in the 1× permeabilization buffer provided with the kit. Flow cytometry was performed on an Attune NxT Flow Cytometer (Thermo Fisher Scientific, RRID:SCR_019590). Data were analyzed with FlowJo (v10.10.0) (RRID:SCR_008520).

### Confocal imaging

HeLa cells were transfected with a plasmid encoding either WT αL (pcDNA3-ITGAL-mCFP) or V1081D αL (pcDNA3-ITGAL-V1081D-mCFP) fused to mCFP, along with a plasmid encoding β2-mYFP (pEYFP-ITGB2-mYFP; RRID:Addgene_8638; a gift from T. Springer). After 24 hours, cells double-positive for mYFP and mCFP were isolated by FACS and plated in eight-well μ-Slides (ibidi, catalog no. 80826) at a density of 50,000 cells per well. The next day, cells were stained with ER-Tracker Red (BODIPY TR Glibenclamide) (Thermo Fisher Scientific, catalog no. E34250) and MemBrit Fix-ST 755/777 (Thermo Fisher Scientific, catalog no. 50-196-4524), according to the manufacturer’s instructions. Cells were then imaged with an inverted LSM 980 laser scanning confocal microscope (Zeiss) equipped with a 60× 1.4 numerical aperture oil-immersion objective lens, 34 spectral detection channels, and an Axiocan705 monochrome camera, with Zeiss ZEN Blue acquisition software (v.3.5).

### VLP–ICAM-1 production

HEK293T cells were used to seed 10-cm dishes at a density of 5 × 10^6^ cells per dish. The next day, cells were transfected with 8 μg of psPAX2-D64V-mNeon (Addgene plasmid no. 196509; RRID:-Addgene_196509, a gift from H. Chang) and 4 μg of pCMV6-ICAM-1 in the presence of X-tremeGENE 9 (Roche, catalog no. 6365787001) transfection reagent. The medium was replaced the following day, and supernatants were collected 2 days posttransfection, passed through 0.45-μm filters, and concentrated 100 times with a Lenti-X concentrator (Takara Bio, catalog no. 631231) according to the manufacturer’s instructions.

### ICAM-1 binding assays

ICAM-1 binding to Jurkat cells or T cell blasts was assessed with VLPs containing mNEON and displaying either WT ICAM-1, a binding-deficient ICAM-1 mutant (E34A), or uncoated VLPs as a control. LFA-1 activation was induced by Mg/EGTA treatment. Integrin αL KO Jurkat cells transduced with WT αL, patient variants, or an empty vector were resuspended at a density of 2 × 10^6^ cells/ml in RPMI medium containing 0.1% BSA, supplemented with 5 mM MgCl_2_ and 2 mM EGTA (Mg/EGTA). T cell blasts from patients and controls were prepared under the same conditions. In total, 50,000 cells (25 μl) per well were used to seed 96-well V-bottom polypropylene plates (Greiner Bio-One, catalog no. 651201). We then added 2.5 μl of concentrated VLP–ICAM-1 per well (see VLP–ICAM-1 production). Plates were incubated at 37°C for 30 min to allow binding. The cells were then washed twice with PBS containing calcium and magnesium (PBS^++^) supplemented with 0.1% BSA. ICAM-1 binding was quantified by flow cytometry based on mNEON fluorescence intensity, with an Attune NxT Flow Cytometer (Thermo Fisher Scientific, RRID:SCR_019590). Data were analyzed with FlowJo (v10.10.0, RRID:SCR_008520).

### T cell homotypic aggregation assays

LFA-1–dependent homotypic aggregation was assessed in αL KO Jurkat cells transduced with WT αL, patient variants, or an empty vector. Cells were resuspended at a density of 2 × 10^6^ cells/ml in RPMI medium containing 0.1% BSA, supplemented with either 5 mM MgCl_2_ and 2 mM EGTA (Mg/EGTA) or an activating anti-β2 antibody (10 μg/ml; clone CBR LFA1/2; BioLegend, catalog no. 366302). In total, 100,000 cells (50 μl) per well were used to seed cell-repellent 96-well plates (Greiner Bio-One, catalog no. 655970), which were incubated at 37°C for 1.5 hours to allow the formation of large aggregates. After incubation, the plate was shaken at 900 rpm for 30 s to disrupt weak, nonintegrin-mediated adhesion. Microscopy images were taken to document cell aggregation. Cells were then fixed by adding 50 μl of 4% paraformaldehyde (PFA, Santa Cruz Biotechnology, catalog no. sc-281692) to each well and incubating at room temperature for 30 min. The plate was shaken again at 900 rpm for 10 s, and the cell suspension was gently collected with a 1000-μl pipette tip and passed through a filter with 35-μm pores (Corning, catalog no. 352235) to separate single cells from aggregates. Aggregation was quantified by determining the percentage of cells retained on the filter (relative to input) by volumetric flow cytometry. The mean of two technical replicates (two wells) was used for analysis. Volumetric counting was performed on an Attune NxT Flow Cytometer (Thermo Fisher Scientific, RRID:SCR_019590), and data were analyzed with FlowJo (v10.10.0) (RRID:SCR_008520).

### LFA-1 activation in primary T cells

LFA-1 expression, conformational activation, and ligand binding were evaluated in primary T cells from patients and healthy donors after stimulation with inside-out or pharmacological integrin activators. PBMCs were thawed, rested for 2 hours, and resuspended in RPMI supplemented with 0.1% BSA before plating 1 × 10^5^ cells per well in V-bottom 96-well polypropylene plates (Greiner, catalog no. 651201). For conformational activation, Allophycocyanin-conjugated m24 antibody (a conformation-specific antibody that recognizes the open, high-affinity state of β2 integrins) was added to the cultures at 2 μg/ml (BioLegend, catalog no. 363410). For ICAM-1 binding, VLPs containing mNEON and displaying ICAM-1 were added to the wells (see the “ICAM-1 binding assays” section). Cells were then stimulated for 5 to 30 min with the following integrin activators: CXCL12 (500 ng/ml; 5 min), Mg^2+^/EGTA (5 mM/2 mM; 10 min), a β2-activating mAb (clone CBR LFA1/2; BioLegend, catalog no. 366302; 5 μg/ml; 10 min), PMA (100 ng/ml; 10 min), or anti-CD3/CD28 (30 min). Following stimulation, cells were immediately fixed with ice-cold 1% PFA (Santa Cruz Biotechnology, catalog no. sc-281692), washed, and stained for flow cytometric analysis.

### β2-integrin activation in neutrophils

The expression and conformational activation of β2 integrins were assessed in neutrophils from a patient with αL deficiency (P1) and age-matched healthy controls after stimulation with neutrophil chemoattractants (inside-out signals) or pharmacological integrin activators. Fresh whole blood was subjected to erythrocyte lysis (BioLegend, catalog no. 420301), after which leukocytes were resuspended in RPMI supplemented with 0.1% BSA and rested for 1 hour at 37°C. A total of 1 × 10^5^ leukocytes per well was plated in V-bottom 96-well polypropylene plates (Greiner, catalog no. 651201). Allophycocyanin-conjugated m24 antibody (BioLegend, catalog no. 363410), which binds the extended/open high-affinity conformation of β2 integrins, was added at 2 μg/ml. Cells were then stimulated for 10 min with the following integrin activators: the bacterial-derived peptide fMLP (100 nM, Sigma-Aldrich, catalog no. 47729), the chemokine CXCL8 (IL-8, 100 ng/ml, BioLegend, catalog no. 574202), LTB4 (100 nM, Cayman Chemical, catalog no. 20110), PMA (100 nM, Sigma-Aldrich, catalog no. P8139), or a β2-activating mAb (clone CBR LFA1/2; BioLegend, catalog no. 366302; 5 μg/ml). After stimulation, cells were immediately fixed with ice-cold 1% PFA (Santa Cruz Biotechnology, catalog no. sc-281692), washed, and stained for flow cytometric analysis.

### Adhesion assays

#### Immobilization of ICAM-3, VCAM-1, MAdCAM-1, and laminin

For the preparation of substrates for cell adhesion assays, flat-bottom 96-well tissue culture plates (Corning, catalog no. CLS3628) were coated with the following recombinant ligands: human ICAM-3-Fc (5 μg/ml; R&D Systems, catalog no. 715-IC-050), human VCAM-1–Fc (5 μg/ml; Sino Biological, catalog no. 10113-H02H), human MAdCAM-1-Fc (5 μg/ml; Thermo Fisher Scientific, catalog no. 50-210-6496), and laminin 511 (10 μg/ml; BioLamina, catalog no. LN511-0202). A human IgG1 (5 μg/ml; Sino Biological, catalog no. HG1K) was also used as a negative control. All ligands were diluted to the indicated concentrations in PBS containing calcium and magnesium (PBS^++^). Plates were incubated overnight at 4°C and then washed three times with PBS^++^ supplemented with 0.1% BSA. Plates were then blocked with PBS^++^ supplemented with 2% BSA for 1 hour at 37°C and washed once with PBS^++^ 0.1% BSA before cell seeding.

#### Immobilization of ICAM-1

For ICAM-1 immobilization, 96-well streptavidin-coated, BSA pre-blocked plates (Thermo Fisher Scientific, catalog no. 15121) were incubated with recombinant human biotinylated ICAM-1 (C-terminal Avi-tag; Sino Biological, catalog no. 10346-H49H-B), at a concentration of 2 μg/ml in PBS^++^. Plates were incubated at room temperature for 2 hours with shaking at 800 rpm and then washed three times with PBS^++^ 0.1% BSA before cell seeding.

#### Adhesion of T cells to immobilized integrin ligand

Jurkat cells or primary T cells were resuspended at a concentration of 0.5 × 10^6^ cells/ml in RPMI medium supplemented with 0.1% BSA, 5 mM MgCl_2_, and 2 mM EGTA. We then immediately added 100 μl of cell suspension (50,000 cells) to each of the coated wells or left an equal volume aside for input measurement. Plates were incubated at 37°C for 30 min. Nonadherent cells were removed by shaking the plate at 900 rpm for 10 s and then washing gently, twice with 100 μl of PBS^++^ supplemented with 0.1% BSA per wash. Adherent cells were detached by adding 50 μl of TrypLE (Gibco, catalog no. 12604013) and incubating for 10 min at 37°C. We then added 100 μl of RPMI medium supplemented with 10% FBS and transferred 150 μl of the resulting cell suspension to a V-bottom plate. Cells were fixed by adding 50 μl of 4% PFA (Santa Cruz Biotechnology, catalog no. sc-281692) to each well. For input samples, we added 50 μl of RPMI medium and 50 μl of 4% PFA to 100 μl of cell suspension.

#### Adhesion of HEK293T cells to immobilized ICAM-1 and ICAM-3

HEK293T cells were transfected with either WT αL or the αL variants reported in the general population, along with β2. They were mechanically harvested 60 hours posttransfection in the presence of EDTA. Cells were washed twice in PBS^++^ 0.1% BSA to remove residual EDTA and resuspended in DMEM medium supplemented with 0.1% BSA, 5 mM MgCl_2_, and 2 mM EGTA. We then immediately added 100 μl of cell suspension (50,000 cells) to each coated well or set aside the same volume for input measurement. Plates were incubated for 45 min at 37°C. Nonadherent cells were removed, and adherent cells were harvested as previously described for T cells.

#### Quantification of cell adhesion

The percentage of cells adhering to the plate was determined by volumetric flow cytometry. Cell counts were obtained with 50 μl of the suspension from coated wells and normalized against the corresponding count from 50 μl of the input well suspension. The mean of two technical replicates (two wells) was used for analysis. Volumetric counting was performed on an Attune NxT Flow Cytometer (Thermo Fisher Scientific, RRID:SCR_019590), and data were analyzed using FlowJo (v10.10.0) (RRID:SCR_008520).

### T cell migration assays

#### Immobilization of integrin ligands on Transwell membranes

Migration assays were performed with 96-well Transwell plates with 5.0-μm pores for Jurkat cells (Corning, catalog no. 3388) and 3.0-μm pores for T cell blasts (Corning, catalog no. 3385). Transwell inserts were coated with the following recombinant ligands: human ICAM-1–Fc (5 μg/ml; Sino Biological, catalog no. 10346-H02H), VCAM-1–Fc (5 μg/ml; Sino Biological, catalog no. 10113-H02H), and laminin 511 (10 μg/ml; BioLamina, catalog no. LN511-0202). A human IgG1 (5 μg/ml; Sino Biological, catalog no. HG1K) was used as a negative control. All ligands were diluted to the specified concentrations in PBS containing calcium and magnesium (PBS^++^). Inserts were incubated overnight at 4°C and then washed twice with PBS^++^ supplemented with 0.1% BSA. Inserts were then blocked by incubation with PBS^++^ supplemented with 2% BSA for 1 hour at 37°C. The blocked inserts were washed once with PBS^++^ 0.1% BSA before cell seeding.

#### Migration of T cells

Integrin αL KO Jurkat cells transduced with WT αL, patient variants, or an empty vector were serum-starved for 24 hours in RPMI medium supplemented with 0.1% BSA before the assay. T cell blasts from patients and controls were serum-starved under the same conditions for 12 hours. Cells were resuspended at a density of 1 × 10^6^ cells/ml in RPMI supplemented with 0.1% BSA, and 75 μl of the suspension (75,000 cells) was added to the upper chamber of the coated Transwell insert (Corning, catalog no. 3388 for Jurkat cells or catalog no. 3385 for primary T cells) or set aside for input measurement. The inserts were then placed on top of the lower chambers containing 235 μl of RPMI supplemented with 0.1% BSA and CXCL12 (100 ng/ml; Sino Biological, catalog no. 13511-HNCE). Transwells were incubated for 4 hours at 37°C. The inserts were then removed, and 150 μl (64%) of the contents of the lower chamber was transferred to a V-bottom plate. Cells were fixed by adding 50 μl of 4% PFA (Santa Cruz Biotechnology, catalog no. sc-281692) to each well. For input samples, 75 μl of RPMI medium and 50 μl of 4% PFA were added to 75 μl of cell suspension.

#### Quantification of cell migration

The percentage of cells migrating from the upper to the lower chamber was quantified using volumetric flow cytometry. Cell counts were obtained from 50 μl of the fixed lower chamber suspension, scaled up to estimate the number of cells present in the entire lower chamber, and normalized against the corresponding count from the fixed input suspension. The mean of two technical replicates (two Transwells) was used for analysis. Volumetric counting was performed on an Attune NxT Flow Cytometer (Thermo Fisher Scientific, RRID:SCR_019590), and data were analyzed with FlowJo (v10.10.0) (RRID:SCR_008520).

### Neutrophil migration assays

Neutrophil chemotaxis was assessed using 96-well Transwell plates with 3.0-μm pores (Corning, catalog no. 3385). Transwell inserts were blocked by incubation with PBS supplemented with 2% BSA for 1 hour at 37°C. The blocked inserts were washed once with PBS 0.1% BSA before cell seeding. Fresh whole blood from a patient with αL deficiency (P1) and age-matched healthy controls was subjected to erythrocyte lysis (BioLegend, catalog no. 420301). Cells were then resuspended in RPMI supplemented with 0.1% BSA and allowed to rest for 1 hour before use. For experiments involving integrin blockade, neutrophils were preincubated for 30 min at 37°C with antibodies against β2 integrins (TS1/18; 10 μg/ml; BioLegend, catalog no. 302115) or LFA-1 (TS1/22; 10 μg/ml; Thermo Fisher Scientific, catalog no. MA11A10).

A total of 75 μl of the cell suspension (75,000 cells) was added to the upper chamber of the Transwell insert or set aside for input measurement. The inserts were then placed on top of the lower chambers containing 235 μl of RPMI supplemented with 0.1% BSA and either fMLP (20 nM, Sigma-Aldrich, catalog no. 47729), the chemokine CXCL8 (IL-8; 20 ng/ml; BioLegend, catalog no. 574202), or LTB4 (100 nM; Cayman Chemical, catalog no. 20110). Transwells were incubated for 1 hour at 37°C. The inserts were then removed, and 150 μl (64%) of the contents of the lower chamber was transferred to a V-bottom plate. Cells were fixed by adding 50 μl of 4% PFA (Santa Cruz Biotechnology, catalog no. sc-281692) to each well. The proportion of neutrophils migrating from the upper to the lower chamber was quantified using volumetric flow cytometry, gating on the neutrophil population. The mean of two technical replicates (two Transwells) was used for analysis. Volumetric counting was performed on an Attune NxT Flow Cytometer (Thermo Fisher Scientific, RRID:SCR_019590), and data were analyzed with FlowJo (v10.10.0) (RRID:SCR_008520).

### T cell proliferation assay

PBMCs were labeled with carboxyfluorescein diacetate succinimidyl ester (CFSE) dye (Thermo Fisher Scientific, catalog no. C34554) according to the manufacturer’s instructions. Cells were used to seed 96-well plates at a density of 100,000 cells per well in RPMI medium supplemented with 10% FBS and were either left unstimulated or were stimulated with anti-CD3/CD28/CD2 antibodies (STEMCELL Technologies, catalog no. 109700, 1:40 dilution). Five days poststimulation, cells were harvested and surface-stained by incubation for 1 hour at 4°C with the following antibodies: anti-CD3-V450 (dilution: 1:200), clone UCHT1 (BD Biosciences, catalog no. 560365); anti-CD4-BV480 (dilution: 1:2500), clone SK3 (BD Biosciences, catalog no. 566104); anti-CD8-BV711 (dilution: 1:200), clone RPA-T8 (BD Biosciences, catalog no. 563677); anti–CD11a-peridinin–chlorophyll–protein (PerCP) (dilution: 1:1000), clone TS2/4 (BioLegend, catalog no. 350608); anti–CLA-PE (dilution: 1:200), clone HECA-452 (BioLegend, catalog no. 321311); anti–CCR7-PE-Cy7 (dilution: 1:200), clone G043H7 (BioLegend, catalog no. 353225); and anti–CD45RA-Allophycocyanin-H7 (dilution: 1:200), clone HI100 (BD Biosciences, catalog no. 560674), in the presence of Human TruStain FcX (BioLegend, AB_2818986) and True-Stain Monocyte Blocker (BioLegend, catalog no. 426102) to minimize nonspecific binding. Acquisition was performed with an Attune NxT Flow Cytometer (Thermo Fisher Scientific, RRID:SCR_019590), and data were analyzed with FlowJo (v10.10.0) (RRID:SCR_008520). Cryopreserved PBMCs from patients with αL deficiency (*n* = 6) and healthy controls (*n* = 14, including 8 travel controls) were used in these experiments. Similar results were obtained with cells stimulated with plate-bound anti-CD3 antibody (2 μg/ml; clone OKT3; BioLegend, catalog no. 317325) and anti-CD28 antibody (2 μg/ml; clone V-CD28. 05; BioLegend, catalog no. 302933) or PMA/ionomycin (5 ng/ml).

### Detection of cytokine production by intracellular flow cytometry

PBMCs were used to seed U-bottom 96-well plates at a density of 200,000 cells per well in 200 μl of RPMI medium supplemented with 10% FBS. Cells were either left unstimulated or were stimulated with anti-CD3/CD28/CD2 antibodies (STEMCELL Technologies, catalog no. 109700, 1:40 dilution). After incubation for 24 hours at 37°C, 100 μl of supernatant was collected, frozen, and stored at −20°C until use, and 100 μl of fresh medium supplemented with PMA (50 ng/ml; Sigma-Aldrich, catalog no. P8139), ionomycin (1 μM; Sigma-Aldrich, catalog no. I3909-1ML), and brefeldin A (1×; Tonbo, TNB-4506-L001) was added. The cells were incubated in this medium for 4 hours and were then harvested and stained with ViaKrome 808 viability dye (Beckman Coulter, 1:2000 in PBS) for 20 min at room temperature. Surface staining was then performed by incubating the cells for 1 hour at 4°C with the following antibodies: TCRγδ BUV661 (clone 11F2, BD Biosciences, catalog no. 750019, RRID:AB_2874238, 1:100 dilution), CLA PE (clone HECA-452, BioLegend, catalog no. 321311, 1:200 dilution), and CD11a PerCP (clone TS/4, BioLegend, catalog no. 350608, 1:200 dilution), in the presence of Human TruStain FcX (BioLegend, AB_2818986) and True-Stain Monocyte Blocker (BioLegend, catalog no. 426102) to minimize nonspecific binding. Cells were washed, fixed, and permeabilized with the Foxp3 Transcription Factor Staining Buffer Set (eBioscience) according to the manufacturer’s instructions. Permeabilized cells were incubated overnight at 4°C with the following antibodies: CD45 Alexa Fluor 350 (clone 2D1, FAB1430U, R&D Systems, AB_3646482, 1:200 dilution); CD56 BUV737 (clone TULY56, Thermo Fisher Scientific, catalog no. 367-0566-42, RRID:AB_2895975, 1:400 dilution); CD3 NovaFluor B610-70S (clone SK7, Thermo Fisher Scientific, AB_3098363, 1:2000 dilution); CD45RA BUV395 (clone 5H9, BD Biosciences, catalog no. 740315, RRID:AB_2740052, 1:12,000 dilution); CD8 BUV805 (clone SK1, BD Biosciences, catalog no. 612889, RRID:AB_2833078, 1:6000 dilution); CCR7 BV750 (clone G043H7, BioLegend, catalog no. 353253, RRID:AB_2800944, 1:400 dilution); FoxP3 RB780 (clone 236A/E7, BD Biosciences, catalog no. 569086, 1:200 dilution); CD4 cFluor BYG750 (clone SK3, Cytek, catalog no. SKU R7-20160, 1:6000 dilution); TNF Alexa Fluor 700 (clone Mab11, BioLegend, catalog no. 502928, AB_2561315, 1:1200 dilution); anti-CCR10-BB515 (dilution: 1:400), clone 1B5 (BD Biosciences, catalog no. 564769); anti–IL-9-PerCP-Cy5.5 (dilution: 1:200), clone MH9A4 (BioLegend, catalogno.507609);anti–granulocyte-macrophagecolony-stimulating factor (GM-CSF)–PE/Dazzle594 (dilution: 1:200), clone BVD2-21C11 (BioLegend, catalog no. 502317); anti–IL-22–Allophycocyanin (dilution: 1:600), clone 2G12A41 (BioLegend, catalog no. 366705); anti–IL-10–BV421 (dilution: 1:200), clone JES3-9D7 (BioLegend, catalog no. 501421); anti–IFN-γ–e450 (dilution: 1:2000), clone 4S.B3 (Thermo Fisher Scientific, catalog no. 48-7319-41); anti–IL-2–BV510 (dilution: 1:200), clone MQ1-17H12 (BioLegend, catalog no. 500337); anti–IL-4–BV605 (dilution: 1:200), clone MP42SD2 (BioLegend, catalog no. 500827); anti–IL-17A–BV786 (dilution: 1:200), clone BL168 (BioLegend, catalog no. 512337), in the presence of Human TruStain FcX (BioLegend, AB_2818986), True-Stain Monocyte Blocker (BioLegend, catalog no. 426102), and Cell-Blox Plus (Thermo Fisher Scientific, C001T06F01) to minimize nonspecific binding. Acquisition was performed with a Cytek Aurora Spectral Analyzer (RRID:SCR_019826). Data were analyzed with FlowJo software (v10.10.0) (SCR_008520). Cryopreserved PBMCs from patients with αL deficiency (*n* = 6) and healthy controls (*n* = 14, including 8 travel controls) were used in these experiments.

### Detection of secreted cytokines in a multiplex bead assay

The harvested supernatants of stimulated PBMCs were stored at −20°C until use. Cytokine concentrations were determined in multiplex bead assays with Human T Helper Cytokine Panel Version 2 (BioLegend, catalog no. 741028). This panel can be used to quantify 12 human cytokines—IL-2, IL-4, IL-5, IL-6, IL-9, IL-10, IL-13, IL-17A, IL-17F, IL-22, IFN-γ, and TNF—secreted by T_H_1, T_H_2, T_H_9, T_H_17, and T_H_22 cells. LEGENDPlex reagents (beads and antibodies) were diluted 1:4 in PBS supplemented with 1% BSA. The samples were analyzed by flow cytometry on an Attune NxT flow cytometer (RRID:SCR_019590), with 5000 beads acquired per sample. Data were analyzed with LEGENDplex Cloud-based Data Analysis Software (Qognit).

### Generation and maturation of monocyte-derived dendritic cells

CD14^+^ monocytes were isolated from freshly collected PBMCs obtained by Ficoll density gradient centrifugation (GE Healthcare). Positive selection was performed using CD14 MicroBeads (Miltenyi Biotec, catalog no. 130-097-052) according to the manufacturer’s instructions. Purified monocytes were resuspended in RPMI 1640 medium with GlutaMAX (Gibco), supplemented with 10% FBS, and plated at a density of 1 × 10^6^ cells/ml in six-well plates (2 × 10^6^ monocytes in 2 ml per well). Monocytes were differentiated into immature MDDCs by culturing them for 6 days in the presence of recombinant human GM-CSF (PeproTech, catalog no. 300-03) and IL-4 (PeproTech, catalog no. 200-04), each at 50 ng/ml. Cytokines were refreshed at day 3. On day 6, MDDCs were matured via Toll-like receptor stimulation. Half of the culture medium was removed and replaced with medium containing a 2× agonist mixture to obtain the final concentrations of R848 (2 μg/ml; Invivogen, catalog no. tlrl-r848-5) and lipopolysaccharide (0.1 μg/ml) from *Salmonella minnesota* (Invivogen, catalog no. tlrl-smlps). Cells were incubated with the maturation cocktail for 36 hours, after which fully matured dendritic cells were collected and used for allogenic reactions.

### APC-dependent T cell activation using allogeneic reactions

APC-dependent T cell activation was assessed using one-way “mixed lymphocyte reaction” (MLR) assays. Cryopreserved PBMCs from patients with αL deficiency and healthy controls (including travel controls) were cocultured with allogeneic MDDCs generated from healthy donors, as described above. Additional MLR assays were performed using PBMCs from healthy donors cocultured with MDDCs generated from P1.

Fresh PBMCs were also used to isolate both monocytes and T cells from the same healthy individual. Monocytes were differentiated into MDDCs. Meanwhile, T cells were electroporated to KO αL (see the “Gene KO” section). After electroporation, T cells were rested for 7 days to allow surface LFA-1 to disappear. During this time, monocytes completed their differentiation into MDDCs. On day 7, the MDDCs and αL KO T cells from different individuals were cocultured to perform MLR assays.

For quantification of cytokine production by intracellular staining, 200,000 cells were cocultured in a final volume of 200 μl in U-bottom 96-well plates at the indicated MDDC:T cell ratios. Cultures were maintained for 24 hours, with brefeldin A (1×; Tonbo, TNB-4506-L001) added during the final 6 hours. At 24 hours, cells were harvested and stained with Zombie NIR Fixable Viability Dye (BioLegend, catalog no. 423106, 1:5000 in PBS) for 10 min at room temperature. Surface staining was then performed by incubating the cells for 30 min at 4°C with the following antibodies: anti–integrin αL (CD11a)-PE (dilution: 1:1000), clone TS2/4 (BioLegend, catalog no. 350606/RRID:AB_10660753) and anti–CD4-BV480 (dilution: 1:−1000), clone SK3 (BD Biosciences, catalog no. 566104). Cells were washed, fixed, and permeabilized with the Foxp3 Transcription Factor Staining Buffer Set (eBioscience) according to the manufacturer’s instructions. Permeabilized cells were incubated overnight at 4°C with the following antibodies: CD45 Alexa Fluor 350 (clone 2D1, FAB1430U, R&D Systems, AB_3646482, 1:200 dilution); CD3 NovaFluor B610-70S (clone SK7, Thermo Fisher Scientific, AB_3098363, 1:1000 dilution); CD45RA BUV395 (clone 5H9, BD Biosciences, catalog no. 740315, RRID:AB_2740052, 1:12,000 dilution); CD8 BUV805 (clone SK1, BD Biosciences, catalog no. 612889, RR-ID:AB_2833078, 1:12,000 dilution); CCR7 BV750 (clone G043H7, BioLegend, catalog no. 353253, RRID:AB_2800944, 1:400 dilution); TNF Alexa Fluor 700 (clone Mab11, BioLegend, catalog no. 502928, AB_2561315, 1:1200 dilution); anti–IFN-γ–e450 (dilution: 1:2000), clone 4S.B3 (Thermo Fisher Scientific, catalog no. 48-7319-41), in the presence of Human TruStain FcX (BioLegend, AB_2818986), True-Stain Monocyte Blocker (BioLegend, catalog no. 426102), and CellBlox Plus (Thermo Fisher Scientific, C001T06F01) to minimize nonspecific binding. Acquisition was performed with a Cytek Aurora Spectral Analyzer (RRID:SCR_019826). Data were analyzed with FlowJo software (v10.10.0) (SCR_008520).

For proliferation assays, T cells were labeled with CFSE (Thermo Fisher Scientific, catalog no. C34554; dilution: 1:1000 in PBS) before coculture with allogeneic MDDCs at the indicated cell ratios. Cells were plated in U-bottom 96-well plates at 100,000 cells per well in a final volume of 200 μl of RPMI medium supplemented with 10% FBS and low-dose IL-7 (2 ng/ml). On day 3, half of the culture medium was replaced with fresh medium. On day 6, cells were harvested and stained with Zombie NIR Fixable Viability Dye (BioLegend, catalog no. 423106; 1:5000 in PBS) for 10 min at room temperature. Surface staining was then performed by incubating the cells for 30 min at 4°C with the following antibodies: anti–integrin αL (CD11a)-PE (dilution: 1:1000), clone TS2/4 (BioLegend, catalog no. 350606/RRID:AB_10660753) and anti–CD4-BV480 (dilution: 1:1000), clone SK3 (BD Biosciences, catalog no. 566104). Cells were washed, fixed, and permeabilized with the Foxp3 Transcription Factor Staining Buffer Set (eBioscience) according to the manufacturer’s instructions. Permeabilized cells were incubated overnight at 4°C with the following antibodies: CD3 NovaFluor B610-70S (clone SK7, Thermo Fisher Scientific, AB_3098363, 1:1000 dilution); CD45RA BUV395 (clone 5H9, BD Biosciences, catalog no. 740315, RRID:AB_2740052, 1:16,000 dilution); CD8 BUV805 (clone SK1, BD Biosciences, catalog no. 612889, RRID:AB_2833078, 1:16,000 dilution); CCR7 BV750 (clone G043H7, BioLegend, catalog no. 353253, RRID:AB_2800944, 1:400 dilution) in the presence of Human TruStain FcX (BioLegend, AB_2818986), True-Stain Monocyte Blocker (BioLegend, catalog no. 426102), and CellBlox Plus (Thermo Fisher Scientific, C001T06F01) to minimize nonspecific binding. Acquisition was performed with a Cytek Aurora Spectral Analyzer (RRID:-SCR_019826). Data were analyzed with FlowJo software (v10.10.0) (SCR_008520).

### Peptide stimulation assays

Antigen-specific T cell activation was assessed by short-term peptide stimulation of freshly isolated PBMCs. Among patients with αL deficiency, only P1 presented with a lesion (cSCC) caused by a high-risk α-HPV (HPV18). Fresh blood from P1 was therefore used to assess circulating HPV18-specific T cell responses. As P1 does not carry human leukocyte antigen (HLA) alleles linked to known immunodominant HPV18 epitopes, we used overlapping peptide pools spanning HPV18 E6 (Miltenyi Biotec, catalog no. 130-096-005) and HPV18 E7 (Miltenyi Biotec, catalog no. 130-095-996). The CEFSX Ultra SuperStim Pool (JPT, catalog no. PM-CEFSX-1) was used as a positive control for antigen-specific T cell stimulation. This pool contains 202 peptide epitopes covering multiple HLA subtypes and a wide range of infectious agents, including common viral pathogens (e.g., HSV-1, EBV, CMV, SARS-CoV-2, and influenza A), vaccine antigens (e.g., measles, rubella, and tetanus), and additional pathogens such as *Haemophilus influenzae*, *Helicobacter pylori*, and *Toxoplasma gondii*.

PBMCs were resuspended at 5 × 10^6^ cells/ml in RPMI 1640 with GlutaMAX supplemented with 5% heat-inactivated human AB serum (Sigma-Aldrich, catalog no. H6914) and 1 mM sodium pyruvate (Thermo Fisher Scientific, catalog no. 11360070). A total of 1 × 10^6^ cells (200 μl) was distributed into U-bottom 96-well plates and rested for 2 hours before stimulation. Peptides were added at a final concentration of 1 μg of each peptide per milliliter, together with brefeldin A (1×; Tonbo, TNB-4506-L001). Unstimulated wells received an equivalent volume of dimethyl sulfoxide. Cells were stimulated for 16 hours at 37°C, then harvested, and stained with Zombie NIR Fixable Viability Dye (BioLegend, catalog no. 423106; 1:5000 in PBS) for 5 min at room temperature. Surface staining was then performed by incubating the cells for 30 min at 4°C with the following antibodies: anti–integrin αL (CD11a)-PE (dilution: 1:1000), clone TS2/4 (BioLegend, catalog no. 350606/RRID:AB_10660753) and anti–CD4-BV480 (dilution: 1:1000), clone SK3 (BD Biosciences, catalog no. 566104). Cells were washed, fixed, and permeabilized with the Foxp3 Transcription Factor Staining Buffer Set (eBioscience) according to the manufacturer’s instructions. Permeabilized cells were incubated overnight at 4°C with the following antibodies: CD3 NovaFluor B610-70S (clone SK7, Thermo Fisher Scientific, AB_3098363, 1:1000 dilution); CD45RA BUV395 (clone 5H9, BD Biosciences catalog no. 740315, RRID:AB_2740052, 1:16,000 dilution); CD8 BUV805 (clone SK1, BD Biosciences, catalog no. 612889, RRID:AB_2833078, 1:16,000 dilution); CCR7 BV750 (clone G043H7, BioLegend, catalog no. 353253, RRID:AB_2800944, 1:400 dilution); TNF Alexa Fluor 700 (clone Mab11, BioLegend, catalog no. 502928, AB_2561315, 1:1000 dilution); anti–IFN-γ–e450 (dilution: 1:1000), clone 4S.B3 (Thermo Fisher Scientific, catalog no. 48-7319-41), in the presence of Human TruStain FcX (BioLegend, AB_2818986), True-Stain Monocyte Blocker (BioLegend, catalog no. 426102), and CellBlox Plus (Thermo Fisher Scientific, C001T06F01) to minimize nonspecific binding. Acquisition was performed with a Cytek Aurora Spectral Analyzer (RRID:SCR_019826). Data were analyzed with FlowJo software (v10.10.0) (SCR_008520).

### Deep immunophenotyping of whole blood cells

Deep immunophenotyping of whole blood cells was performed using mass cytometry (CyTOF). Fresh whole-blood samples (200 μl) were obtained from patients (*n* = 4) and controls (*n* = 51) and processed within 24 hours of collection. Custom-designed antibody panels, detailed in table S10, were used according to the manufacturer’s instructions (Fluidigm). After overnight dead cell staining, cells were frozen and stored at −80°C until acquisition on a Helios mass cytometer (Fluidigm). Data analysis was performed with OMIQ software.

### Full-spectrum flow cytometry

A 38-color full-spectrum flow cytometry panel was designed and validated for the in-depth characterization of circulating tissue-specific T cells and integrin expression across leukocyte subsets. Fresh whole-blood samples (1 million cells) or cryopreserved PBMCs (0.5 to 1 million) from patients (*n* = 6) and controls (*n* = 22) were stained. Dead cells were labeled by incubation with ViaKrome 808 (Beckman Coulter, 1:2000 dilution) in PBS at room temperature for 20 min. Surface staining was then performed by incubating the cells for 1 hour at 4°C with the antibodies detailed in table S11 (surface), in the presence of Human TruStain FcX (BioLegend, RRID:AB_2818986), True-Stain Monocyte Blocker (BioLegend, catalog no. 426102), and CellBlox Plus (Thermo Fisher Scientific, catalog no. C001T06F01) to minimize nonspecific binding. The cells were washed and then fixed and permeabilized with the Foxp3 Transcription Factor Staining Buffer Set (eBioscience) according to the manufacturer’s instructions. Permeabilized cells were incubated overnight at 4°C with the antibodies detailed in table S11 (intracellular). Acquisition was performed with a Cytek Aurora Spectral Analyzer (RRID:-SCR_019826). Data were analyzed with FlowJo software (v10.10.0) (SCR_008520). Gating strategy for spectral flow cytometry is provided in data file S6.

### Single-cell transcriptomics and surface epitope detection (CITE-seq)

Cryopreserved PBMCs from patients with αL deficiency (P1 and P2) and eight healthy adult controls were analyzed by CITE-seq, a method for simultaneous single-cell transcriptomic sequencing and surface protein analysis. Thawed cells were washed with medium, filtered through a 70-μm mesh MACS SmartStrainer (Miltenyi Biotec, catalog no. 130-098-462), and stained by incubation with Total-Seq-C Human Universal Cocktail, V1.0 (BioLegend, catalog no. 399905) for 30 min at 4°C, according to the manufacturer’s protocol. The cocktail contains oligomer-conjugated antibodies targeting 130 different cell–surface proteins and seven isotypic controls. Cells were washed three times with PBS supplemented with 0.2% BSA and subjected to single-cell capture on the 10X Genomics Chromium platform. Libraries were generated with the Chromium Next GEM Automated Single-Cell 5′ Kit v2 with Feature Barcoding (10X Genomics) according to the manufacturer’s protocol. The purified libraries were sequenced on the Illumina NovaSeq 6000 System (S4 flow cell, 150-bp paired-end reads), with a median depth of 50,000 reads per cell for gene expression.

### Alignment and demultiplexing

We performed alignment, filtering, barcode counting, and unique molecular identifier (UMI) counting on the gene expression and antibody barcode libraries and sequence assembly and paired clonotype calling on the V(D)J libraries, for each sample with CellRanger (v7.2.0), using the cellranger multicommand with default parameters. The hg38 reference transcriptome and a VDJ reference were obtained from CellRanger (transcriptome: https://cf.10xgenomics. com/supp/cell-exp/refdata-gex-GRCh38-2020-A.tar.gz, VDJ reference: https://cf.10xgenomics.com/supp/cell-vdj/refdata-cellranger-vdj-GRCh38-alts-ensembl-7.1.0.tar.gz). For the demultiplexing of samples, the bam file produced from the alignment for each multiplexed sample was filtered using samtools (v1.9), using the samtools view command with parameters -S -b -q 10 -F 3844. The filtered bam file was then deduplicated with umi tools (1.1.0), using the umi tools dedup command with parameters --extract-umi-method=tag--umi-tag=UB --cell-tag=CB. SNVs were called on the filtered and deduplicated bam file with freebays (v1.3.2) (95), using the parameters -iXu -C 2 -q 1. Barcodes within multiplexed samples were then assigned to specific samples with scSPLIT (v1.0.9), using common variants obtained from https://melbourne.figshare.com/articles/dataset/Common_SNVS_hg38/17032163.

### Quality control

Quality control was performed with Seurat 5.1.0 in R 4.3.3 (https://ropensci.org/blog/2021/11/16/how-to-cite-r-and-r-packages/) for each sample separately. We first filtered out cells that deviated by more than 5 median absolute deviations in either direction from the median log mRNA and protein library size and/or with more than 10% mitochondrial counts. We then removed any proteins in the sample with counts that were an order of magnitude below the lowest count of the isotype controls. We normalized and denoised protein counts with the Descaled by Background package (v1.0.4), using a background count matrix obtained from droplets that did not capture cells. The following parameters were used for the dsb normalization function: denoise.counts = TRUE, use.isotype.control = TRUE, quantile.clipping = TRUE, scale.factor = “mean.subtract.” We removed ambient RNA counts with SoupX (1.6.2), using a background count matrix obtained from droplets that did not capture cells and clusters defined with Seurat’s FindClusters function with the weighted nearest neighbor (WNN) graph using the Leiden algorithm and a resolution of 0.5. We estimated the contamination fraction with SoupX’s autoEstCont function with default parameters and adjusted the RNA count matrix with SoupX’s adjustCounts function with the parameter roundToInt = TRUE. We then predicted and removed doublets with scDbl finder (1.16.0), with a doublet rate set to 0.076. Multiplex samples were then split according to the barcode assignment from scSPLIT and doublets predicted by sc-SPLIT were removed.

### Integration, clustering, and annotation

We normalized the RNA counts for all patients and controls separately with Seurat’s scTransform function and then integrated the samples with Seurat’s Reciprocal Principal Components Analysis (RPCA) workflow, using the control samples designated as the reference datasets. We then integrated the Denoised and Scaled by Background (DSB)-normalized and denoised protein counts for all patients and controls according to the same workflow, again with the control samples designated as the reference datasets. We constructed a WNN graph with Seurat’s FindMultimodalNeightbors function and then used Seurat’s FindClusters function with the WNN graph, using the Leiden algorithm and a resolution of 5. We then annotated the cells from all samples using a PBMC CITE-seq reference (https://zenodo.org/records/7779017), using Seurat’s FindTransferAnchors and MapQuery functions. We merged cells that were labeled as CD4 T central memory and CD4 T effector memory as CD4 memory cells and merged the groups of cells labeled as CD8 T central memory and CD8 T effector memory into a single CD8 memory cells group. Rare cell populations labeled as doublets, AXL^+^ SIGLEC6^+^ dendritic cells (ASDC), erythrocytes, hematopoietic stem and progenitor cells (HSPC), innate lymphoid cells (ILC), and platelets were excluded from the scRNA-seq dataset. This decision was based on the marker expression profiles obtained by CITE-seq, which indicated that these labels were misclassifications.

### Compositional analysis

We extracted the number of cells for each leukocyte annotation to generate a table of cell counts per cell type for each patient and control. We then performed compositional analysis with sc-Coda (v0.1.9), using cells annotated as “B naïve” as the reference cell type.

### Pseudobulk differential expression analysis

We aggregated the raw transcript counts of each gene within each reference annotated cell type with Seurat’s Aggregate Expression function. We then analyzed differential expression between patient and control cells with DESeq2 (v1.42.1) within Seurat’s FindMarkers function. We considered a gene to be differentially expressed within a cell type if its Benjamini-Hochberg adjusted *P* value was less than 0.05 and the absolute magnitude of its log_2_ fold change was greater than 1.

### Expression levels for integrins and anergy markers (protein)

DSB-normalized and denoised protein counts were plotted as violin plots with Seurat’s VlnPlot function.

### Analysis of the scTCR-seq repertoire

High-level annotations of each high-confidence contig from the cell-associated barcodes output of CellRanger multi were used for TCR analysis. Clonotypes were assigned on the basis of identical CDR3 amino acid sequences for both the TRA and TRB chains. Tree map (v2.4.4) plots were used to illustrate clonal expansion. Repertoire diversity metrics were then assessed by calculating species richness, the Gini-Simpson index, and Shannon entropy with vegan (v2.6.8). We accounted for sample size variability within memory populations by iteratively subsampling each sample (nboots = 100) with replacement, selecting up to 100 observations or clonotypes per iteration. Diversity metrics were computed with the Gini-Simpson index, and results were aggregated by sample and bootstrap index for analysis. This approach ensured robust diversity estimation while minimizing sampling bias. Clonotype distributions and diversity metrics were compared between patients and healthy controls, and publicly available TCR databases (Immune Epitope Database) were used to infer potential antigen specificity by TCRMatch (44). All analyses were conducted in R (v4.3.3).

### Skin digestion and T cell counts

Four-millimeter punch biopsy samples of healthy skin were obtained from the inner upper arm or thigh of patients with αL deficiency and healthy controls. Biopsy specimens were transported in complete RPMI medium (10% FBS and 1% penicillin-streptomycin) within 24 hours. Upon arrival, tissues were washed with PBS to remove residual blood and incubated in 500 μl of 1× dispase solution (STEMCELL Technologies, catalog no. 07923) in 24-well plates for 2 hours at 37°C, with agitation every 30 min. The epidermis was carefully peeled away from the dermis. The dermis was minced into small fragments, added to the same dispase solution supplemented with collagenase (Roche, catalog no. 1088866001), and digested for 12 hours at 37°C. During this period, the epidermis was stored at 4°C in transport medium. The epidermis was then washed with PBS to eliminate serum and digested with 500 μl of trypsin-EDTA (Thermo Fisher Scientific, catalog no. 25200056) for 45 min at 37°C. The epidermal digestion was quenched by the addition of FBS, and the dermal digestion was stopped by the addition of 6 mM EDTA. Digested samples were dissociated by repeated pipetting until complete tissue dissolution. The resulting cell suspension was filtered through a 35-μm mesh (Corning, catalog no. 352235). Samples were centrifuged (350*g*, 10 min, 4°C), and the pellet was resuspended in PBS. We used 10% of the resulting cell suspension to obtain absolute cell counts by volumetric flow cytometry in the presence of 4′,6-diamidino-2-phenylindole (10 ng/ml; Thermo Fisher Scientific, catalog no. 62248) and anti–HLA-ABC antibody (BioLegend, catalog no. 311409), without further washing. The remaining cells were permeabilized and stained with anti-CD3 (BD Biosciences, catalog no. 560365), anti-CD4 (BD Biosciences, catalog no. 566104), and anti-CD8 (BD Biosciences, catalog no. 563677) antibodies. Volumetric cell counting was performed with an Attune NxT Flow Cytometer (Thermo Fisher Scientific, RRID:SCR_019590), and data were analyzed with FlowJo software (v10.10.0, RRID:SCR_008520).

### Skin digestion and integrin expression

A 28-color full-spectrum flow cytometry panel was designed for the detailed characterization of cutaneous T cells and their integrin expression profiles. We obtained 4-mm punch biopsy specimens of healthy skin from the inner upper arm of two healthy adults. Cell suspensions prepared from fully digested skin, as described above, were used directly for staining. PBMCs were obtained from the same donors and digested simultaneously. Dead cells were labeled by incubation with ViaKrome 808 (Beckman Coulter, 1:2000 dilution) in PBS at room temperature for 10 min. Surface staining was then performed by incubating the cells at 4°C for 1 hour with the following antibodies: anti–CD62L-BUV496 (dilution: 1:100), clone DREG-56 (BD Biosciences, catalog no. 741155); anti–TCRγδ-BUV661 (dilution: 1:100), clone 11F2 (BD Biosciences, catalog no. 750019); anti-CD18-BUV805 (dilution: 1:100), clone 6.7 (BD Biosciences, catalog no. 749381); anti-CD103 (integrin αE; ITGAE)-BV605 (dilution: 1:400), clone Ber-ACT8 (BioLegend, catalog no. 350217); anti-CD49d (ITGA4)-BV785 (dilution: 1:200), clone 9F10 (BioLegend, catalog no. 304344); anti-OX40-PE-Cy7 (dilution: 1:−100), clone OX-86 (BioLegend, catalog no. 119416); anti-CD29 (integrin β1; ITGB1)-PE-Dazzle594 (dilution: 1:400), clone TS2/16 (BioLegend, catalog no. 303031); anti-ITGB7-Allophycocyanin (dilution: 1:400), clone FIB504 (BioLegend, catalog no. 321207), in the presence of Human TruStain FcX (BioLegend, AB_2818986), TrueStain Monocyte Blocker (BioLegend, catalog no. 426102), and CellBlox Plus (Thermo Fisher Scientific, C001T06F01) to minimize nonspecific binding. The cells were washed, then fixed, and permeabilized with the Foxp3 Transcription Factor Staining Buffer Set (eBioscience) according to the manufacturer’s instructions. Permeabilized cells were incubated overnight at 4°C with the following antibodies: anti–CD45RA-BUV395 (dilution: 1:5000), clone 5H9 (BD Biosciences, catalog no. 740315); anti–CD45-AF350 (dilution: 1:−100), clone 2D1 (R&D Systems, catalog no. FAB1430U); anti–CD11b-BUV563 (dilution: 1:800), clone ICRF44 (Thermo Fisher Scientific, catalog no. 365-0118-42); anti–CCR4-BUV615 (dilution: 1:100), clone 1G1 (BD Biosciences, catalog no. 613000); anti–CD56-BUV737 (dilution: 1:200), clone TULY56 (Thermo Fisher Scientific, catalog no. 367-0566-42); anti–CD11c-eFluor 450 (dilution: 1:200), clone 3.9 (Thermo Fisher Scientific, catalog no. 48-0116-42); anti-CCR6-BV711 (dilution: 1:100), clone G034E3 (BioLegend, catalog no. 353436); anti–CCR7-BV750 (dilution: 1:200), clone G043H7 (BioLegend, catalog no. 353253); anti-CCR10-BB515 (dilution: 1:−200), clone 1B5 (BD Biosciences, catalog no. 564769); anti–CD14-Spark Blue 550 (dilution: 1:5000), clone 63D3 (BioLegend, catalog no. 367147); anti–CD8-NovaFluor Blue 585 (dilution: 1:100), clone OKT-8 (Thermo Fisher Scientific, catalog no. H003T02B04); anti–CD3-NovaFluor B610-70S (dilution: 1:800), clone SK7 (Thermo Fisher Scientific, catalog no. H028T03B06); anti-CD11a (ITGAL)-PerCP (dilution: 1:100), clone TS2/4 (BioLegend, catalog no. 350608); anti–FoxP3-RB780 (dilution: 1:200), clone 236A/E7 (BD Biosciences, catalog no. 569086); anti–CLA-PE (dilution: 1:100), clone HECA-452 (BioLegend, catalog no. 321311); anti-CD69 intra–PE-Cy5 (dilution: 1:800), clone FN50 (BioLegend, catalog no. 310908); anti–CD4-cFluor BYG750 (dilution: 1:2000), clone SK3 (CYTEK, catalog no. SKU R7-20160); anti–CXCR3-PE-Fire810 (dilution: 1:−100), clone G025H7 (BioLegend, catalog no. 353760); anti–CD127-NovaFluor Red 710 (dilution: 1:100), clone eBioRDR5 (Thermo Fisher Scientific, catalog no. H017T03R04). Acquisition was performed with a Cytek Aurora Spectral Analyzer (RRID:SCR_019826). Data were analyzed with FlowJo software (v10.10.0) (SCR_008520). Cutaneous T cell subsets were defined as follows: CD8 T cells (CD3^+^TCRγδ^−^CD56^−^CD8), CD4 T cells (CD3^+^ TCRγδ^−^ CD4 FoxP3^−^), T_reg_ cells (CD3^+^CD4^+^FoxP3^+^CD127^−^CD25^+^), γδ T cells (CD3^+^ TCRγδ^+^), skin-resident memory T cells (T_RM_; CD69^+^ CCR7^−^CD45RA^−^), recirculating memory T cells (T_RECIRC_; CD69^−^ CCR7^−^CD45RA^−^), and migratory memory cells (CD69^−^CCR7^+^ CD62L^−^).

### Analysis of T cell subsets in patient skin

We obtained 4-mm punch biopsy specimens of skin from the upper arm of patient P1 and three age-matched healthy adults. Cell suspensions prepared from fully digested skin, as described above, were used directly for staining. Dead cells were labeled by incubation with Zombie NIR Fixable Viability Dye (BioLegend, catalog no. 423106, 1:2000 in PBS) for 5 min at room temperature. Surface staining was then performed by incubating the cells for 60 min at 4°C with the following antibodies: anti–integrin αL (CD11a)-PE (dilution: 1:200), clone TS2/4 (BioLegend, catalog no. 350606/RRID:AB_10660753); anti–integrin β2 (CD18)-AF700 (dilution: 1:200), clone TS1/18 (BioLegend, catalog no. 302123); anti–CD4-BV480 (dilution: 1:200), clone SK3 (BD Biosciences, catalog no. 566104); anti–CD62L-BUV496 (dilution: 1:100), clone DREG-56 (BD Biosciences, catalog no. 741155); anti–TCRγδ-BUV661 (dilution: 1:50), clone 11F2 (BD Biosciences, catalog no. 750019); anti-CD103 (ITGAE)-BV605 (dilution: 1:200), clone Ber-ACT8 (BioLegend, catalog no. 350217); anti-CD49d (ITGA4)-BV785 (dilution: 1:200), clone 9F10 (BioLegend, catalog no. 304344); anti-CD29 (ITGB1)-PE-Dazzle594 (dilution: 1:200), clone TS2/16 (BioLegend, catalog no. 303031); anti–ITGB7-Allophycocyanin (dilution: 1:200), clone FIB504 (BioLegend, catalog no. 321207); anti–CLA-PE (dilution: 1:100), clone HECA-452 (BioLegend, catalog no. 321311); anti–CD127-Allophycocyanin/Fire 750 (dilution: 1:100), clone A019D5 (BioLegend, catalog no. 351350), in the presence of Human TruStain FcX (BioLegend, AB_2818986), True-Stain Monocyte Blocker (BioLegend, catalog no. 426102), and CellBlox Plus (Thermo Fisher Scientific, C001T06F01) to minimize nonspecific binding. The cells were washed, then fixed, and permeabilized with the Foxp3 Transcription Factor Staining Buffer Set (eBioscience) according to the manufacturer’s instructions. Permeabilized cells were incubated overnight at 4°C with the following antibodies: anti–CD45RA-BUV395 (dilution: 1:5000), clone 5H9 (BD Biosciences, catalog no. 740315); anti–CD45-AF350 (dilution: 1:100), clone 2D1 (R&D Systems, catalog no. FAB1430U); anti–CD11b-BUV563 (dilution: 1:400), clone ICRF44 (Thermo Fisher Scientific, catalog no. 365-0118-42); anti-CD56-BUV737 (dilution: 1:200), clone TULY56 (Thermo Fisher Scientific, catalog no. 367-0566-42); anti–CD11c-eFluor 450 (dilution: 1:400), clone 3.9 (Thermo Fisher Scientific, catalog no. 48-0116-42); anti–CCR7-BV750 (dilution: 1:200), clone G043H7 (BioLegend, catalog no. 353253); anti–CD3-NovaFluor B610-70S (dilution: 1:1000), clone SK7 (Thermo Fisher Scientific, catalog no. H028T03B06); anti–FoxP3-RB780 (dilution: 1:200), clone 236A/E7 (BD Biosciences, catalog no. 569086); anti-CD69 intra–PE-Cy5 (dilution: 1:1000), clone FN50 (BioLegend, catalog no. 310908). Acquisition was performed with a Cytek Aurora Spectral Analyzer (RRID:SCR_019826). Data were analyzed with FlowJo software (v10.10.0) (SCR_008520). Cutaneous T cell subsets were defined as follows: CD8 T cells (CD3^+^ TCRγδ^−^ CD56^−^ CD8), CD4 T cells (CD3^+^ TCRγδ^−^ CD4 FoxP3^−^), T_reg_ cells (CD3^+^ CD4 FoxP3^+^ CD127^−^), γδ T cells (CD3^+^ TCRγδ^+^), skin-resident memory T cells (T_RM_; CD69^+^CCR7^−^CD45RA^−^), recirculating memory T cells (T_RECIRC_; CD69^−^CCR7^−^CD45RA^−^), and migratory memory cells (CD69^−^CCR7^+^CD62L^−^).

### Detection of HPV transcripts by scRNA-seq

Keratinocytes obtained from the digested biopsy specimens of P1 or healthy donors (*n* = 4) (as described above) were analyzed by scRNA-seq to detect HPV replication. The scRNA-seq libraries were generated with the Chromium Next GEM Single-Cell 3′ Reagent Kit v3.1 according to the manufacturer’s protocol, and the purified libraries were sequenced on a NovaSeq 6000 (Illumina) machine at a depth of ~60,000 reads per cell. Demultiplexed FASTQ files from scRNA-seq were aligned to a custom-built genome comprising hg38 and all HPV reference genomes, with standard alignment protocols. The expression matrices were integrated with the Seurat package (v4.3.0) in R. Initial quality control was performed manually based on standard metrics to filter out low-quality data. The filtered data were further integrated with Harmony. Keratinocyte subset clusters were identified on the basis of the expression of the following genes: *ITGA6*, *KRT5*, *KRT14*, *SPINK5*, *DSC1*, and *KRT2*. Feature plots showing HPV expression in keratinocytes were generated with Seurat in R.

### Statistical analysis

Statistical analyses were performed with GraphPad Prism (v10.4.0, RRID:SCR_002798) and are detailed in the corresponding figure legends. The normality of the distribution of each dataset was assessed in Shapiro-Wilk normality tests. For pairwise comparisons of normally distributed datasets, hypotheses were tested in Student’s *t* test (unpaired two-tailed tests) or, when variances were unequal, unpaired *t* tests with Welch’s correction. We used Holm-Šidák correction to adjust *t* tests for multiple comparisons. In comparisons of more than two groups with normally distributed datasets, hypotheses were tested in ordinary one-way analysis of variance (ANOVA) with Tukey’s test for multiple comparisons or Brown-Forsythe Welch’s ANOVA with Dunnett’s test for multiple comparisons, depending on the number of conditions and the experimental setup. For pairwise comparisons of nonnormally distributed datasets, statistical significance was determined in Mann-Whitney tests with Holm-Šidák correction for multiple comparisons. In comparisons of more than two groups with nonnormally distributed datasets, the Kruskal-Wallis test was performed followed by Dunn’s test for multiple comparisons. For comparisons involving two independent variables, two-way ANOVA was used with correction for multiple comparisons. Dunnett’s correction was used when comparing multiple groups to a single control group. Tukey’s correction was used for all pairwise comparisons among groups. *P* values or adjusted *P* values below 0.05 were considered significant for all statistical tests.

## Supplementary Material

Supplementary Data 2

Supplementary Data 1

Supplementary Data 3

Supplementary Data 4

Supplementary Data 7

Supplementary Data 6

Supplementary Data 5

Supplementary Table 10

Supplementary Table 9

Supplementary Table 11

Supplementary Table 8

Supplementary Table 7

Supplementary Table 6

Supplementary Table 5

Supplementary Table 4

Supplementary Table 3

Supplementary Table 2

Supplementary Table 1

Supplementary Fig 8

Supplementary Fig 6

Supplementary Fig 7

Supplementary Fig 4

Supplementary Fig 5

Supplementary Fig 3

Supplementary Fig 1

Supplementary Fig 2

Supplementary Fig 9

The PDF file includes:

Figs. S1 to S9

Tables S1 to S11

Legends for data files S1 to S7

Other Supplementary Material for this manuscript includes the following:

Data files S1 to S7

MDAR Reproducibility Checklist

## Data Availability

Tabulated data underlying Figs. 1 to 8 and figs. S1 to S8 are provided in data file S7. Uncropped immunoblots are provided in data file S5. Gating strategy for spectral flow cytometry is provided in data file S6. RNA-seq data are deposited at the SRA database under accession number PRJNA1415184. All other data needed to evaluate the conclusions in the paper are present in the paper or the Supplementary Materials. Plasmids encoding full-length *ITGAL* (WT or variants) and *ICAM1* (WT or E34A), αL KO Jurkat cells, T cell blasts from the patients and their TC were generated by the authors and are available upon reasonable request under a material transfer agreement with the Rockefeller University. Primer sequences are provided in data file S4. No original code was developed for this study. All other materials are commercially available as described in Materials and Methods.
