# Supplementary figures and images for: Human LFA-1 governs T cell immune surveillance of the skin
Source: Sci Immunol. Author manuscript; Available in PMC 2026 May 13. (PMC13171165; doi:10.1126/sciimmunol.adz8360)

# Gating Strategy

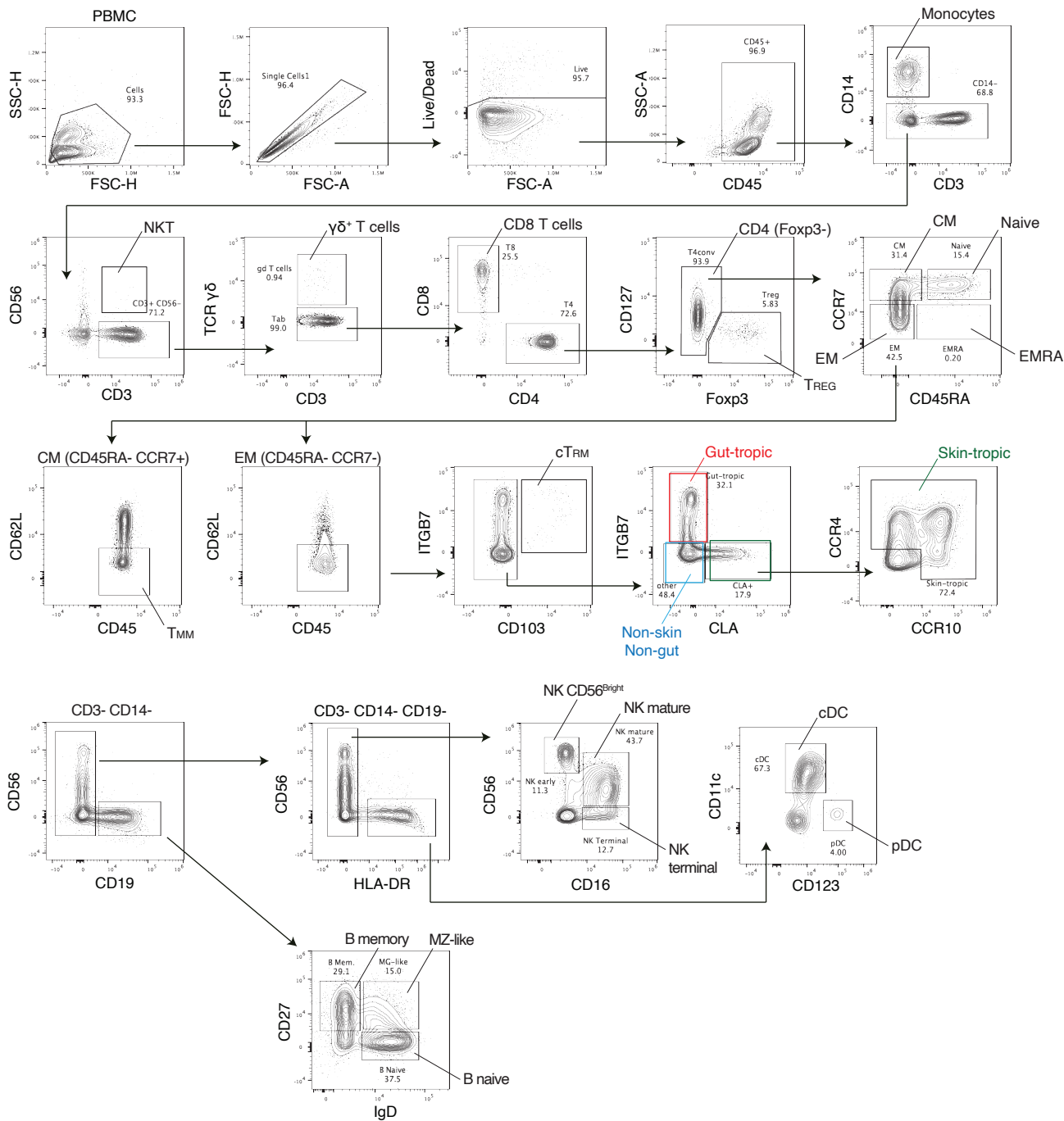

Supplement: Supplementary Data 6 [file NIHMS2157577-supplement-Supplementary_Data_6.pdf]

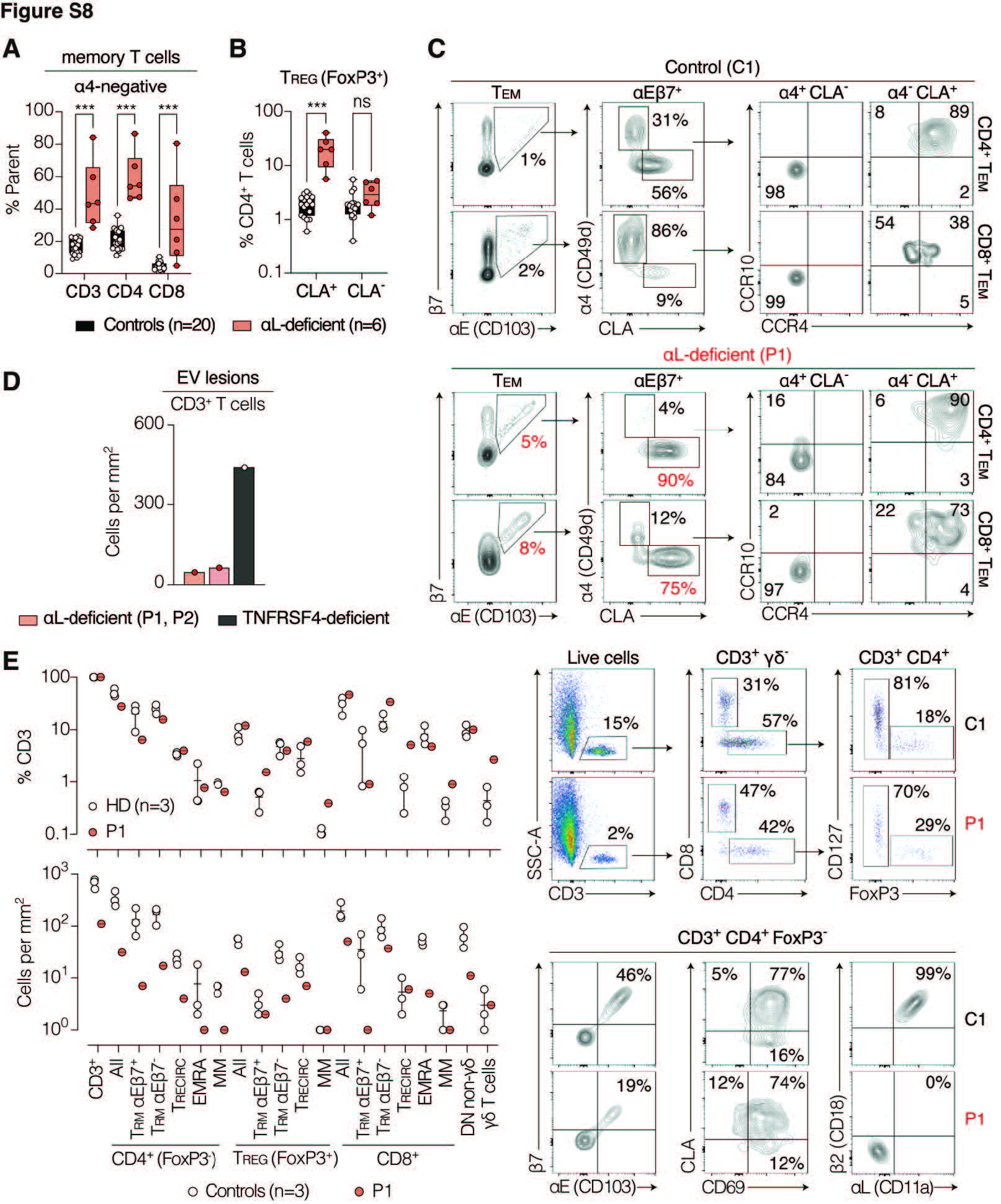

Supplement: Supplementary Fig 8 [file NIHMS2157577-supplement-Supplementary_Fig_8.jpeg]

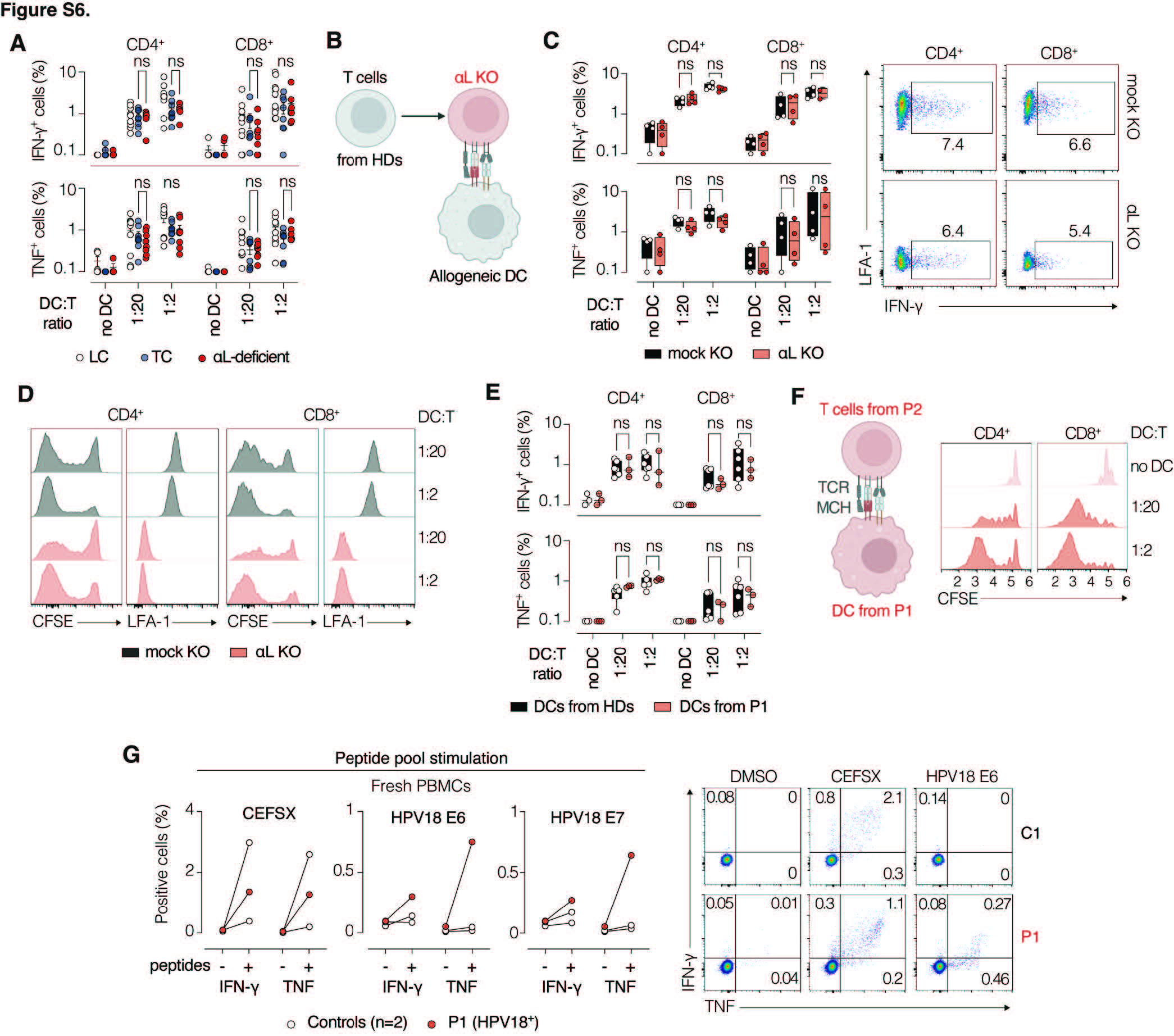

Supplement: Supplementary Fig 6 [file NIHMS2157577-supplement-Supplementary_Fig_6.jpeg]

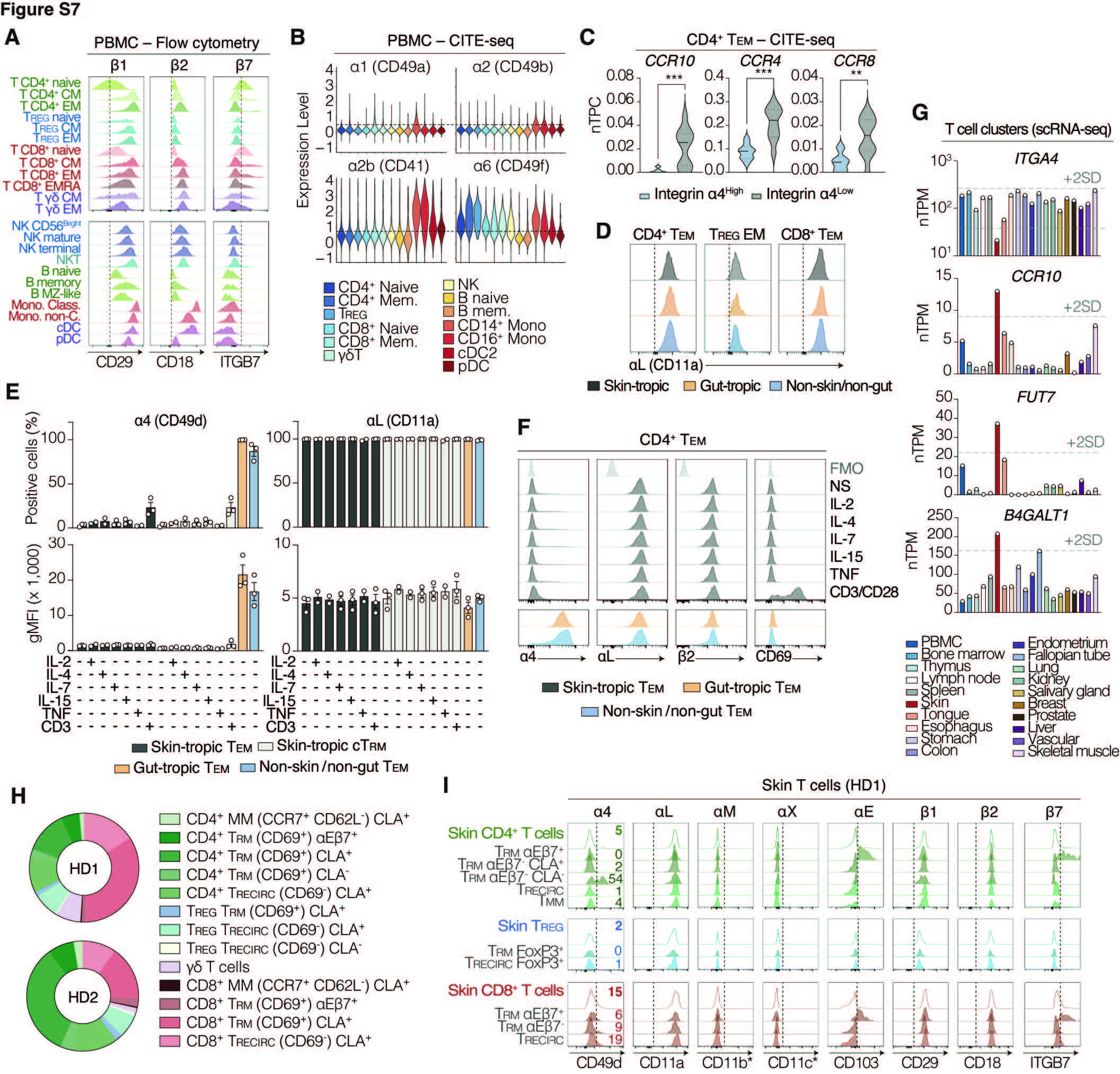

Supplement: Supplementary Fig 7 [file NIHMS2157577-supplement-Supplementary_Fig_7.jpeg]

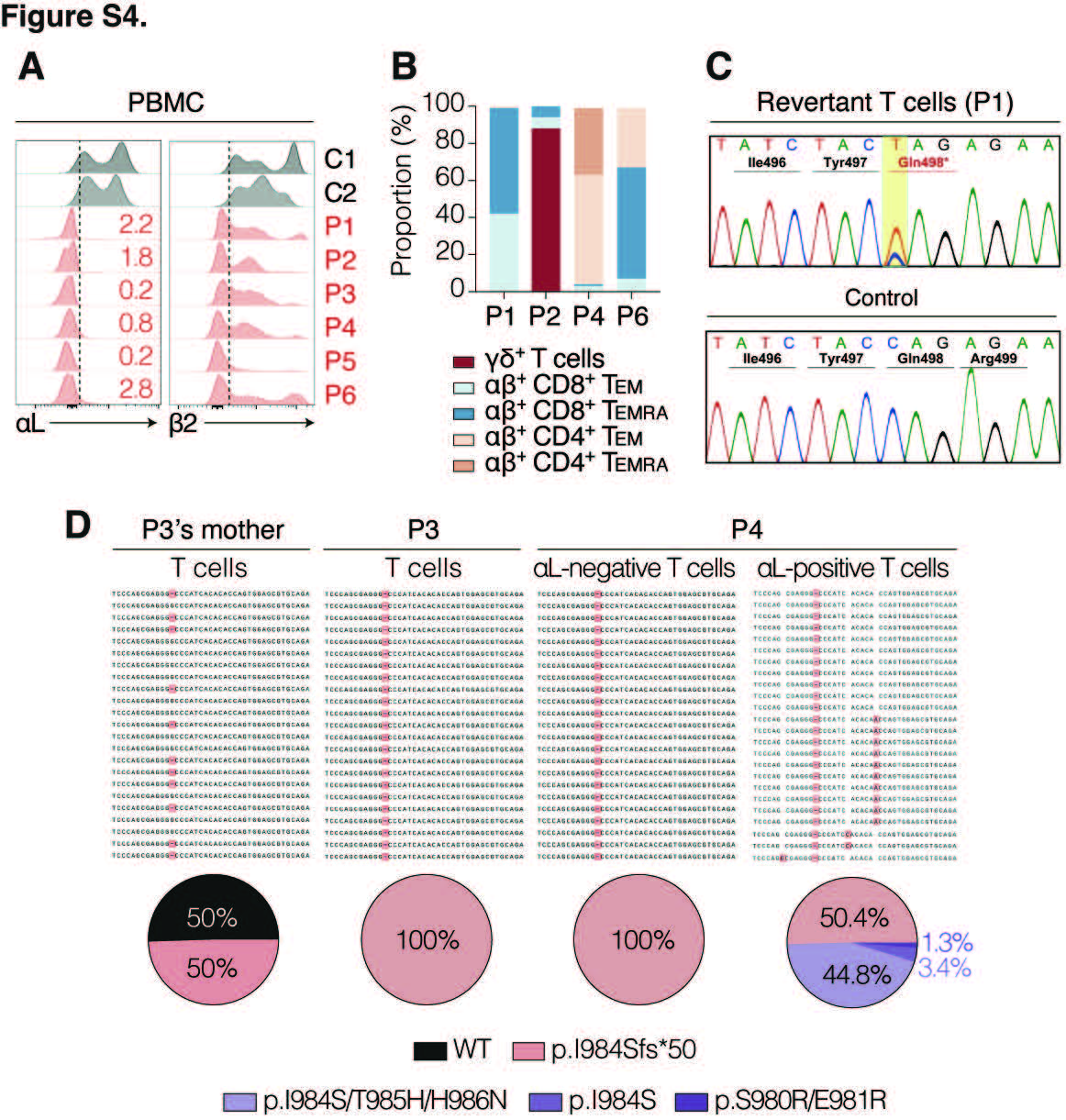

Supplement: Supplementary Fig 4 [file NIHMS2157577-supplement-Supplementary_Fig_4.jpeg]

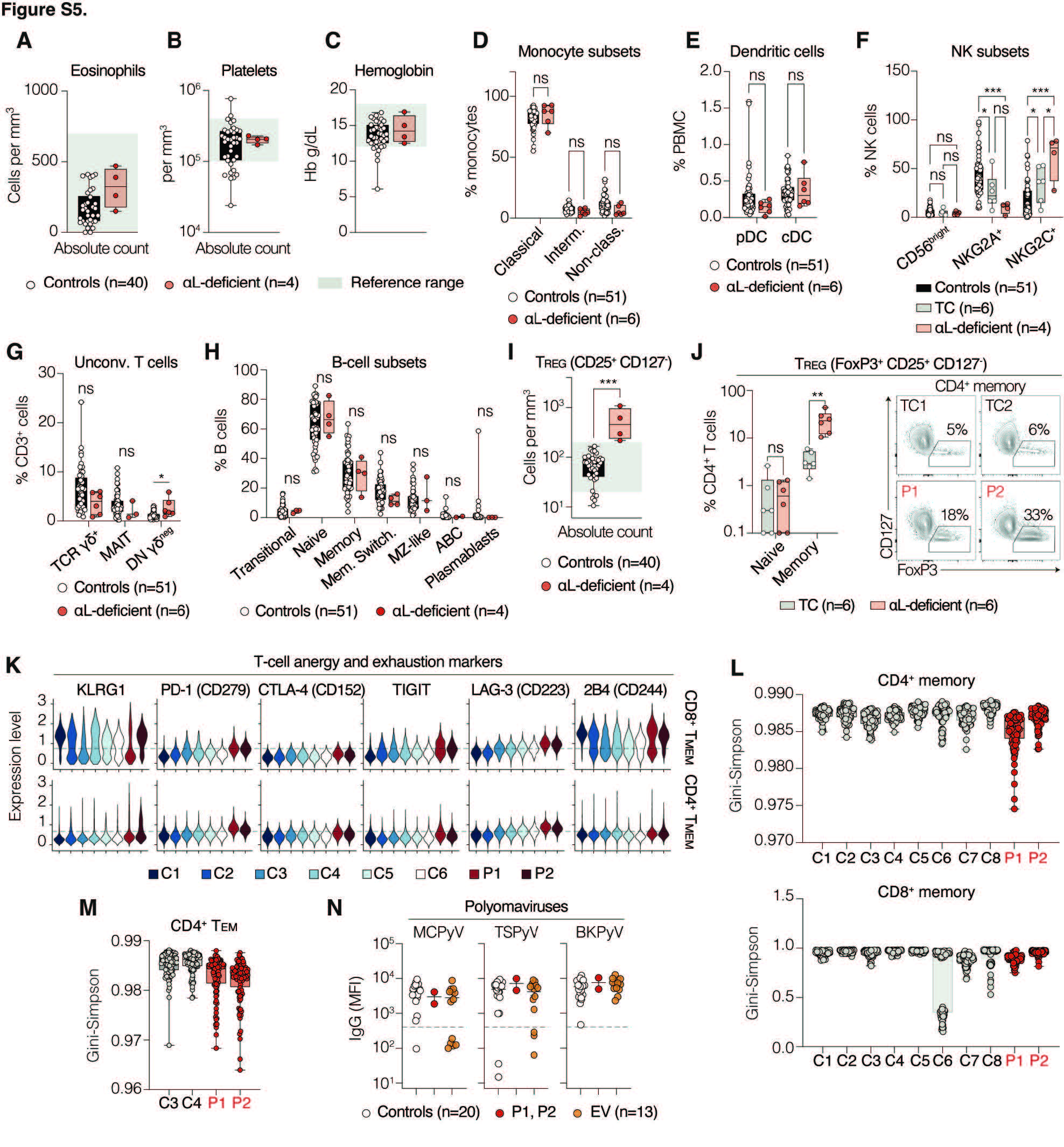

Supplement: Supplementary Fig 5 [file NIHMS2157577-supplement-Supplementary_Fig_5.jpeg]

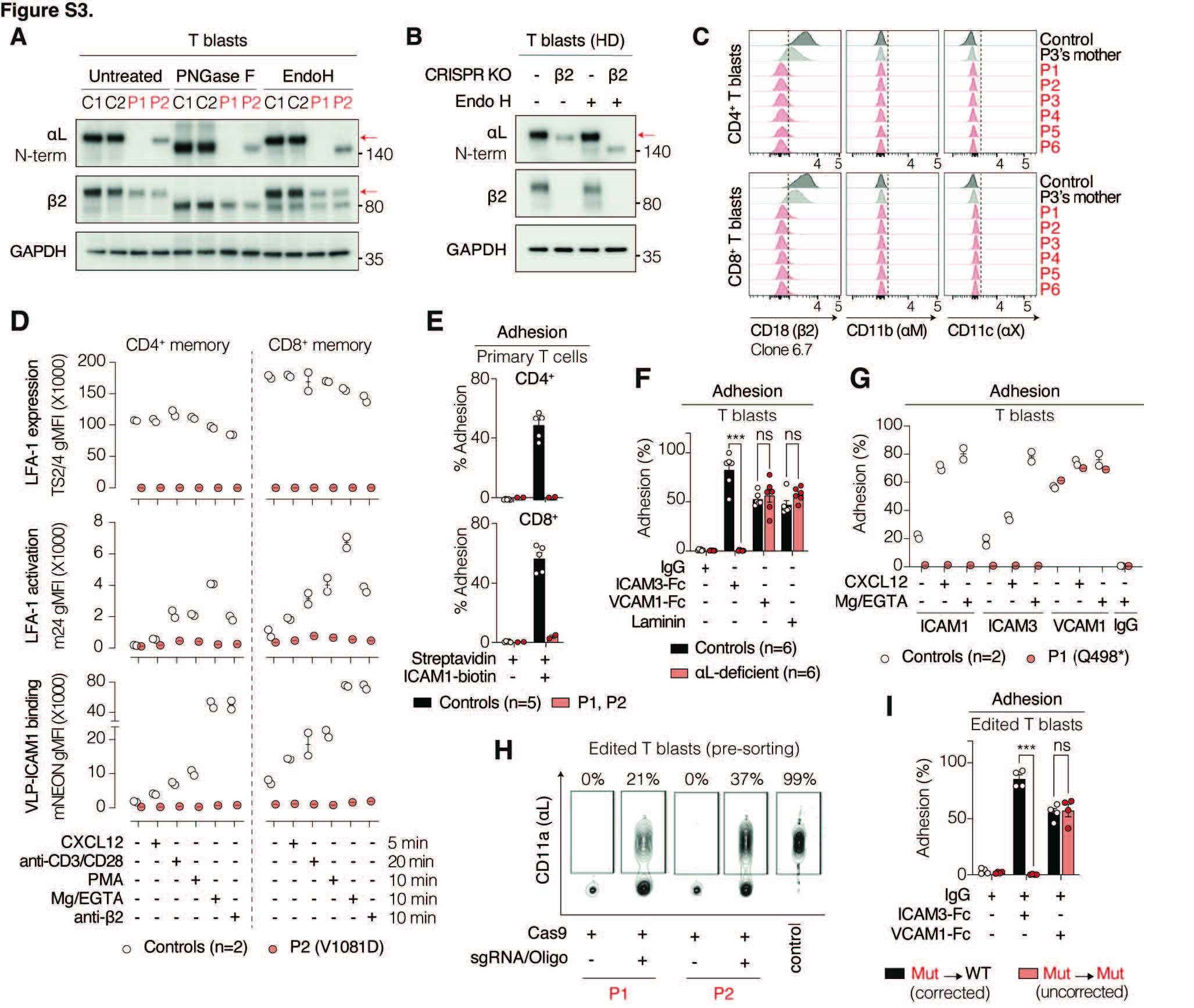

Supplement: Supplementary Fig 3 [file NIHMS2157577-supplement-Supplementary_Fig_3.jpeg]

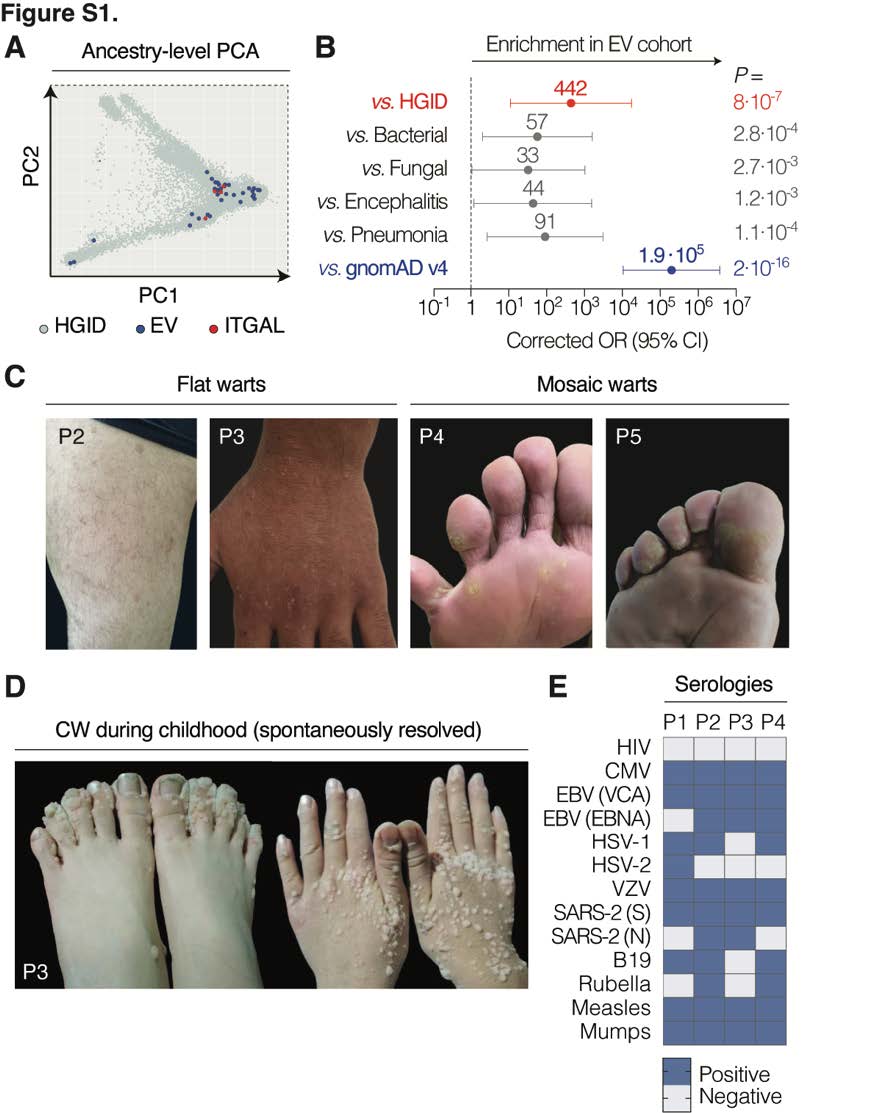

Supplement: Supplementary Fig 1 [file NIHMS2157577-supplement-Supplementary_Fig_1.jpeg]

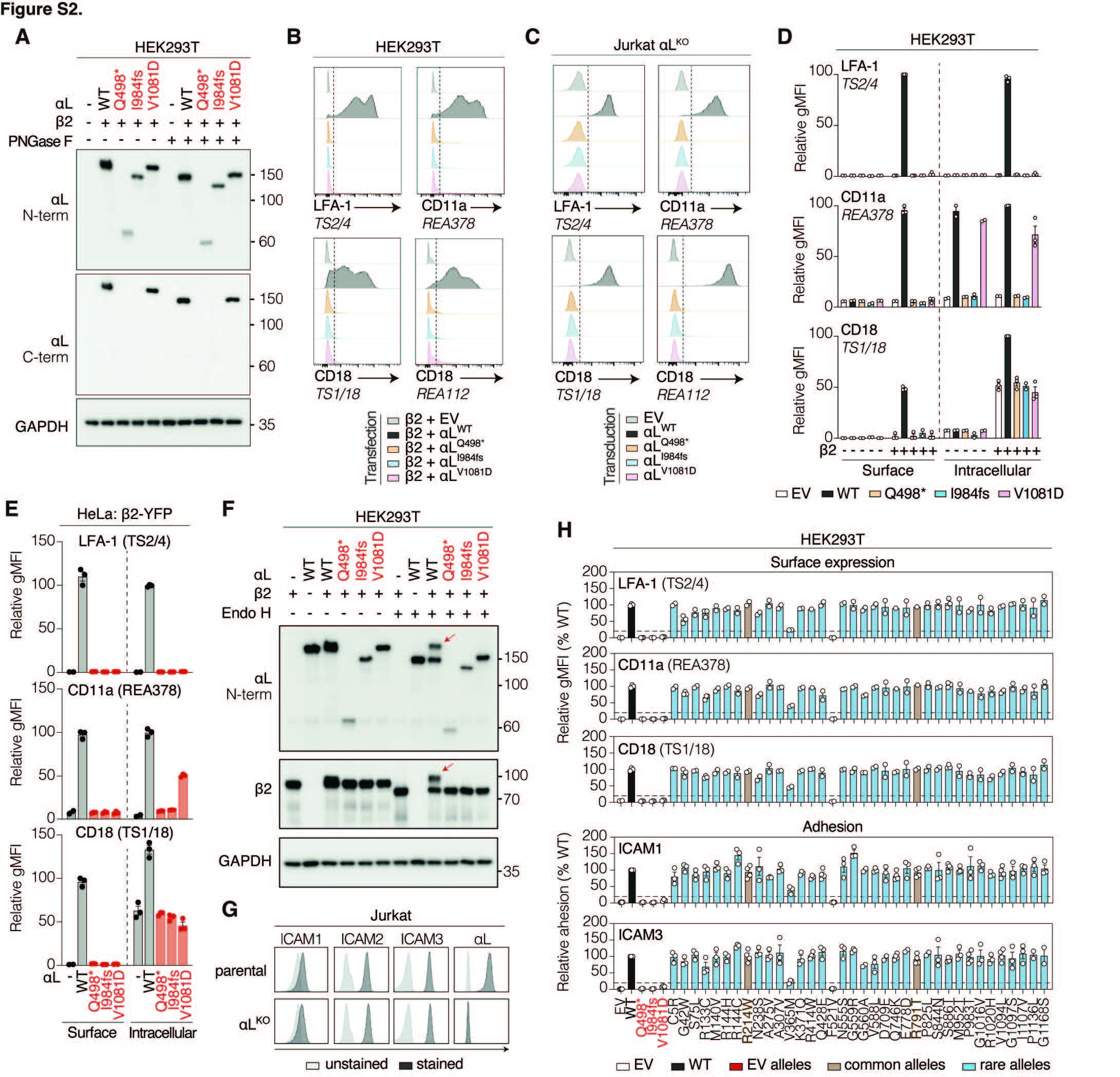

Supplement: Supplementary Fig 2 [file NIHMS2157577-supplement-Supplementary_Fig_2.jpeg]
