# Supplementary material for: Human LFA-1 governs T cell immune surveillance of the skin
Source: Sci Immunol. Author manuscript; Available in PMC 2026 May 13. (PMC13171165; doi:10.1126/sciimmunol.adz8360)
Supplement: Supplementary Data 5 [file NIHMS2157577-supplement-Supplementary_Data_5.pdf]

Figure 2C

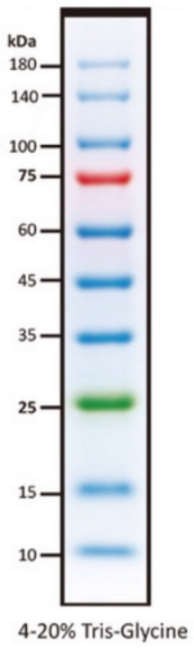
kDa
180
140
100
75
60
45
35
25
15
10
4-20% Tris-Glycine

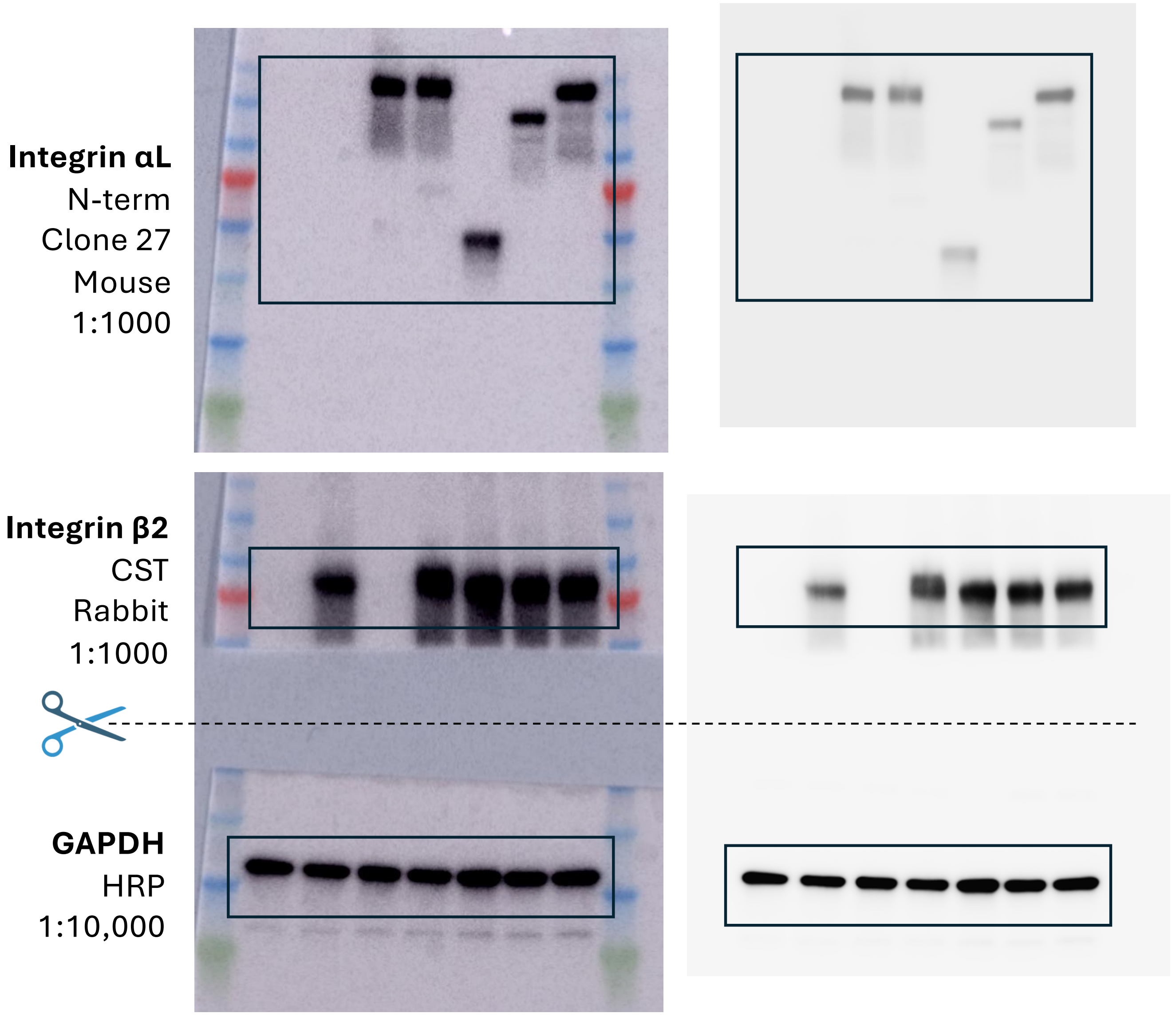
Integrin αL
N-term
Clone 27
Mouse
1:1000
Integrin β2
CST
Rabbit
1:1000
GAPDH
HRP
1:10,000

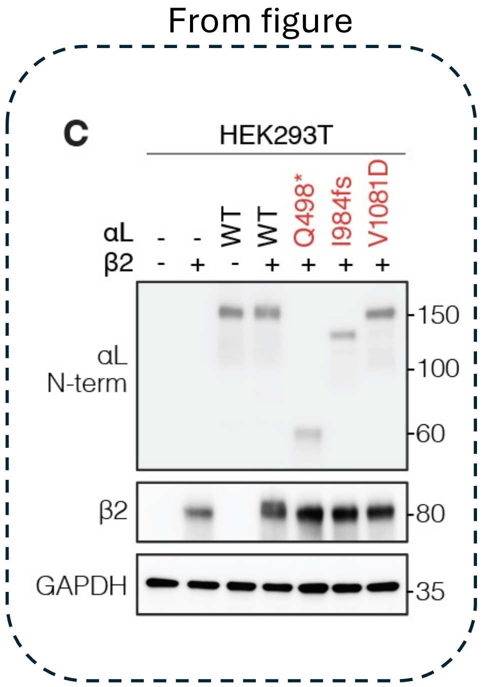
From figure
C
HEK293T
αL - - WT WT Q498* I984fs V1081D
β2 - + - + + + +
αL
N-term
150
100
60
β2
80
GAPDH
35

Figure 2G (1/2)

Integrin αL

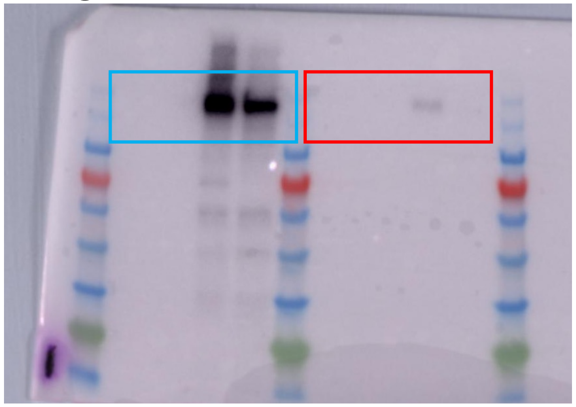

Integrin αL (long exposure)

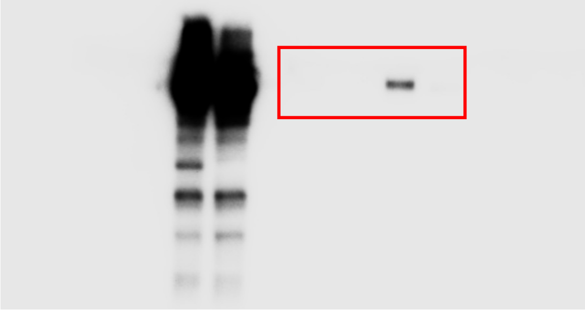

Integrin αL (short exposure)

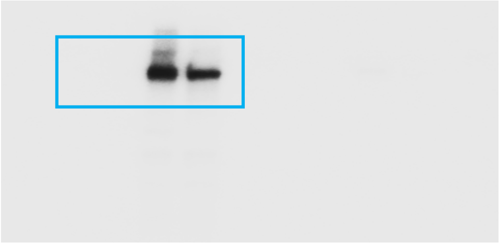

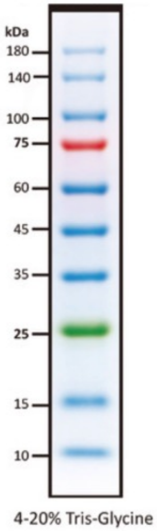

From figure

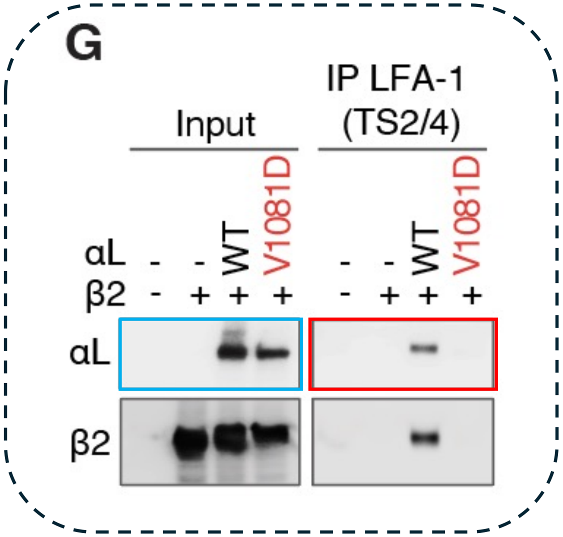

Figure 2G (2/2)

Integrin β2

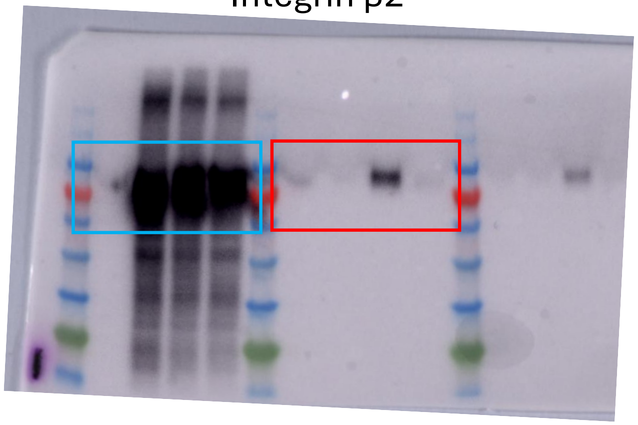

Integrin β2 (long exposure)

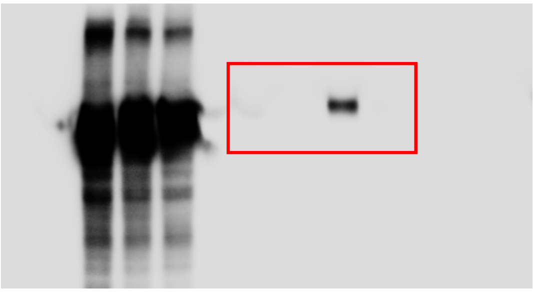

Integrin β2 (short exposure)

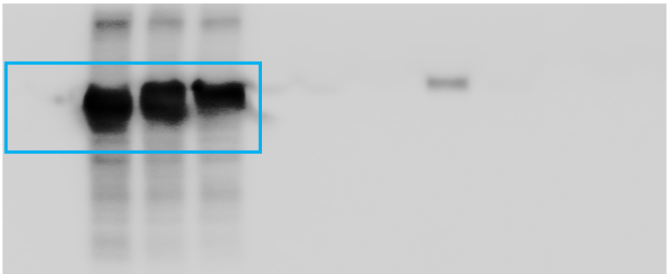

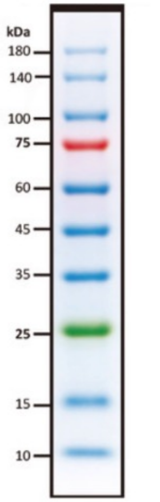

4-20% Tris-Glycine

From figure

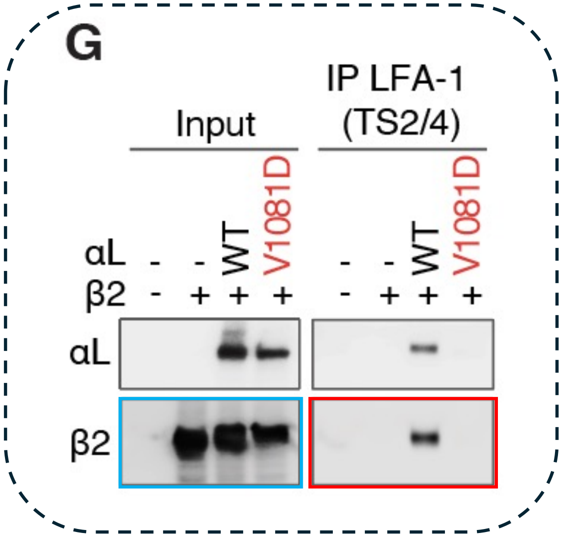

Figure 2H

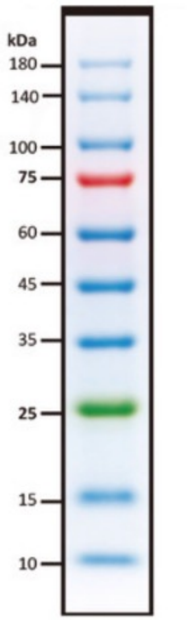

4-20% Tris-Glycine

HA (integrin β2)

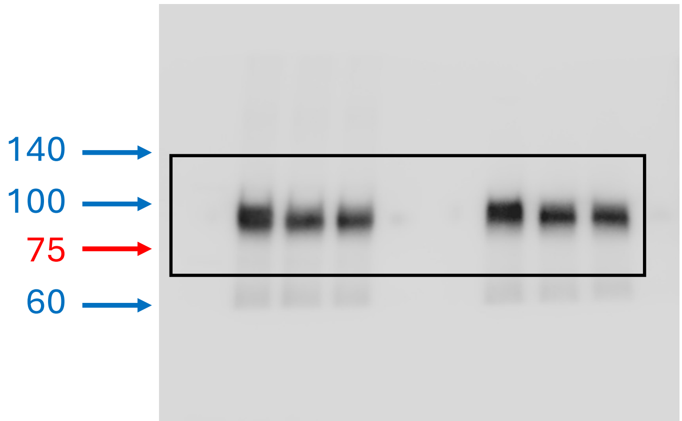

Rabbit mAb; 1:1000

Integrin αL

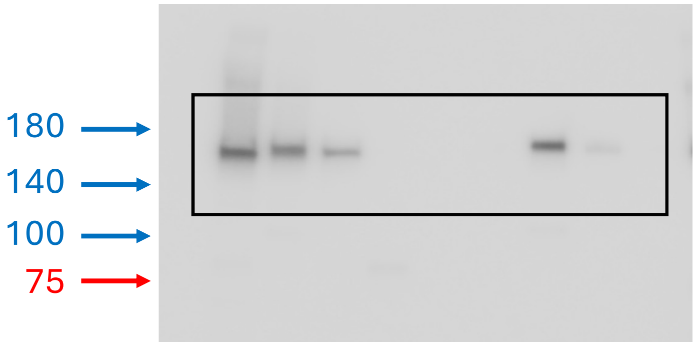

Mouse mAb; Clone 27; N-term; 1:1000

			vol uL	% of total
1	Ladder			
2	Input 1	ITGAL WT	10 + 5	~2%
3	Input 2	ITGB2-HA + ITGAL WT	10 + 5	~2%
4	Input 3	ITGB2-HA + ITGAL V1081D	10 + 5	~2%
5	Input 4	ITGB2-HA + ITGAL Q498*	10 + 5	~2%
6	Ladder			
7	HA IP 1	ITGAL WT	15	~20%
8	HA IP 2	ITGB2-HA + ITGAL WT	15	~20%
9	HA IP 3	ITGB2-HA + ITGAL V1081D	15	~20%
10	HA IP 4	ITGB2-HA + ITGAL Q498*	15	~20%

From figure

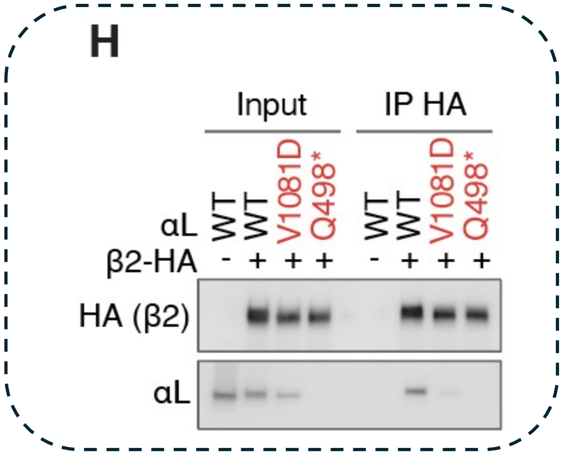

Figure 3A (1/2)

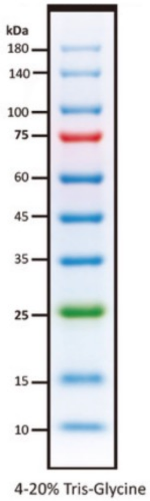

Integrin αL

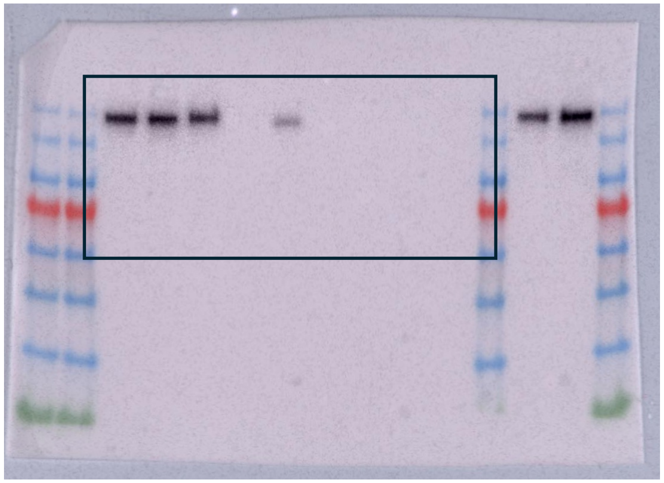

Integrin αL

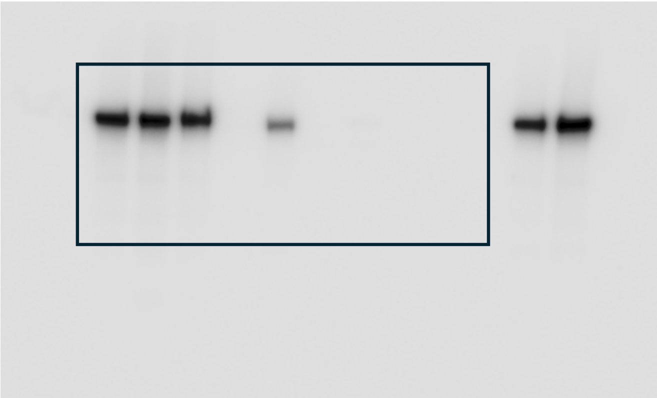

Integrin αL
N-term
Clone 27
Mouse
1:1000

Well				ul/well	ug/well
1	Ladder				
2	Ladder				
3	C1	TC2		15	10
4	C2	TC3		15	10
5	C3	LC		15	10
6	P1		Q498*	15	10
7	P2		V1081D	15	10
8	P3		I984fs	15	10
9	P4		I984fs	15	10
10	P5		Q498*	15	10
11	P6		Q498*	15	10
12	Ladder				
13	Mother (P3)		Het (Q498*)	15	10
14	Husband (P5)		Unknown	15	10
15	Ladder				

From figure

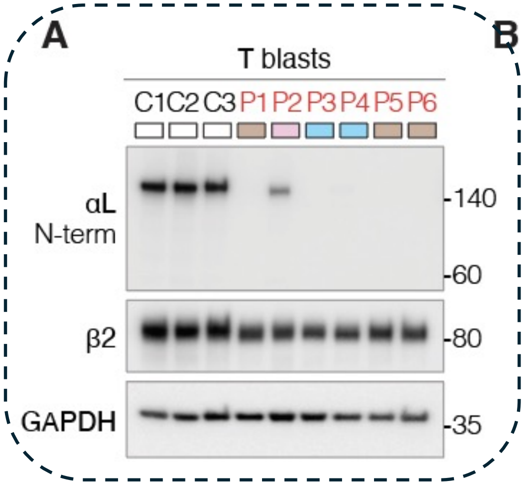

Figure 3A (2/2)

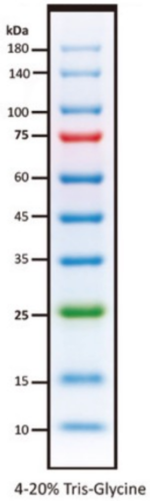

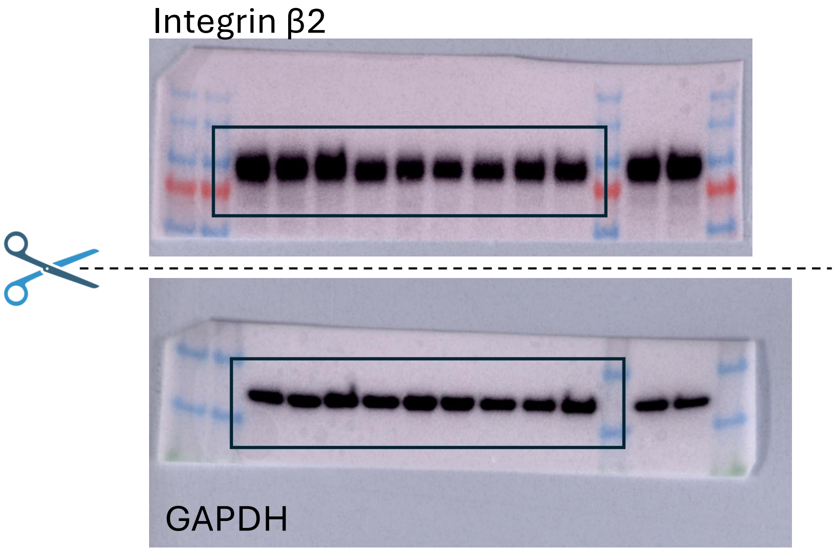

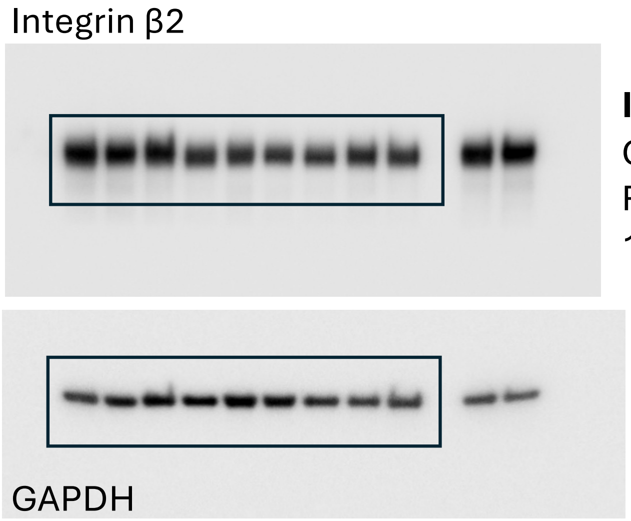

Integrin β2
CST
Rabbit
1:1000

GAPDH
HRP
1:10,000

Well				ul/well	ug/well
1	Ladder				
2	Ladder				
3	C1	TC2		15	10
4	C2	TC3		15	10
5	C3	LC		15	10
6	P1		Q498*	15	10
7	P2		V1081D	15	10
8	P3		I984fs	15	10
9	P4		I984fs	15	10
10	P5		Q498*	15	10
11	P6		Q498*	15	10
12	Ladder				
13	Mother (P3)		Het (Q498*)	15	10
14	Husband (P5)		Unknown	15	10
15	Ladder				

From figure

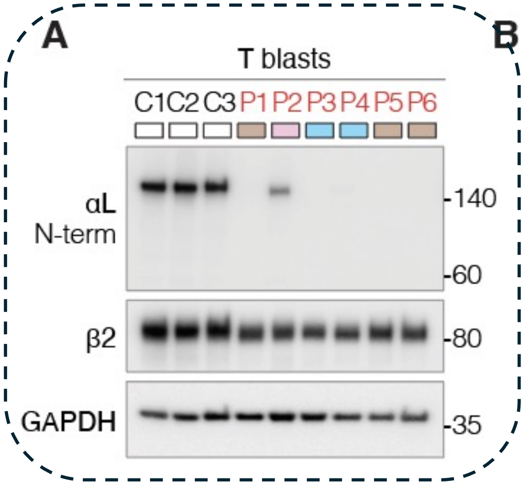

Figure S2A (1/2)

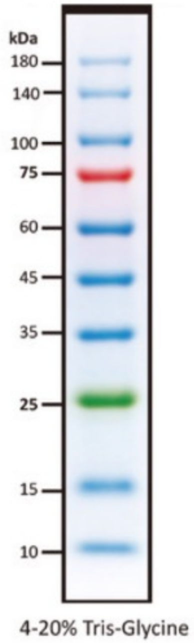

Integrin αL
N-term
Clone 27
Mouse
1:1000

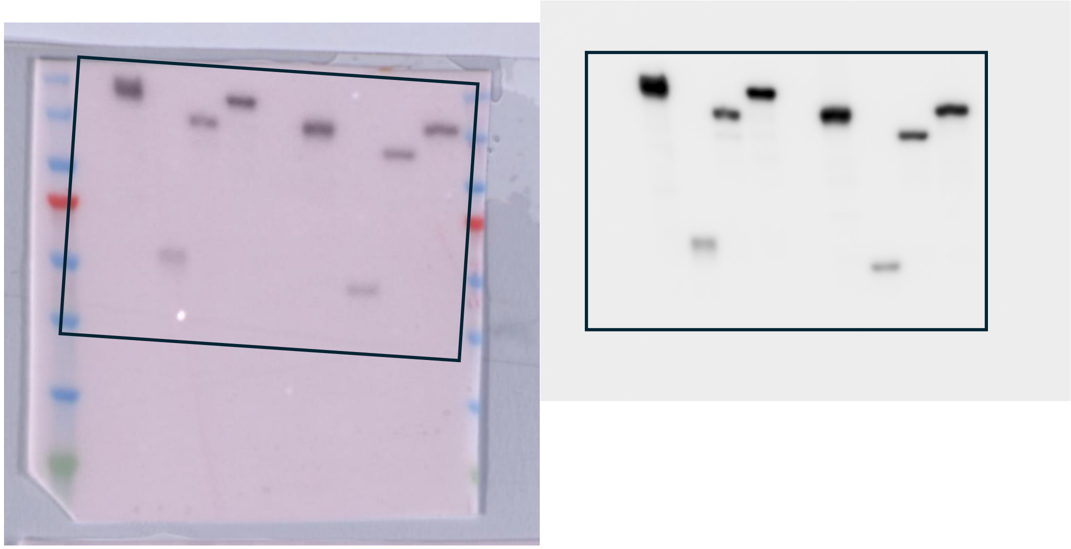

Integrin αL
C-term
CST
Rabbit
1:1000

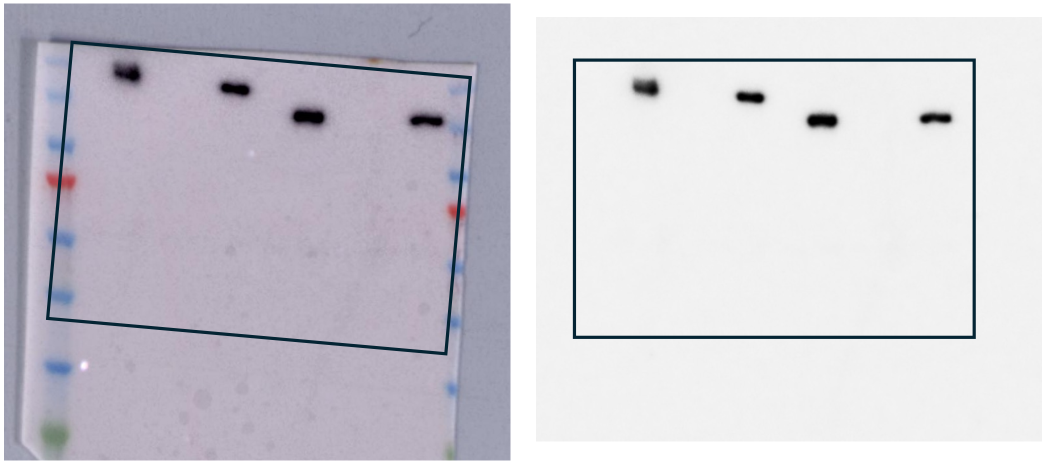

From figure

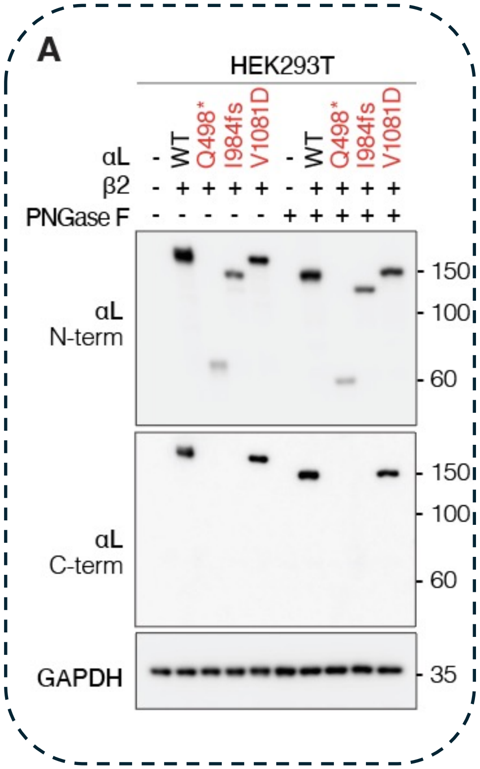

Figure S2A (2/2)

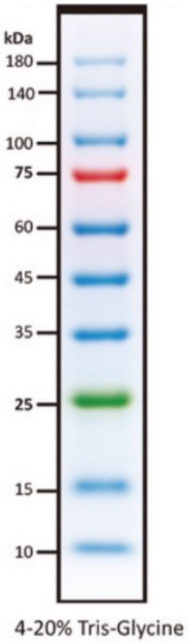

GAPDH
HRP
1:10,000

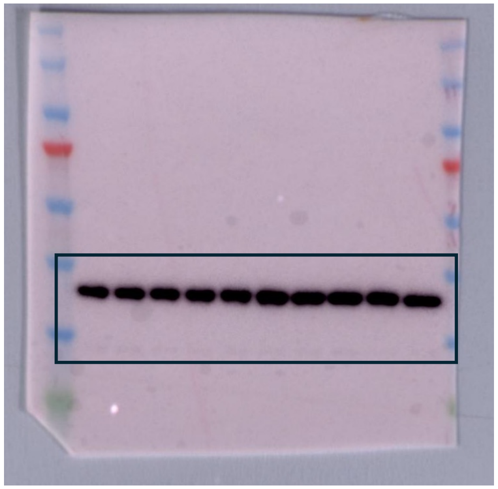

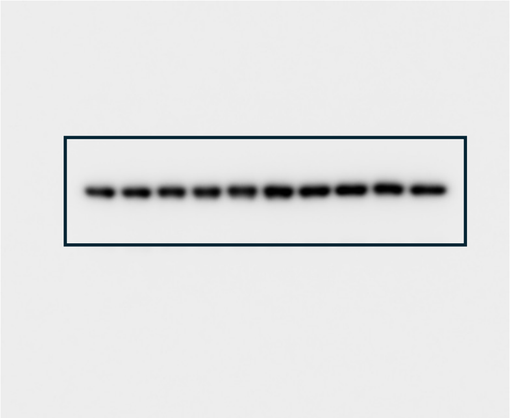

From figure

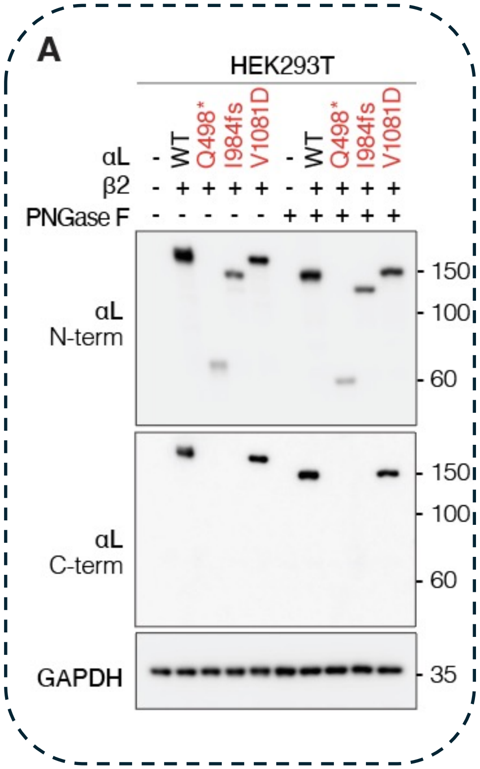

Figure S2F (1/2)

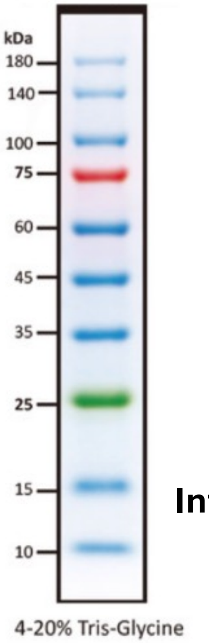

Integrin αL
N-term
Clone 27
Mouse
1:1000

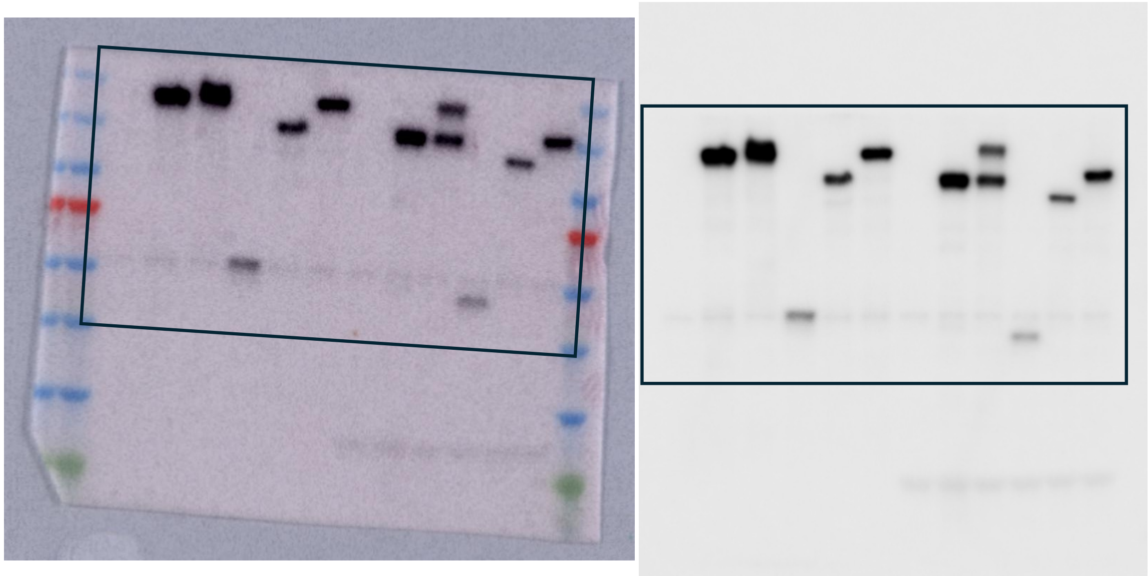

Integrin β2
CST
Rabbit
1:1000

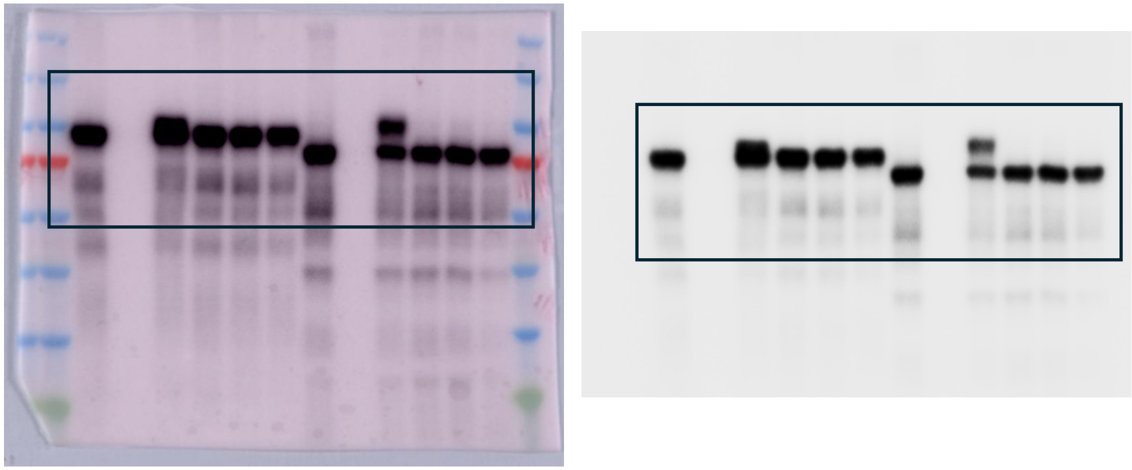

From figure

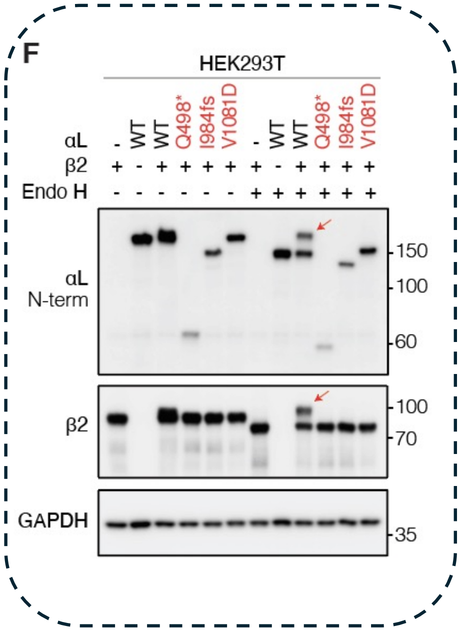

Figure S2F (2/2)

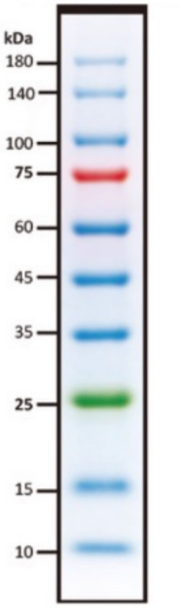

4-20% Tris-Glycine

GAPDH
HRP
1:10,000

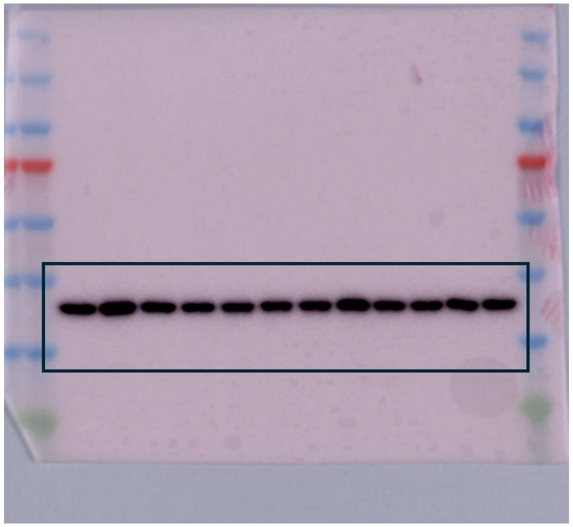

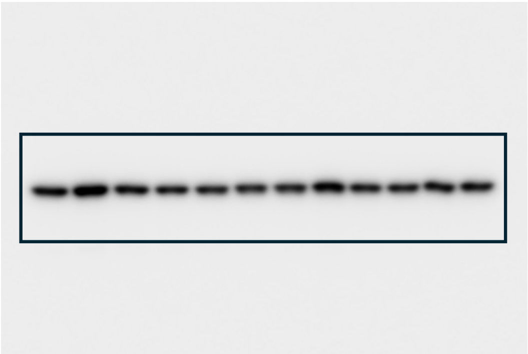

From figure

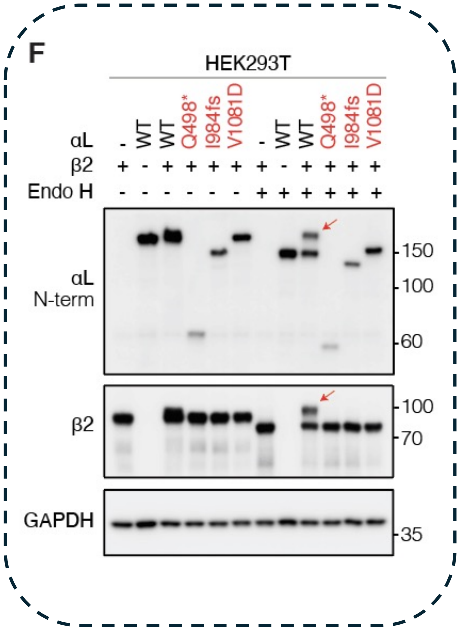

Figure S3A (1/2)

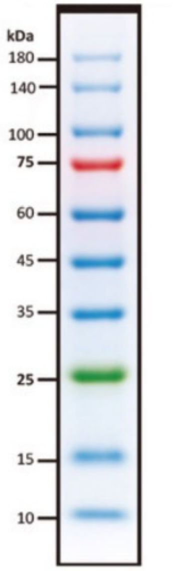

4-20% Tris-Glycine

Integrin αL

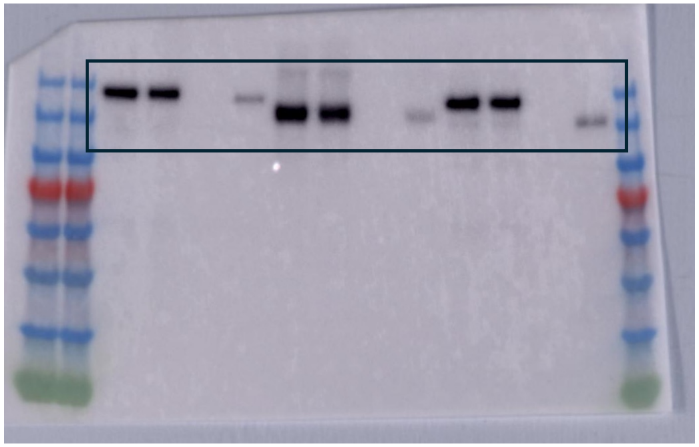

Integrin αL

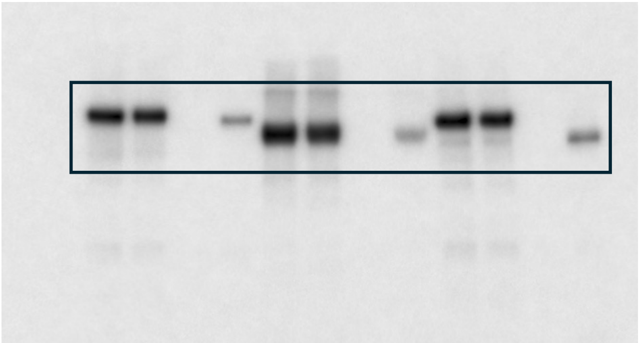

Well				ul/well	ug/well
1	Ladder				
2	Ladder				
3	D2	PHA-blast TC1	Undigested	12.5	5.3
4	D5	PHA-blast TC2	Undigested	12.5	5.0
5	D1	PHA-blast P1	Undigested	12.5	5.6
6	D4	PHA-blast P2	Undigested	12.5	5.7
7	D2	PHA-blast TC1	PNGase F	12.5	5.3
8	D5	PHA-blast TC2	PNGase F	12.5	5.0
9	D1	PHA-blast P1	PNGase F	12.5	5.6
10	D4	PHA-blast P2	PNGase F	12.5	5.7
11	D2	PHA-blast TC1	EndoH	12.5	5.3
12	D5	PHA-blast TC2	EndoH	12.5	5.0
13	D1	PHA-blast P1	EndoH	12.5	5.6
14	D4	PHA-blast P2	EndoH	12.5	5.7
15	Ladder				

From figure

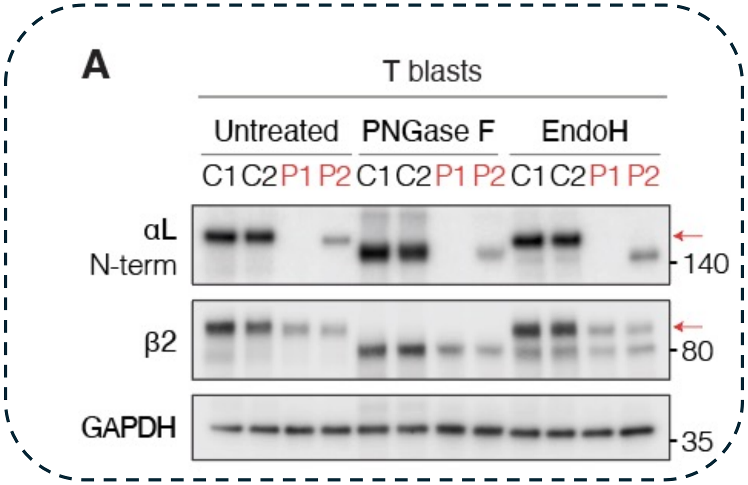

Figure S3A (2/2)

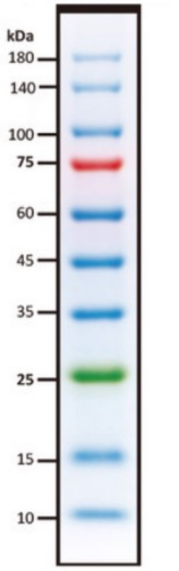

4-20% Tris-Glycine

Integrin β2

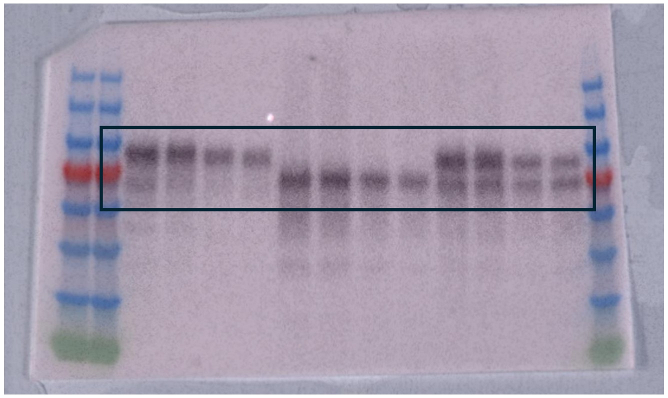

Integrin β2

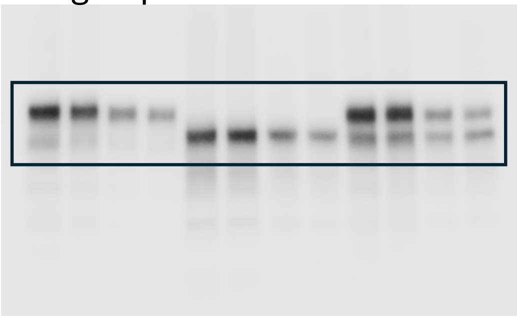

GAPDH

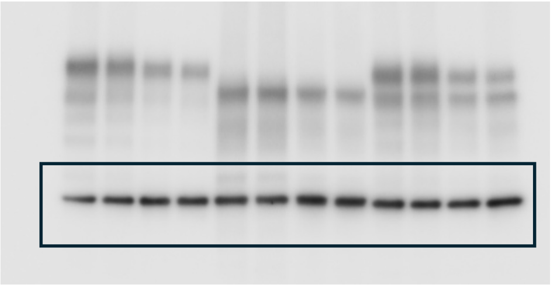

Well				ul/well	ug/well
1	Ladder				
2	Ladder				
3	D2	PHA-blast TC1	Undigested	12.5	5.3
4	D5	PHA-blast TC2	Undigested	12.5	5.0
5	D1	PHA-blast P1	Undigested	12.5	5.6
6	D4	PHA-blast P2	Undigested	12.5	5.7
7	D2	PHA-blast TC1	PNGase F	12.5	5.3
8	D5	PHA-blast TC2	PNGase F	12.5	5.0
9	D1	PHA-blast P1	PNGase F	12.5	5.6
10	D4	PHA-blast P2	PNGase F	12.5	5.7
11	D2	PHA-blast TC1	EndoH	12.5	5.3
12	D5	PHA-blast TC2	EndoH	12.5	5.0
13	D1	PHA-blast P1	EndoH	12.5	5.6
14	D4	PHA-blast P2	EndoH	12.5	5.7
15	Ladder				

From figure

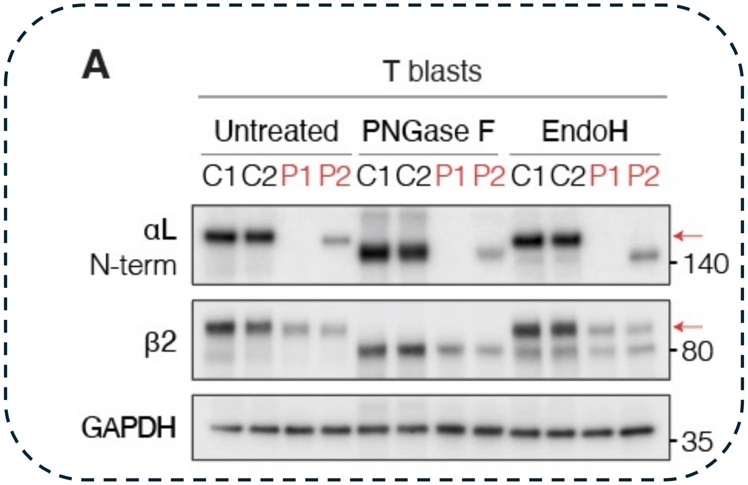

Figure S3B

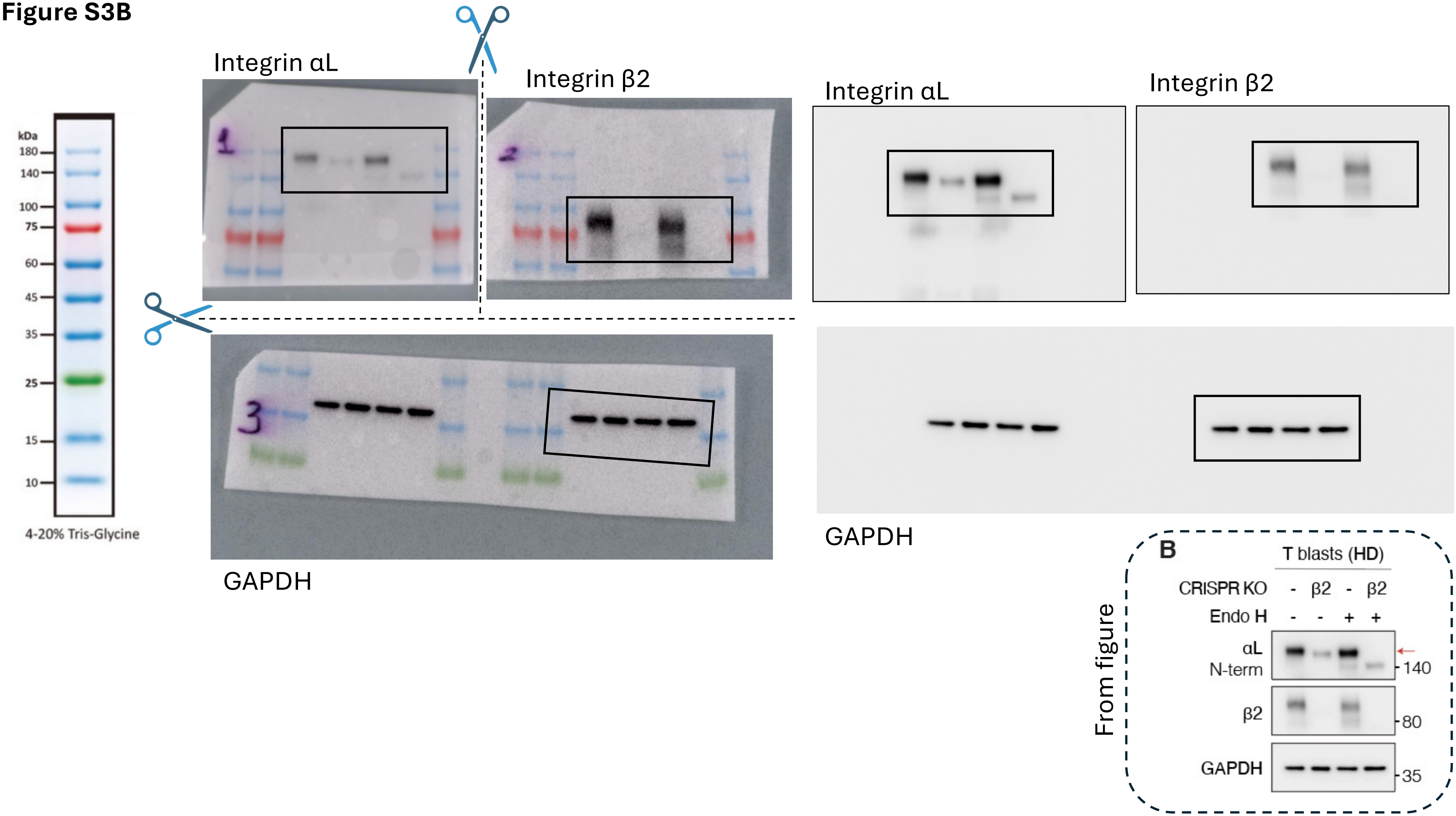
kDa
180
140
100
75
60
45
35
25
15
10
4-20% Tris-Glycine
Integrin αL
Integrin β2
1
2
3
GAPDH
Integrin αL
Integrin β2
GAPDH
From figure
B
T blasts (HD)
CRISPR KO - β2 - β2
Endo H - - + +
αL
N-term
140
β2
80
GAPDH
35
