# Supplementary material for: Human LFA-1 governs T cell immune surveillance of the skin
Source: Sci Immunol. Author manuscript; Available in PMC 2026 May 13. (PMC13171165; doi:10.1126/sciimmunol.adz8360)
Supplement: Supplementary Table 10 [file NIHMS2157577-supplement-Supplementary_Table_10.pdf]

Table S10. Custom-designed mass cytometry (CyTOF) panels for the deep immunophenotyping of whole-blood cells

Panel 1	Identifier	Panel 2	Identifier
CXCR3	RRID:AB_2810969	CCR4	RRID:AB_2074395
TCRgd	RRID:AB_2687643	CCR7	RRID:AB_2814291
CD19	RRID:AB_2651155	CD11c	RRID:AB_1236381
CD38	RRID:AB_2687640	CD123	RRID:AB_2661822
CD123	RRID:AB_2661794	CD127	RRID:AB_2661792
Va7.2	RRID:AB_2891190	CD14	RRID:AB_314192
CD3	RRID:AB_2811086	CD141	RRID:AB_2661788
CD45RA	RRID:AB_2810246	CD16	RRID:AB_314214
CD27	RRID:AB_2858231	CD161	RRID:AB_2661837
CD1c	RRID:AB_1088995	CD169	RRID:AB_2189031
CLEC9A	RRID:AB_2810252	CD19	RRID:AB_2661817
CD161	RRID:AB_2687651	CD1c	RRID:AB_2661820
CD8	RRID:AB_2892771	CD20	RRID:AB_314250
iNKT	RRID: AB_3678002	CD209	RRID:AB_1134253
CCR4	RRID:AB_2921320	CD24	RRID:AB_314851
CD4	RRID:AB_314069	CD25	RRID:AB_2661833
CD21	RRID:AB_2811646	CD27	RRID:AB_2661825
NKG2C	RRID:AB_2801859	CD3	RRID:AB_2661835
CD20	RRID:AB_314250	CD33	RRID:AB_314346
HLA-DR	RRID:AB_2810248	CD38	RRID:AB_2661836
CCR10	RRID:AB_2801898	CD4	RRID:AB_314070
CD45	RRID:AB_2938863	CD45	RRID:AB_2661851
CD66b	RRID:AB_2814367	CD45RA	RRID:AB_314406
CCR6	RRID:AB_2687639	CD56	RRID:AB_395903
CD127	RRID:AB_2810240	CD57	RRID:AB_2661815
CD11c	RRID:AB_2687850	CD66b	RRID:AB_2661823
CD25	RRID:AB_2756416	CD69	RRID:AB_314837
NKVFS1	RRID:AB_323743	CD8	RRID:AB_2661818
CCR7	RRID:AB_2858236	CD86	RRID:AB_314530
NKG2A	RRID:AB_2756426	CX3CR1	RRID:AB_1595422
CXCR5	RRID:AB_2858239	CXCR3	RRID:AB_2655743
CD24	RRID:AB_2938867	CXCR5	RRID:AB_2801905
CD31	RRID:AB_2737262	HLADR	RRID:AB_314680
CD14	RRID:AB_2687634	IgD	RRID:AB_10550095
CD56	RRID:AB_2938870	PD1	RRID:AB_11147365
CD57	RRID:AB_2562403	PDL1	RRID:AB_2275581
KIR3DL1L2	RRID:AB_2889458		
IgD	RRID:AB_2811082		
CD16	RRID:AB_2756431		
