# Supplementary material for: Human LFA-1 governs T cell immune surveillance of the skin
Source: Sci Immunol. Author manuscript; Available in PMC 2026 May 13. (PMC13171165; doi:10.1126/sciimmunol.adz8360)
Supplement: Supplementary Table 9 [file NIHMS2157577-supplement-Supplementary_Table_9.pdf]

Table S9. Serum levels of immunoglobulins

	P1	P2	P4	P5	Unit	Normal Ranges (Adult)
IgG	7.11	13.7	13.2	16.6	g/L	6-16 g/L
IgA	1.11	1.9	3.09	1.13	g/L	0.8-3 g/L
IgM	0.65	1.36	0.98	1.54	g/L	0.4-2.5 g/L

	P1	P2	P4	P5	Unit	Normal Ranges (Adult)
IgG1	3.96	6.6	6.92	N.A.	g/L	3.824-9.286 g/L
IgG2	1.37	5.47	4.15	N.A.	g/L	2.418-7.003 g/L
IgG3	0.417	<0.0547	0.67	N.A.	g/L	0.2182-1.761 g/L
IgG4	0.188	0.383	0.39	N.A.	g/L	0.039-0.864 g/L
