# Supplementary material for: Human LFA-1 governs T cell immune surveillance of the skin
Source: Sci Immunol. Author manuscript; Available in PMC 2026 May 13. (PMC13171165; doi:10.1126/sciimmunol.adz8360)
Supplement: Supplementary Table 11 [file NIHMS2157577-supplement-Supplementary_Table_11.pdf]

Table S11. Full-spectrum flow cytometry Homing panel

Channel	Specificity	Color	Clone	Vendor	Cat#	Staining	Dilution
UV2	CD45RA	BUV395	5H9	BD Biosciences	740315	Intracellular	12,000
UV6	CD45	AF350	2D1	R&D Systems	FAB1430U	Intracellular	200
UV7	HLA-DR	BUV496	L243	BD Biosciences	753685	Intracellular	5,000
UV9	CD11b (ITGAM)	BUV563	ICRF44	Thermo Fisher Scientific	365-0118-42	Intracellular	1,000
UV10	CCR4	BUV615	1G1	BD Biosciences	613000	Intracellular	100
UV11	TCRγδ	BUV661	11F2	BD Biosciences	750019	Surface	100
UV14	CD56	BUV737	TULY56	Thermo Fisher Scientific	367-0566-42	Intracellular	400
UV16	CD18	BUV805	6.7	BD Biosciences	749381	Surface	100
V3	CD11c (ITGAX)	eFluor 450	3.9	Thermo Fisher Scientific	48-0116-42	Intracellular	400
V5	IgD	BV480	IA6-2	Thermo Fisher Scientific	414-9868-41	Surface	200
V7	CD123	BV510	6H6	BioLegend	306022	Surface	800
V8	CD16	BV570	3G8	BioLegend	302036	Surface	200
V10	CD103 (ITGAE)	BV605	Ber-ACT8	BioLegend	350217	Surface	400
V11	CD28	BV650	CD28.2	Thermo Fisher Scientific	416-0289-42	Surface	100
V13	CCR6	BV711	G034E3	BioLegend	353436	Surface	100
V14	CCR7	BV750	G043H7	BioLegend	353253	Intracellular	400
V15	CD49d (ITGA4)	BV785	9F10	BioLegend	304344	Surface	200
B1	CCR10	BB515	1B5	Thermo Fisher Scientific	564769	Intracellular	400
B3	CD14	Spark Blue 550	63D3	BioLegend	367147	Intracellular	60,000
B5	CD8	NovaFluor Blue 585	OKT-8	Thermo Fisher Scientific	H003T02B04	Intracellular	200
B6	CD3	NovaFluor B610-70S	SK7	Thermo Fisher Scientific	H028T03B06	Intracellular	2000
B8	CD11a (ITGAL)	PerCP	TS2/4	BioLegend	350608	Surface	200
B10	OX40	PerCP-Vio700	REA621	Miltenyi Biotec	130-129-007	Surface	200
B14	FoxP3	RB780	236A/E7	BD Biosciences	569086	Intracellular	200
YG1	CLA	PE	HECA-452	Biolegend	321311	Surface	100
YG2	CD27	NovaFluor Y 590	O323	Thermo Fisher Scientific	H012T03Y02	Surface	100
YG3	CD29 (ITGB1)	PE-Dazzle594	TS2/16	BioLegend	303031	Surface	400
YG5	CD69	PE-Cy5	FN50	BioLegend	310908	Intracellular	1600
YG6	CXCR5	NovaFluor Y 690	MU5UBEE	Thermo Fisher Scientific	H037T03Y05	Surface	100
YG7	CD25	PE-Alexa Fluor700	CD25-3G10	Thermo Fisher Scientific	MHCD2524	Surface	200
YG8	CD4	cFluor BYG750	SK3	Cytek Biosciences	SKU R7-20160	Intracellular	6000
YG9	CD49c (ITGA3)	PE-vio770	REA360	Miltenyi Biotec	130-105-367	Surface	100
YG10	CXCR3	PE-Fire810	G025H7	BioLegend	353760	Surface	100
R1	ITGB7	APC	FIB504	BioLegend	321207	Surface	400
R4	CD127	NovaFluor Red 710	eBioRDR5	Thermo Fisher Scientific	H017T03R04	Surface	100
R5	Viability	ViaKrome 808	-	Beckman Coulter	C36628	-	2,000
R7	Integrin $\alpha 9 \beta 1$	APC-vio770	REA483	Miltenyi Biotec	130-129-859	Surface	100
R8	CD19	APC-Fire810	HIB19	BioLegend	302272	Surface	200
