# Supplementary material for: Human LFA-1 governs T cell immune surveillance of the skin
Source: Sci Immunol. Author manuscript; Available in PMC 2026 May 13. (PMC13171165; doi:10.1126/sciimmunol.adz8360)
Supplement: Supplementary Table 8 [file NIHMS2157577-supplement-Supplementary_Table_8.pdf]

Table S8. Complete blood cell counts

Parameters	Unit	P1	P3	P4	P5	Mother (P3)	Sister (P3)	TC3	Controls (n=40)					Reference Interval
									Mean	Median	*SD*	Min.	Max.	
Age, Sex		67 yo, f	14 yo, m	56 yo, m	35 yo, f	45 yo, f	19 yo, f	32 yo, m	14-70 yo, f (19) / m (17) / N.A (4)					
WBC Count	10*3/µL	7.7	10.49	14.02	10.91	10.7	17.89	9.49	5.94	5.80	1.22	4.08	9.96	4.0 - 13.5
Hemoglobin	g/dL	12.5	16.9	15	13.4	15.3	14.5	12	13.6	13.7	2.0	6.1	16.8	13 - 18
Platelet Count	10*3/µL	208	230	205	172	309	352	183	205	203	142	24	770	150 - 450
Neutrophils Absolute	10*3/µL	3.56	5.28	8.38	5.15	6.43	15.07	4.16	3.27	3.21	0.97	1.53	6.61	1.8 - 7.7
Lymphocytes Absolute	10*3/µL	3.46	3.99	4.58	4.48	3.07	1.52	4.49	2.06	2.05	0.50	1.20	3.40	1 - 4.8
Monocytes Absolute	10*3/µL	0.42	0.82	0.79	0.8	1.09	1.27	0.75	0.45	0.41	0.18	0.16	0.87	0.1 - 1.4
Eosinophils Absolute	10*3/µL	0.15	0.39	0.26	0.47	0.1	0.02	0.08	0.18	0.16	0.13	0.00	0.41	0 - 0.7
Basophils Absolute	10*3/µL	0.04	0.01	0.01	0.01	0.01	0.01	0.01	0.04	0.04	0.03	0.00	0.11	0 - 0.3
Neutrophils Relative	%	46.5	50.4	59.7	47.2	60.1	84.2	43.9	N.A	N.A	N.A	N.A	N.A	40.0 - 73.0 %
Lymphocytes Relative	%	45.2	38	32.7	41.1	28.7	8.5	47.3	N.A	N.A	N.A	N.A	N.A	20.0 - 44.0 %
Monocytes Relative	%	5.5	7.8	5.6	7.3	10.2	7.1	7.9	N.A	N.A	N.A	N.A	N.A	3.0 - 13.0 %
Eosinophils Relative	%	2	3.7	1.9	4.3	0.9	0.1	0.8	N.A	N.A	N.A	N.A	N.A	0.0 - 6.0 %
Basophils Relative	%	0.5	0.1	0.1	0.1	0.1	0.1	0.1	N.A	N.A	N.A	N.A	N.A	0.0 - 3.0 %
RBC Count	10*6/µL	4.15	6.17	5.42	4.81	5.66	5.35	6.24	N.A	N.A	N.A	N.A	N.A	4.7 - 6.1
Hematocrit	%	38.2	46.8	43.7	41.7	44.2	41.8	36.4	N.A	N.A	N.A	N.A	N.A	41 - 52
MCV	fL	92	75.9	80.6	86.7	78.1	78.1	58.3	N.A	N.A	N.A	N.A	N.A	80 - 100
MCH	pg	30.1	27.4	27.7	27.9	27	27.1	19.2	N.A	N.A	N.A	N.A	N.A	28-32
MCHC	g/dL	32.7	36.1	34.3	32.1	34.6	34.7	33	N.A	N.A	N.A	N.A	N.A	30-37
RBC Distribution Width	%	12.7	13.7	13.1	13.1	13.5	13.4	18.1	N.A	N.A	N.A	N.A	N.A	11 - 14.5

TC3, Travel control of P3; f, female; m, male; yo, years old; WBC, white blood cell; RBC, red blood cell; MCV, mean corpuscular volume; MCH, mean corpuscular hemoglobin; MCHC, mean corpuscular hemoglobin concentration; N.A, not available.
