# Supplementary material for: Human LFA-1 governs T cell immune surveillance of the skin
Source: Sci Immunol. Author manuscript; Available in PMC 2026 May 13. (PMC13171165; doi:10.1126/sciimmunol.adz8360)
Supplement: Supplementary Table 7 [file NIHMS2157577-supplement-Supplementary_Table_7.pdf]

Table S7. *ITGAL* variants found in the homozygous state in the general population.

Variant CHR-POS.-REF-ALT	AF	rsIDs	Protein consequence	Abbreviated notation	*In vitro* Activity (this study)	CADD	SpliceAI	AlphaMissense	Revel max	Number of homozygous individuals: All	GnomAD non-UKBB	UKBB	All of Us	BRAVO	G_Asia 100K	Turkish Variome	Iranome	GME
16-30472850-T-C	7.30E-04	rs374629566	p.Cys5Arg	C5R	Neutral	7.62	0	0.0854	0.228	13	12	0	0	0	1	0	0	0
16-30474258-G-T	6.86E-06	rs1006417970	p.Gly42Trp	G42W	Neutral	25.3	0.01	0.4044	0.661	1	1	0	0	0	0	0	0	0
16-30475365-C-T	4.09E-05	rs774130388	p.Ser75Leu	S75L	Neutral	11.3	0.01	0.072	0.071	1	1	0	0	0	0	0	0	0
16-30479160-C-T	4.40E-05	rs768038639	p.Arg133Cys	R133C	Neutral	16.1	0.01	0.1145	0.269	1	1	0	0	0	0	0	0	0
16-30479181-A-G	1.19E-03	rs577741062	p.Met140Val	M140V	Neutral	0.924	0.01	0.0458	0.032	28	19	7	1	0	0	0	1	0
16-30479193-C-T	2.11E-05	rs146416180	Arg144Cys	R144C	Neutral	18.4	0	0.141	0.118	1	0	0	0	1	0	0	0	0
16-30479194-G-A	1.32E-03	rs34166708	p.Arg144His	R144H	Neutral	0.12	0	0.0626	0.026	97	21	10	39	27	0	0	0	0
16-30481502-C-T	4.29E-02	rs1064524	p.Arg214Trp	R214W	Neutral	0.36	0	0.0987	0.23	2301	735	1063	320	182	0	0	1	0
16-30481575-A-G	1.74E-05	rs140551792	Asn238Ser	N238S	Neutral	18.8	0.08	0.091	0.189	1	0	0	0	1	0	0	0	0
16-30483928-C-T	4.52E-05	rs147749266	p.Ala275Val	A275V	Neutral	0.009	0	0.0805	0.097	1	1	0	0	0	0	0	0	0
16-30484177-C-T	5.82E-05	rs764595721	Ala307Val	A307V	Neutral	0.012	0	0.077	0.01	1	0	0	1	0	0	0	0	0
16-30489266-G-A	2.29E-05	rs760763568	Val365Met	V365M	Hypomorphic	25.8	0	0.201	0.28	1	0	0	1	0	0	0	0	0
16-30489284-A-C	7.69E-05	rs2230431	p.Lys371Gln	K371Q	Neutral	18.9	0	0.1144	0.161	3	2	0	0	1	0	0	0	0
16-30494238-C-T	5.21E-05	rs551557846	p.Arg414Trp	R414W	Neutral	20	0.01	0.0849	0.245	2	2	0	0	0	0	0	0	0
16-30494280-C-G	5.95E-05	rs200737227	p.Gln428Glu	Q428E	Neutral	22.2	0	0.0831	0.094	2	1	0	0	1	0	0	0	0
16-30496154-T-G	1.24E-06	rs1382792976	Phe521Val	F521V	LOF	25.9	0	0.826	0.535	1	0	0	0	1	0	0	0	0
16-30496257-A-G	5.73E-05	rs369827114	p.Asn555Ser	N555S	Neutral	22.4	0.04	0.1084	0.159	1	1	0	0	0	0	0	0	0
16-30496268-G-A	3.62E-05	rs751825408	p.Gly559Arg	G559R	Neutral	13.1	0.01	0.109	0.2	2	0	0	0	0	0	1	1	0
16-30496272-G-C	1.25E-06	rs767738577	p.Gly560Ala	G560A	Neutral	22.8	0	0.098	0.373	1	0	0	1	0	0		0	0
16-30496496-G-T	6.07E-05	rs376988959	p.Val588Leu	V588L	Neutral	22.6	0	0.1547	0.182	2	1	0	0	1	0	0	0	0
16-30499471-C-A	1.08E-04	rs369990476	p.Asp709Glu	D709E	Neutral	0.195	0.02	0.0931	0.022	1	0	1	0	0	0	0	0	0
16-30505244-C-A	6.00E-03	rs34838942	p.Gln746Lys	Q746K	Neutral	8.44	0	0.0748	0.024	1939	413	130	774	620	2	0	0	0
16-30505430-G-C	7.93E-05	rs200200372	p.Glu778Asp	E778D	Neutral	22.7	0	0.3442	0.288	1	1	0	0	0	0	0	0	0
16-30506720-G-C	3.11E-01	rs2230433	p.Arg791Thr	R791T	Neutral	5.86	0.06	0.0735	0.03	129480	46,203	39,992	25,793	16,464	456	271	129	172
16-30506822-C-T	1.54E-04	rs143575422	p.Pro825Leu	P825L	Neutral	0.001	0	0.0765	0.027	3	0	1	1	0	0	1	0	0
16-30510383-G-A	3.04E-05	rs369314558	Ser844Asn	S844N	Neutral	19.4	0	0.182	0.157	1	0	0	0	1	0	0	0	0
16-30510918-G-C	1.43E-05	rs1436811391	p.Ser886Thr	S886T	Neutral	21.5	0.04	0.0985	0.168	1	1	0	0	0	0	0	0	0
16-30513839-T-C	3.23E-05	rs533945018	p.Met952Thr	M952T	Neutral	14.7	0	0.0836	0.078	2	0	2	0	0	0	0	0	0
16-30517057-C-A	2.48E-06	rs1435466084	p.Pro983Thr	P983T	Neutral	12.4	0	0.0911	0.116	1	1	0	0	0	0	0	0	0
16-30517810-G-T	3.18E-06	NA	p.Gly1016Val	G1016V	Neutral	22.4	0.68	0.1433	0.186	1	1	0	0	0	0	0	0	0
16-30517822-G-A	1.58E-03	rs59353760	p.Arg1020His	R1020H	Neutral	13.6	0.26	0.0591	0.06	113	29	6	47	30	0	0	0	1
16-30519908-G-T	2.55E-04	rs11574949	p.Val1094Leu	V1094L	Neutral	13.4	0	0.1666	0.051	4	2	0	2	0	0	0	0	0
16-30519917-G-A	1.25E-04	rs151035888	Gly1097Ser	G1097S	Neutral	3.2	0	0.068	0.072	2	0	0	1	1	0	0	0	0
16-30519947-A-G	6.16E-06	rs748315049	p.Ile1107Val	I1107V	Neutral	24.8	0	0.175	0.246	1	1	0	0	0	0	0	0	0
16-30521559-C-T	6.44E-05	rs548488325	p.Pro1136Leu	P1136L	Neutral	19.4	0	0.0671	0.096	1	1	0	0	0	0	0	0	0
16-30521654-G-A	2.48E-06	rs2051256244	p.Gly1168Ser	G1168S	Neutral	4.3	0	0.073	0.026	1	0	1	0	0	0	0	0	0
