# Supplementary material for: Human LFA-1 governs T cell immune surveillance of the skin
Source: Sci Immunol. Author manuscript; Available in PMC 2026 May 13. (PMC13171165; doi:10.1126/sciimmunol.adz8360)
Supplement: Supplementary Table 6 [file NIHMS2157577-supplement-Supplementary_Table_6.pdf]

Table S6. Gene constraint metrics for human *ITGAL* and other integrin genes

Gene	Chr.	GDI	pLI	LOEUF	CoNeS	f parameter	LoFTool	evoTol	RVIS	DOMINO	p(HI)	SCoNeS
Genes encoding LFA-1 subunits (Human)												
ITGAL	16	8.16	4.32E-03	0.27435	-0.79714517	0.33497779	0.374	35.7803791	-1.5180166	0.13486792	0.247	0.854
ITGB2	21	2.32	5.08E-15	0.72237	-0.47764708	0.32940298	0.0333	1.5886288	-0.511736	0.12159138	NA	0.88
Genes encoding other integrins (Human)												
ITGAM	16	7.35	3.31E-11	0.46286	-0.12103762	0.52748627	0.543	26.3043479	-0.4451645	0.40704113	NA	0.844
ITGAX	16	12.29	1.37E-15	0.54379	0.119558104	0.58270635	0.441	32.9431439	-0.4987378	0.27325842	NA	0.896
ITGAD	16	3.2	2.00E-21	0.67156	0.829684722	0.54654948	0.604	33.6789298	1.5130545	0.0981115	NA	0.926
ITGAV	2	4.32	2.28E-04	0.30201	-0.71312088	0.42633873	0.178	9.5261985	-0.685526	0.97782909	0.716	0.818
ITGAE	17	9.02	3.92E-20	0.679	0.745326624	0.57321175	0.49	28.8963211	0.64098247	0.07384187	0.194	0.984
ITGA1	5	11.45	2.61E-16	0.55945	0.113569843	0.6537382	0.416	26.8673356	-0.9011283	0.44737602	0.546	0.736
ITGA2	5	6.78	6.28E-11	0.43536	-0.13551415	0.47003772	0.501	30.9308808	-0.6177997	0.44612349	0.401	0.332
ITGA2B	17	12.19	1.52E-16	0.5723	-0.06736435	0.52219263	0.5	28.439242	-1.214143	0.34683684	0.486	0.924
ITGA3	17	7.09	3.55E-07	0.37383	0.095566961	0.46918378	0.713	85.6800446	-0.0896586	0.32095709	0.289	0.94
ITGA4	2	3.87	1.01E-04	0.31966	-0.41559834	0.43487763	0.697	85.4793757	-1.4446373	0.73925489	0.448	0.728
ITGA5	12	3.58	4.25E-01	0.22429	-0.93492025	0.37640357	NA	NA	-0.9490702	0.99936522	0.489	0.342
ITGA6	2	10.98	1.90E-07	0.37345	-0.18072421	0.48734491	0.608	42.1237459	-1.3852841	0.55744221	0.613	0.858
ITGA7	12	4.93	4.27E-17	0.60485	1.16136281	0.55511617	0.935	97.2240803	0.76495769	0.19850099	NA	0.81
ITGA8	10	4.97	6.08E-13	0.49183	0.201485651	0.51722952	NA	NA	-0.0435823	0.16041349	0.297	0.96
ITGA9	3	3.68	8.16E-03	0.27345	-0.09824009	0.42408145	0.555	88.4559644	-0.5276914	0.52254645	0.258	0.894
ITGA10	1	7.64	2.45E-19	0.60509	0.988383162	0.74981281	0.788	54.1750279	0.51770864	0.07612867	0.565	0.898
ITGA11	15	9.33	8.66E-08	0.38665	0.546441084	0.41713607	NA	79.2140469	1.21558737	0.24491572	0.265	0.81
ITGB1	10	0.55	9.82E-01	0.15499	-1.79887037	0.29795465	0.0195	0.9197325	-0.682579	0.99560292	NA	0.046
ITGB3	17	2.84	1.72E-03	0.32046	-0.485972	0.44574795	0.127	4.2363434	-0.179046	0.82848782	0.618	0.872
ITGB4	17	4.32	9.53E-19	0.50549	-0.24557332	0.51913547	0.0863	3.3500558	0.48688817	0.28131346	NA	0.87
ITGB5	3	7.87	9.89E-08	0.47626	-0.25118329	0.42131454	0.0949	4.7770346	0.78230167	0.4148376	0.555	0.436
ITGB6	2	3.41	1.63E-22	0.93071	-0.22663481	0.36265237	0.223	7.0791528	0.70360961	0.7617566	0.57	0.324
ITGB7	12	2.81	2.37E-06	0.42708	-0.81911636	0.37893334	0.143	5.942029	-0.5053156	0.24299112	0.215	0.838
ITGB8	7	2.73	9.98E-01	0.12564	-1.50190131	0.39815949	0.0237	7.6421405	-0.544885	0.93942698	0.47	0.14

NA, not available

Score	Name	Reference
GDI	Gene Damage Index	Itan Y et al., Proc Natl Acad Sci U S A. 2015. PMID: 26483451.
pLI	Probability of Loss-of-Function Intolerance	Lek M et al., Nature. 2016. PMID: 27535533.
LOEUF	Loss-of-Function Observed/Expected Upper Fraction	Karczewski et al., Nature. 2020. PMID: 32461654.
CoNeS	Consensus Negative Selection	Rapaport F et al., Proc Natl Acad Sci U S A. 2021. PMID: 33408250.
f parameter	f parameter from Selection Inference Using a Poisson Random Effects (SnIPRE	Eilertson et al., PLoS Comput Biol. 2012. PMID: 23236270
LoFTool	Gene intolerance score based on loss-of-function variants	Fadista J et al., Bioinformatics. 2017. PMID: 27563026.
evoTol	Protein-sequence based evolutionary intolerance	Rackham OJ et al., Nucleic Acids Res. 2015. PMID: 25550428.
RVIS	Residual Variation Intolerance Score	Petrovski S et al., PLoS Genet. 2013. PMID: 23990802.
DOMINO	Dominant Mode of Inheritance Score	Quinodoz M et al., Am J Hum Genet. 2017. PMID: 28985496.
p(HI)	Probability of Haploinsufficiency	Huang N et al., PLoS Genet. 2010. PMID: 20976243.
SCoNeS	supervised CoNeS: probability of being AR	Rapaport F et al., Proc Natl Acad Sci U S A. 2021. PMID: 33408250.
