# Supplementary material for: Human LFA-1 governs T cell immune surveillance of the skin
Source: Sci Immunol. Author manuscript; Available in PMC 2026 May 13. (PMC13171165; doi:10.1126/sciimmunol.adz8360)
Supplement: Supplementary Table 5 [file NIHMS2157577-supplement-Supplementary_Table_5.pdf]

Table S5. Serological results for antibodies against common viruses

Parameters	P1<br>67 years old	P2<br>33 years old	P3<br>14 years old	P4<br>56 years old	Threshold
IgG anti-HIV	0.09 (negative)	0.12 (negative)	0.24 (negative)	0.23 (negative)	
IgG anti-CMV	101 (positive)	142 (positive)	>180 (positive)	149 (positive)	>14
IgG anti-EBV (VCA)	>750 (positive)	>750 (positive)	>750 (positive)	>750 (positive)	>20
IgG anti-EBV (EBNA)	<3 (negative)	254 (positive)	60.9 (positive)	222 (positive)	>20
IgG anti-HSV-1	56.4 (positive)	53.8 (positive)	0.235 (negative)	56.1 (positive)	>1.1
IgG anti-HSV-2	3.05 (positive)	<0.500 (negative)	<0.50 (negative)	0.819 (negative)	>1.1
IgG anti-VZV	2018 (positive)	495.4 (positive)	2553 (positive)	2070 (positive)	>165
IgG anti-SARS-CoV-2 (Spike)	>2080 (positive)	>2080 (positive)	321 (positive)	339 (positive)	>33
IgG anti-SARS-CoV-2 (Nucleocapsid)	0.3 (negative)	9.62 (positive)	1.03 (positive)	0.07 (negative)	
IgG anti-rubella	<5 (negative)	98.4 (positive)	<5 (negative)	119 (positive)	>11
IgG anti-measles	>300 (positive)	267 (positive)	106 (positive)	>300 (positive)	>16.5
IgG anti-mumps	18.9 (positive)	132 (positive)	41.7 (positive)	>300 (positive)	>11
IgG anti-parvovirus B19	>150 (positive)	13.8 (positive)	<0.100 (negative)	15.7 (positive)	>1.1
