# Supplementary material for: Human LFA-1 governs T cell immune surveillance of the skin
Source: Sci Immunol. Author manuscript; Available in PMC 2026 May 13. (PMC13171165; doi:10.1126/sciimmunol.adz8360)
Supplement: Supplementary Table 4 [file NIHMS2157577-supplement-Supplementary_Table_4.pdf]

Table S4. HPV genotyping in skin biopsy specimens

Patients	Skin sample	β-HPV	γ-HPV	α-HPV
P1	Flat wart	HPV 5	-	-
P1	Flat wart	HPV 5	-	-
P1	Flat wart	HPV 5; 206	-	-
P2	Flat wart	HPV 24	-	HPV 3
P3	Flat wart	-	-	-
P4	Flat wart	HPV 17	HPV 119; 131; 175	-
P5	Flat wart	HPV 5; 8; 23; 38	-	-
P6	Flat wart	HPV 5; 8; 23; 24; 38; 99	-	-
P6	Flat wart	HPV 8; 23; 38; 99	-	-
P3	Common wart	-	-	HPV 2
P4	Common wart	HPV 151	HPV 129; 162; 175; 200; 201	HPV 2
P5	Common wart	HPV 5; 8; 22; 23; 38	HPV 169	-
P5	Normal skin	HPV 5; 23; 38	-	-
P6	Normal skin	HPV 5; 8; 23; 24; 38	-	HPV 57
		86% (12/14)	21% (3/14)	27% (4/14)
