# Supplementary material for: Human LFA-1 governs T cell immune surveillance of the skin
Source: Sci Immunol. Author manuscript; Available in PMC 2026 May 13. (PMC13171165; doi:10.1126/sciimmunol.adz8360)
Supplement: Supplementary Table 3 [file NIHMS2157577-supplement-Supplementary_Table_3.pdf]

Table S3. Summary of *ITGAL* variants identified in EV patients

**Patient(s)**	**P1, P5, P6**	**P2**	**P3, P4**
Variant (GRCh38)	16-30494839-C-T	16-30519870-T-A	16-30517055-GG-G
Chromosome	16	16	16
Position	30494839	30519870	30517055
Reference	C	T	GG
Alternative	T	A	G
rsIDs	NA	NA	rs2051177449
Allele Frequency*	0	0	0
Gene	*ITGAL*	*ITGAL*	*ITGAL*
Transcript (MANE)	NM_002209.3	NM_002209.3	NM_002209.3
Ensembl (MANE)	ENST00000356798.11	ENST00000356798.11	ENST00000356798.11
Exon	13/31	30/31	26/31
cDNA position	1588	3338	3042
CDS position	1492	3242	2946
Protein position	498	1081	982
VEP consequence	stop_gained	missense_variant	frameshift_variant
Protein consequence	p.Gln498*	p.Val1081Asp	p.Ile984Serfs*50
Abbreviated notation	**Q498***	**V1081D**	**I984fs**
CADD v1.7	36	22.5	22.8
AlphaMissense	NA	0.9309	NA
SIFT	NA	deleterious(0)	NA
PolyPhen	NA	NA	NA
LoGo Neutral	NA	0.466142898	NA
LoGo GOF	NA	0.418948547	NA
LoGo LOF	NA	0.114908555	NA
SpliceAI	0.04	0.02	0

NA, not available; *, from gnomAD v4.1
