# Supplementary material for: Human LFA-1 governs T cell immune surveillance of the skin
Source: Sci Immunol. Author manuscript; Available in PMC 2026 May 13. (PMC13171165; doi:10.1126/sciimmunol.adz8360)
Supplement: Supplementary Table 2 [file NIHMS2157577-supplement-Supplementary_Table_2.pdf]

Table S2. Enrichment of the EV cohort in biallelic *ITGAL* variants

	Cohort	Total	Predicted deleterious biallelic *ITGAL* variants in EV versus Controls			
			N (%)	*P*-value	OR	Corrected OR [95% CI]
	EV (index cases)	40	4 (10%)	-	-	-
control cohorts	HGID (non-cHPV)	25,329	0 (0%)	8.06E-07	∞	442 [11-17,588]*
	Bacterial/Mycobacterial diseases	3,866	0 (0%)	2.80E-04	∞	57.07 [2.02-1612.0]*
	Fungal diseases	650	0 (0%)	2.77E-03	∞	32.6 [1-1020.5]*
	Viral encephalitis	1288	0 (0%)	1.19E-03	∞	43.9 [1.2-1547.2]*
	Viral pneumonia	4732	0 (0%)	1.10E-04	∞	91.4 [2.7-3094.4]*
	GnomAD v4	807,162	0 (0%)	2.00E-16	∞	199,026 [10,524-3,763,819]**

*, Firth-corrected odds ratio (OR), adjusted for ethnicity; **, Haldane-corrected OR.
N, number of cases; OR, odds ratio; ∞, infinite; EV, epidermodysplasia verruciformis; HGID, in-house cohorts; cHPV, cutaneous human papillomaviruses.
