# Supplementary material for: Human LFA-1 governs T cell immune surveillance of the skin
Source: Sci Immunol. Author manuscript; Available in PMC 2026 May 13. (PMC13171165; doi:10.1126/sciimmunol.adz8360)
Supplement: Supplementary Table 1 [file NIHMS2157577-supplement-Supplementary_Table_1.pdf]

Table S1. Genes with predicted deleterious homozygous variants identified in the EV cohort.

We included 40 index cases with no detected mutations in known EV-causing genes in this study. The filtering criteria are detailed in the Methods section.

Gene name	No. of Probands	Unique variants
ITGAL	4	3
ZFHX4	1	1
TAS2R10	1	1
RNASEH2B	1	1
ANXA5	1	1
ARHGEF33	1	1
UNC13C	1	1
CCDC113	1	1
PRRC2C	1	1
CCZ1	1	1
SBNO1	1	1
CIDEB	1	1
SSPN	1	1
CRTAP	1	1
ANK2	1	1
CYP2A6	1	1
VPS16	1	1
CYP2D6	1	1
ZNF225	1	1
FTL	1	1
PRSS12	1	1
GRIK1	1	1
RRP15	1	1
IQCH	1	1
SIGLEC1	1	1
SLC2A8	1	1
SLC6A13	1	1
SLC7A13	1	1
KCNA10	1	1
STAB1	1	1
KIF14	1	1
TBP	1	1
THSD1	1	1
TMEM38A	1	1
TPRX1	1	1
LRIT1	1	1
UNC79	1	1
METTL21A	1	1
ZC3H4	1	1
OLFML2B	1	1
ZNF16	1	1
PANX2	1	1
ADAM2	1	1
PIWIL3	1	1
44 genes	47 probands	46 unique variants
