# Supplementary material for: Human LFA-1 governs T cell immune surveillance of the skin
Source: Sci Immunol. Author manuscript; Available in PMC 2026 May 13. (PMC13171165; doi:10.1126/sciimmunol.adz8360)
Supplement: Supplementary Fig 9 [file NIHMS2157577-supplement-Supplementary_Fig_9.pdf]

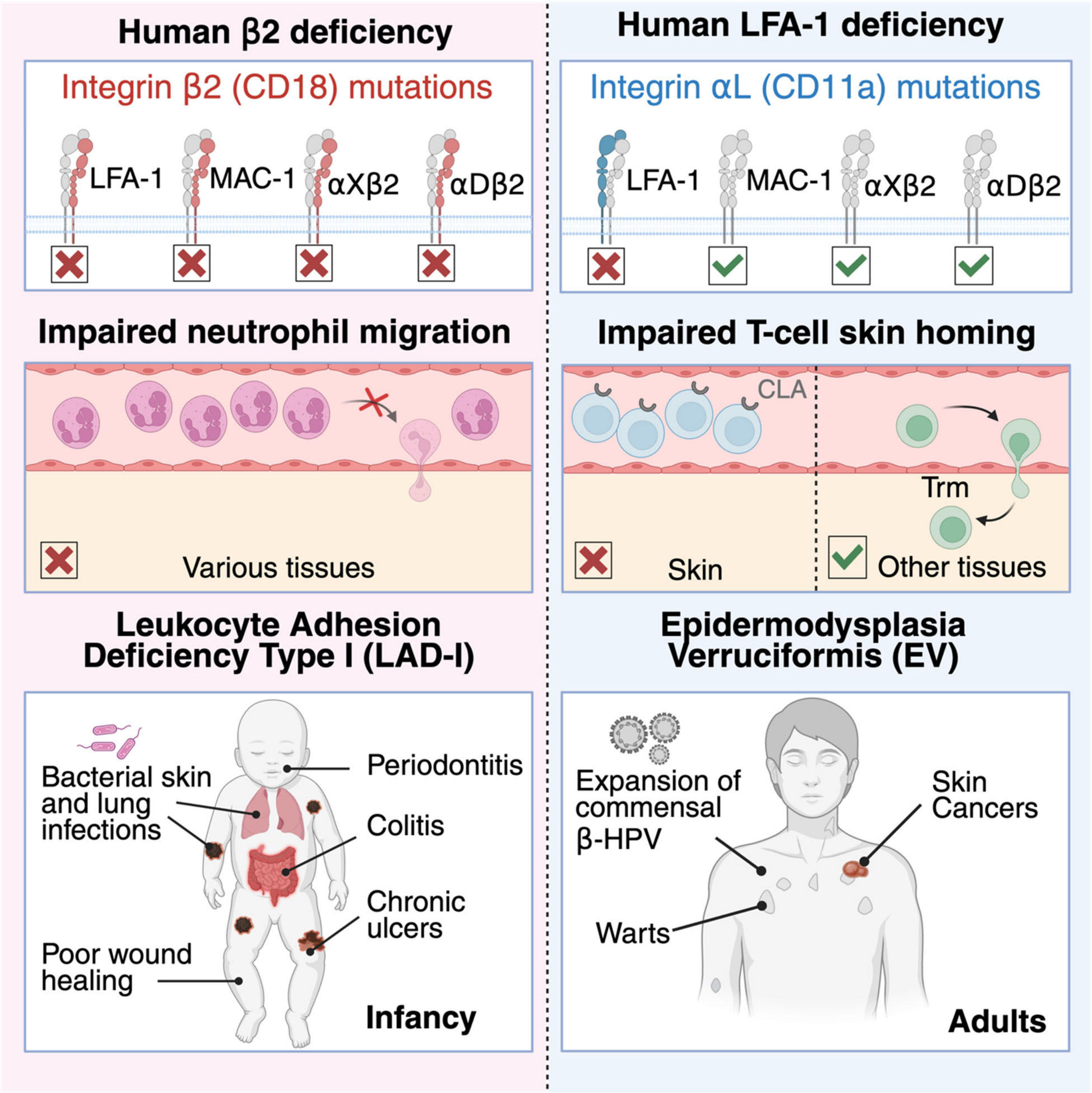

Figure S9. Human inherited integrin β2 (CD18) and integrin αL (CD11a) deficiencies

Inherited β2 (*ITGB2*/CD18) deficiency disrupts all β2 integrins, impairing neutrophil extravasation into inflamed tissues and causing early-onset susceptibility to bacterial and fungal infections in various organs. Inherited αL (*ITGAL*/CD11a) deficiency selectively disrupts LFA-1, impairing steady-state homing of skin-tropic memory T cells to the skin and compromising T cell-mediated surveillance of cutaneous commensal HPVs and associated skin cancers. Created in BioRender.
